# Supplementary material for: Stille coupling via C–N bond cleavage
Source: Nat Commun. 2016 Sep 30;7:12937. doi: 10.1038/ncomms12937 (PMC5056441; doi:10.1038/ncomms12937)
Supplement: Supplementary Information — Supplementary Figures 1-75, Supplementary Tables 1-5, Supplementary Discussion, Supplementary Methods and Supplementary References. [file ncomms12937-s1.pdf]

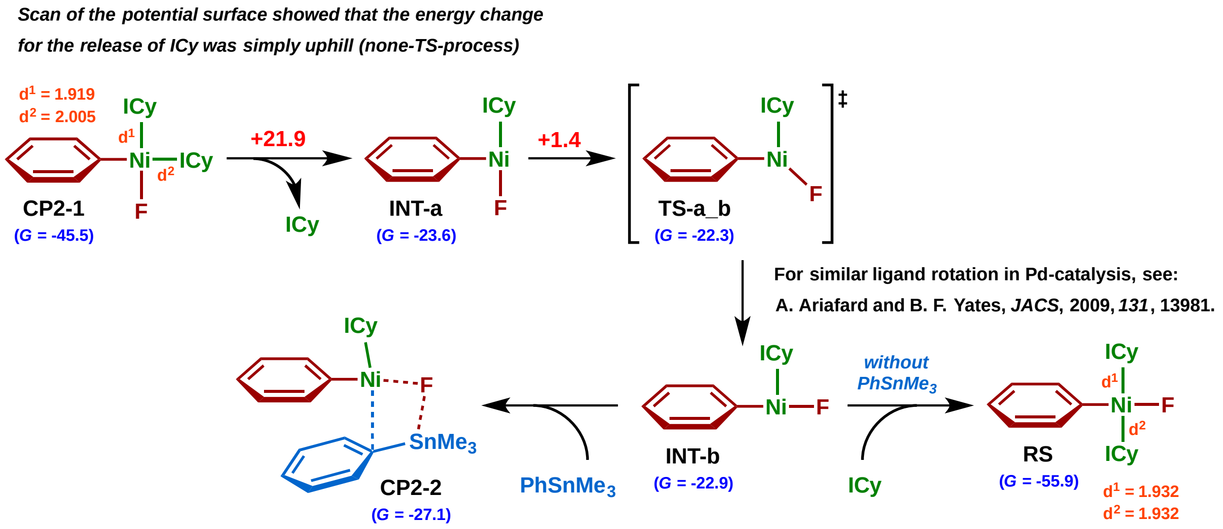

Supplementary Figure 1. Detailed Result in the Transmetalation Step from **CP2-1** to **CP2-2**.

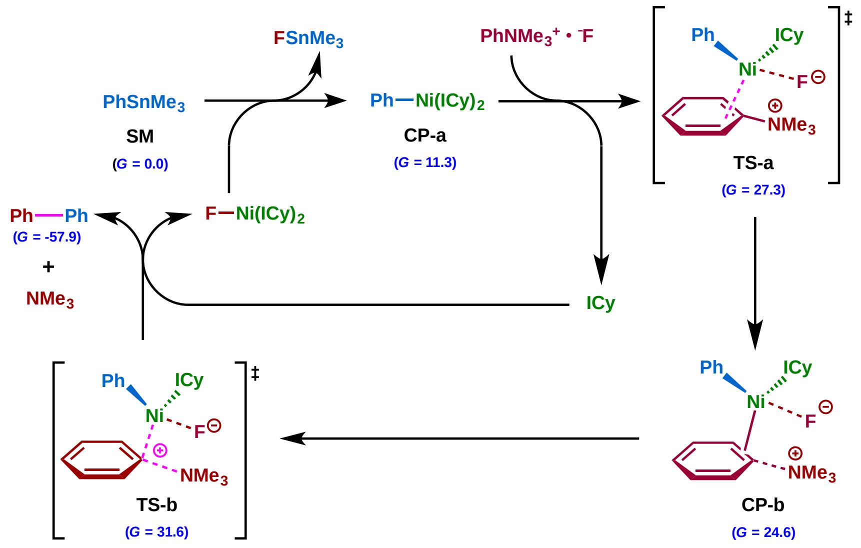

Supplementary Figure 2. Preliminary Calculation on the Ni(I)-mediated Mechanism (1): Reaction Route.

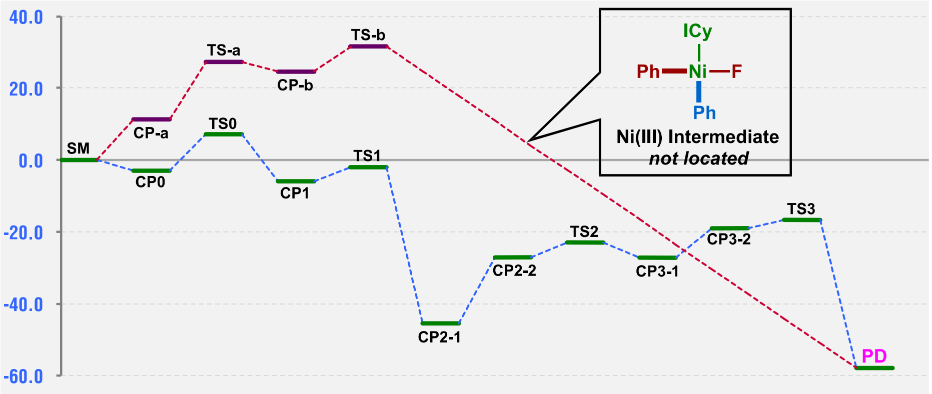

Supplementary Figure 3. Preliminary Calculation on the Ni(I)-mediated Mechanism (2): Energy Change.

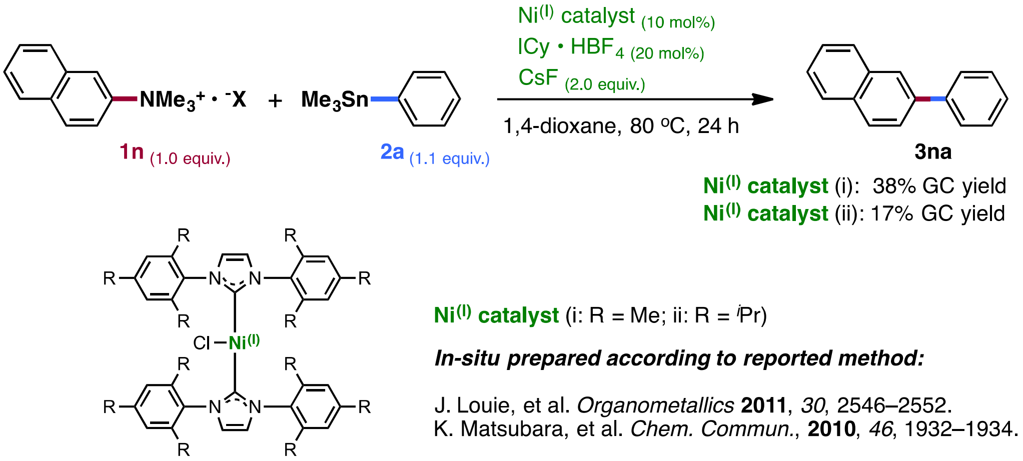

Supplementary Figure 4. Control Experiments with the Ni(I) Catalyst

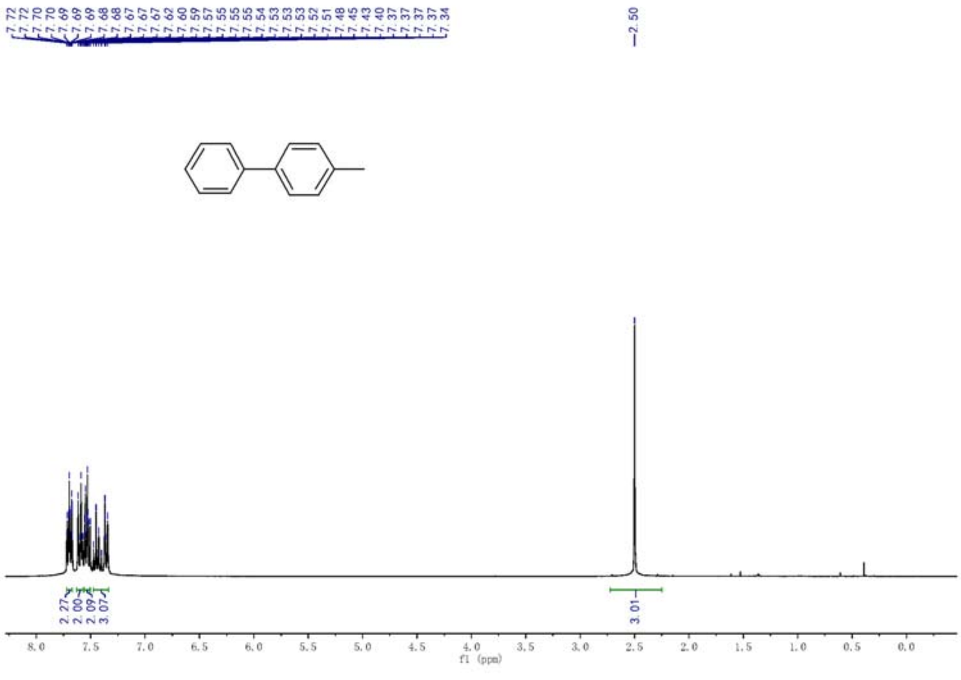

Supplementary Figure 5. ^{1}H NMR Spectrum of **3aa**

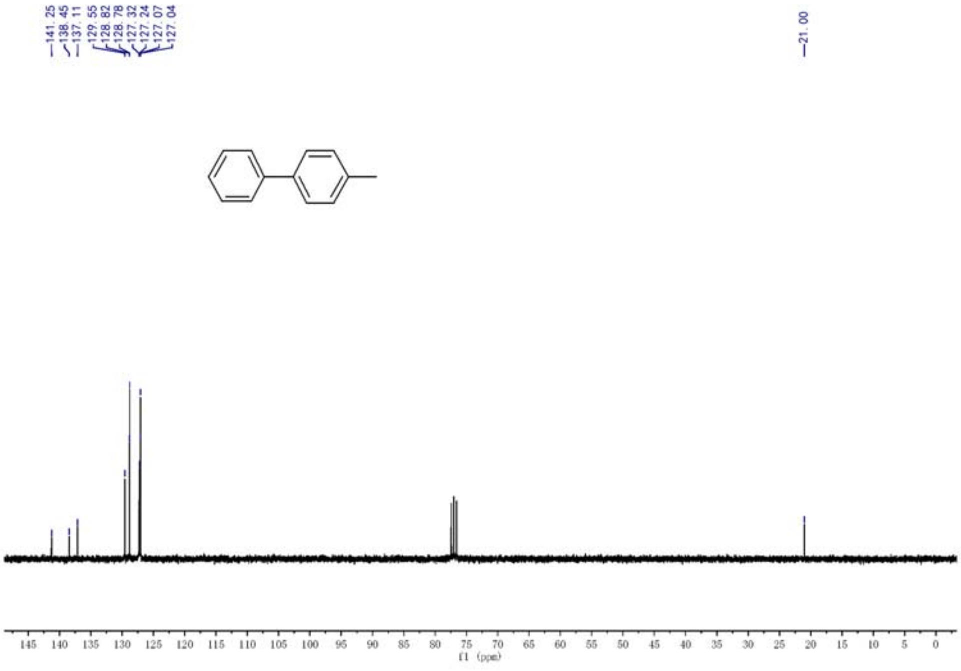

Supplementary Figure 6. ^{13}C NMR Spectrum of **3aa**

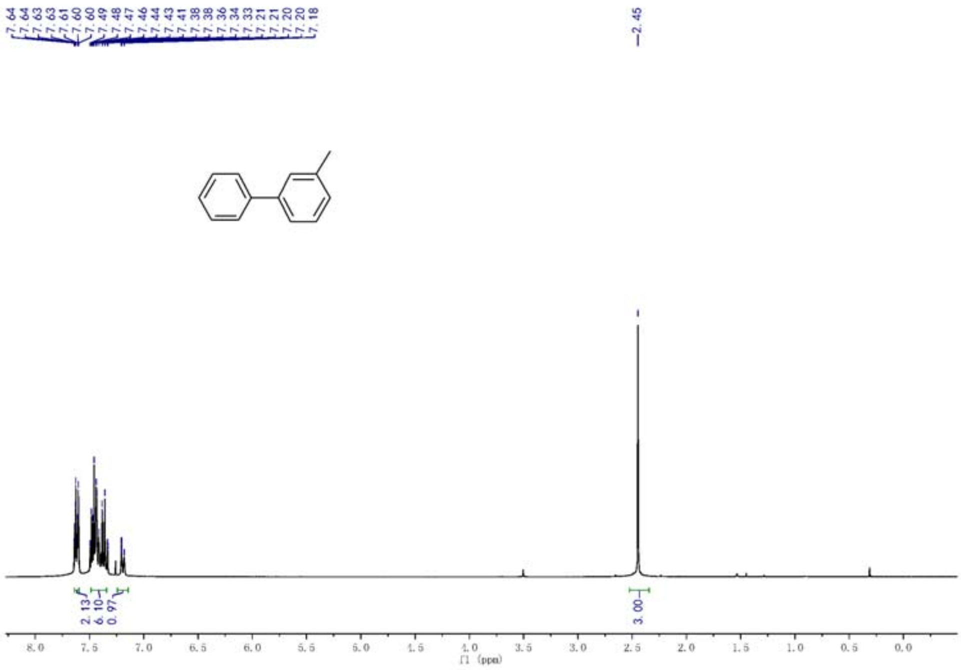

Supplementary Figure 7. ^{1}H NMR Spectrum of **3ba**

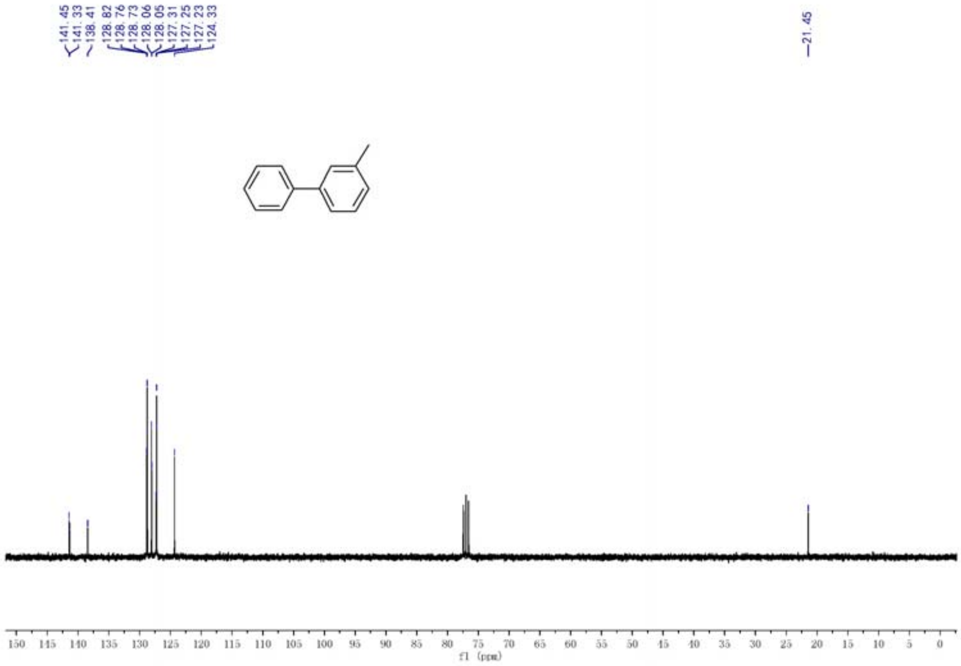

Supplementary Figure 8. ^{13}C NMR Spectrum of **3ba**

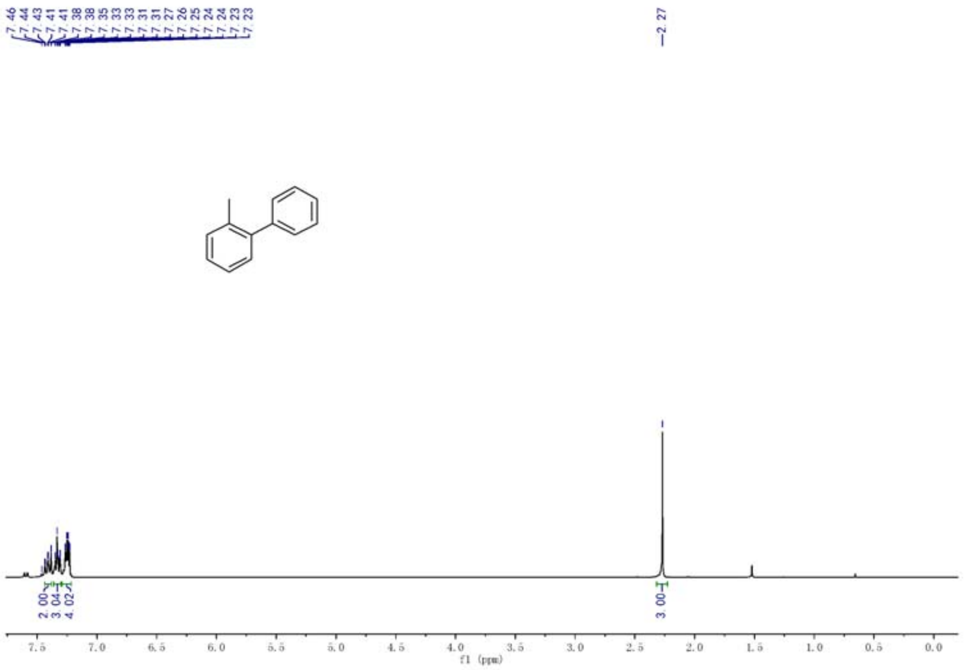

Supplementary Figure 9. ^{1}H NMR Spectrum of **3ca**

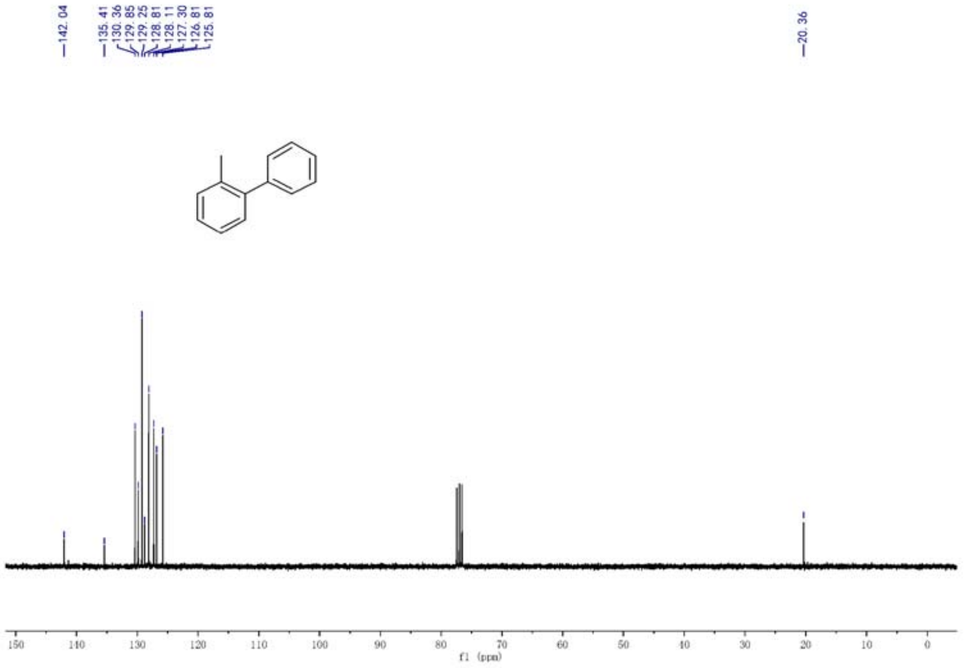

Supplementary Figure 10. ^{13}C NMR Spectrum of **3ca**

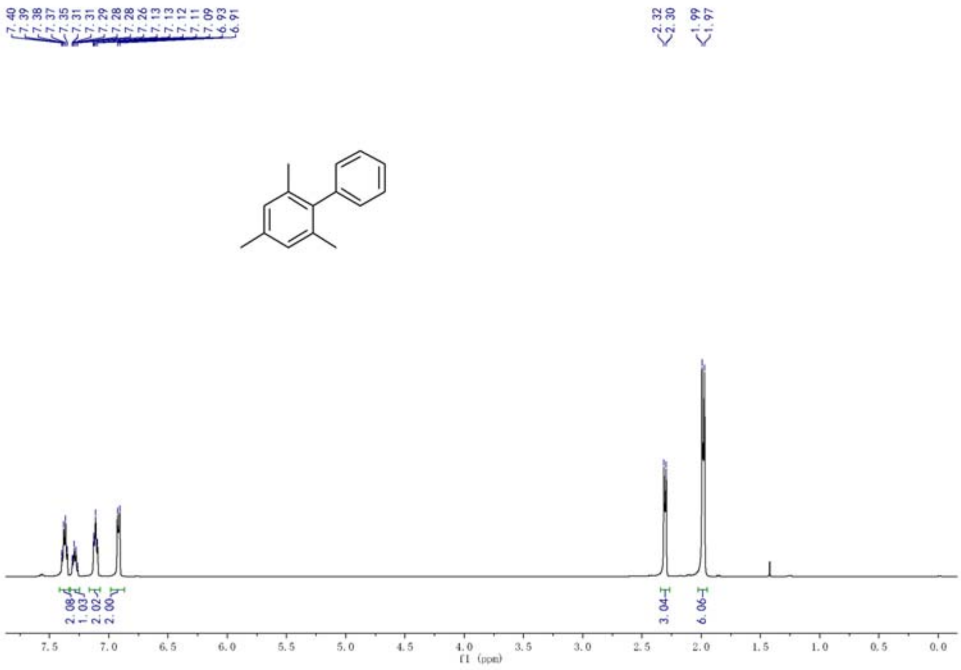

Supplementary Figure 11. ^{1}H NMR Spectrum of **3da**

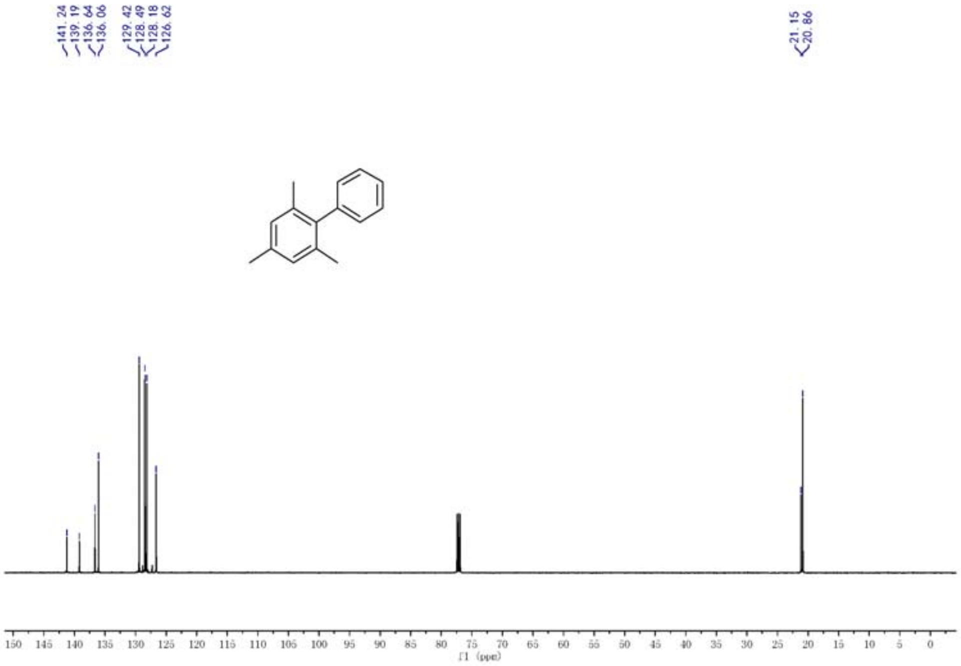

Supplementary Figure 12. ^{13}C NMR Spectrum of **3da**

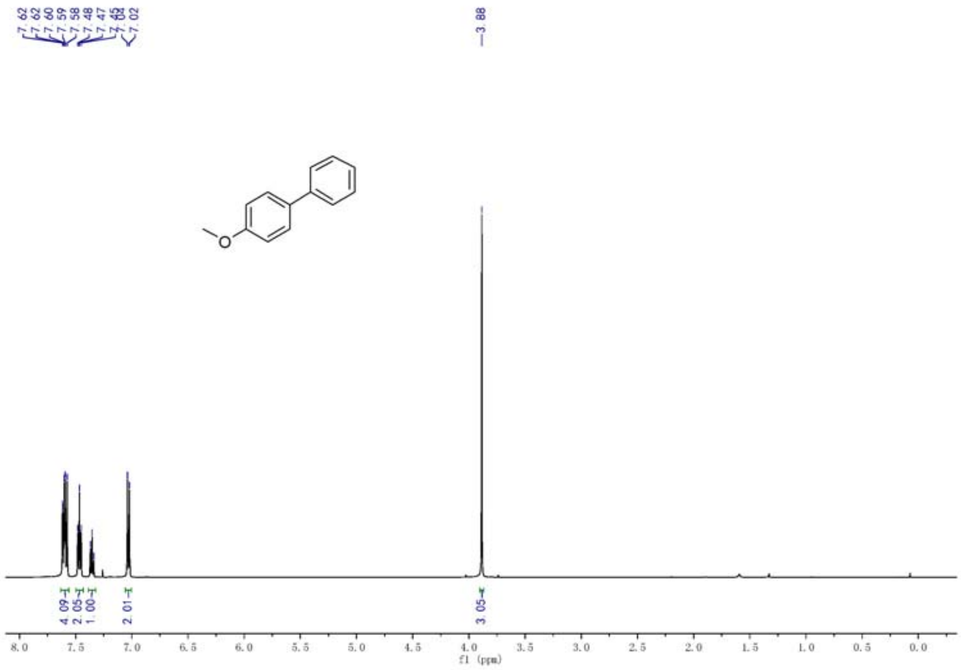

Supplementary Figure 13. ^{1}H NMR Spectrum of **3ea**

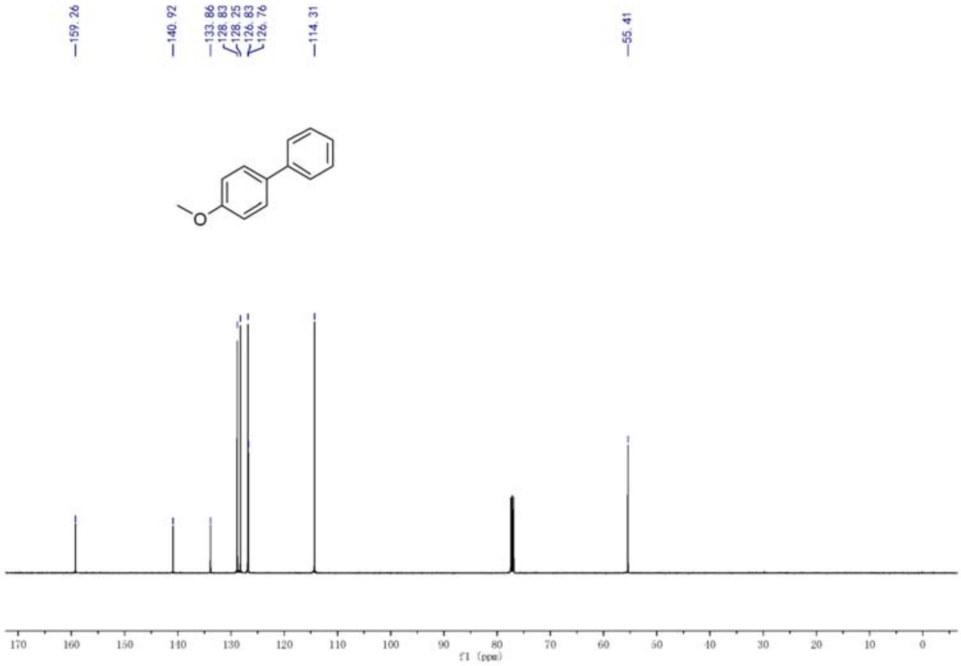

Supplementary Figure 14. ^{13}C NMR Spectrum of **3ea**

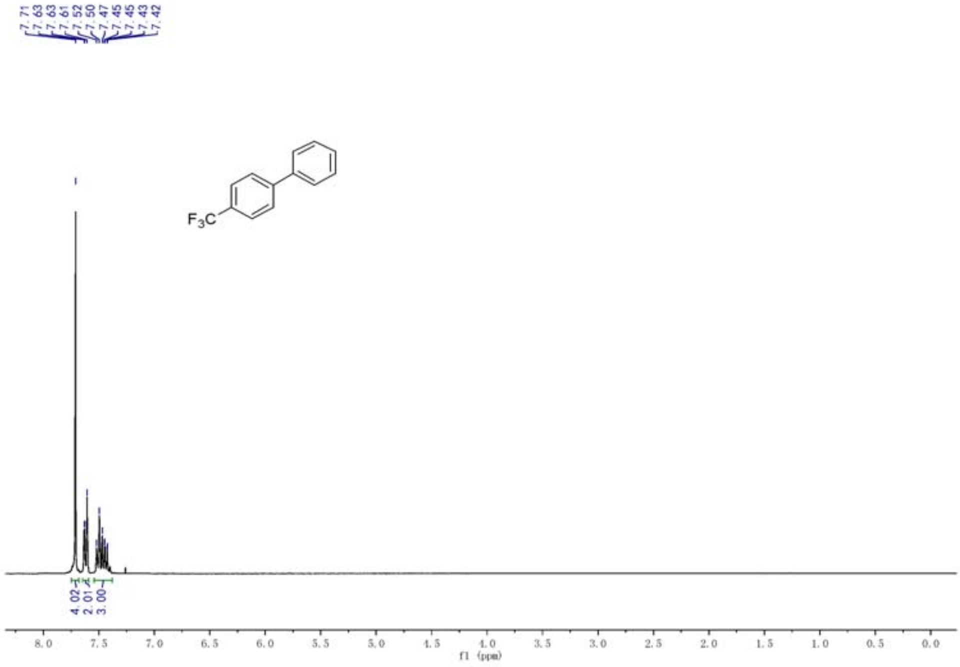

Supplementary Figure 15. ^{1}H NMR Spectrum of **3fa**

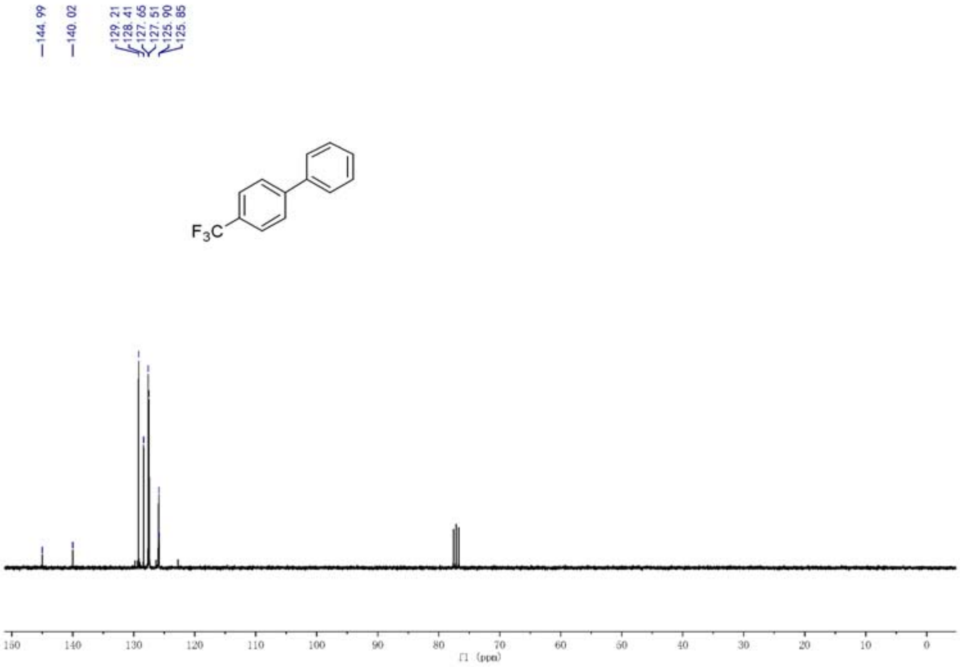

Supplementary Figure 16. ^{13}C NMR Spectrum of **3fa**

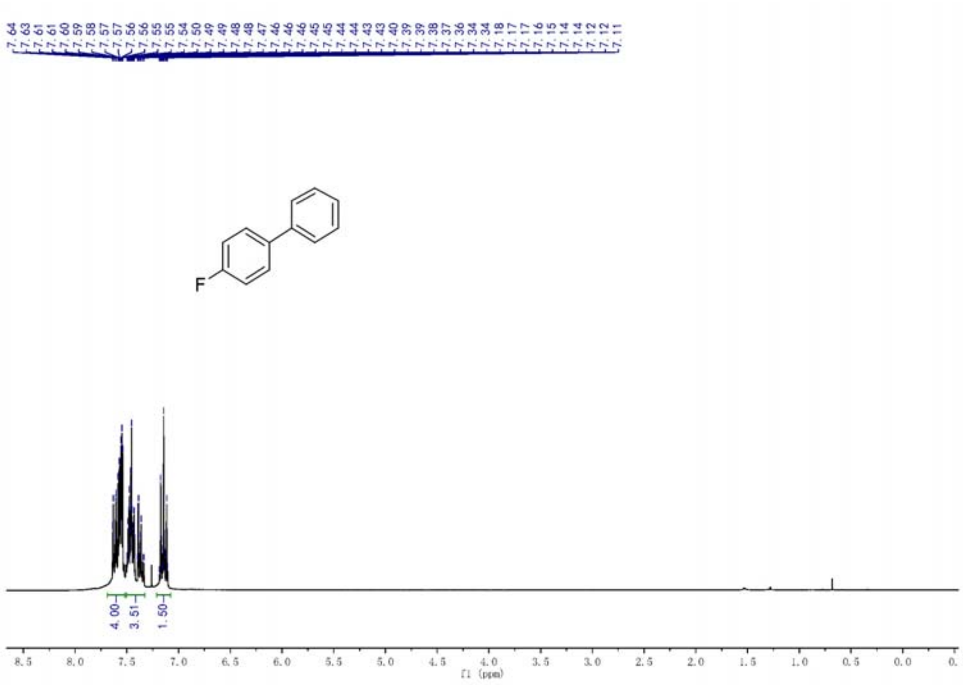

Supplementary Figure 17. ^{1}H NMR Spectrum of **3ga**

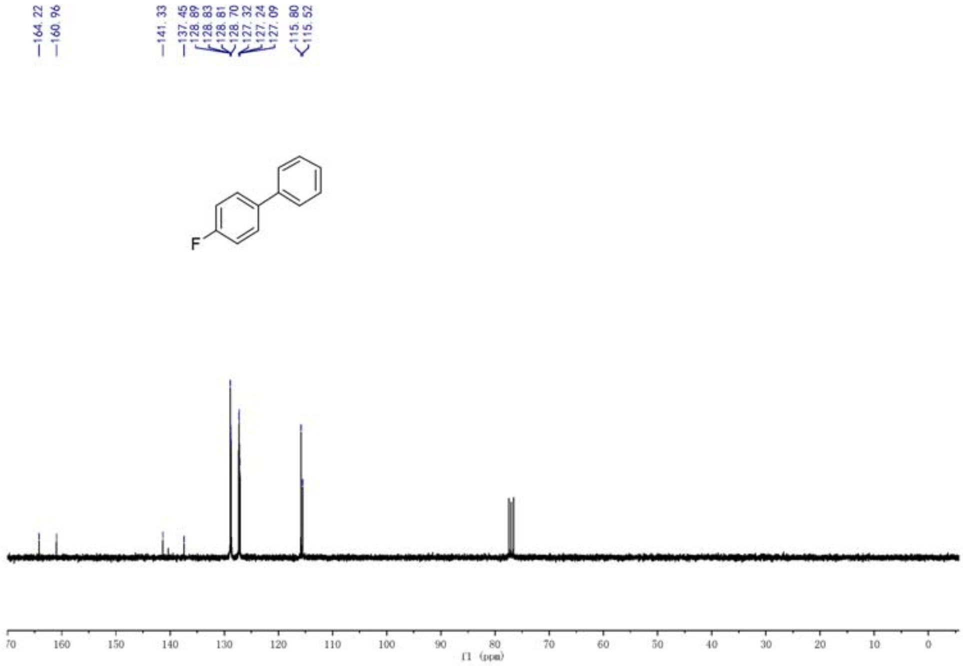

Supplementary Figure 18. ^{13}C NMR Spectrum of **3ga**

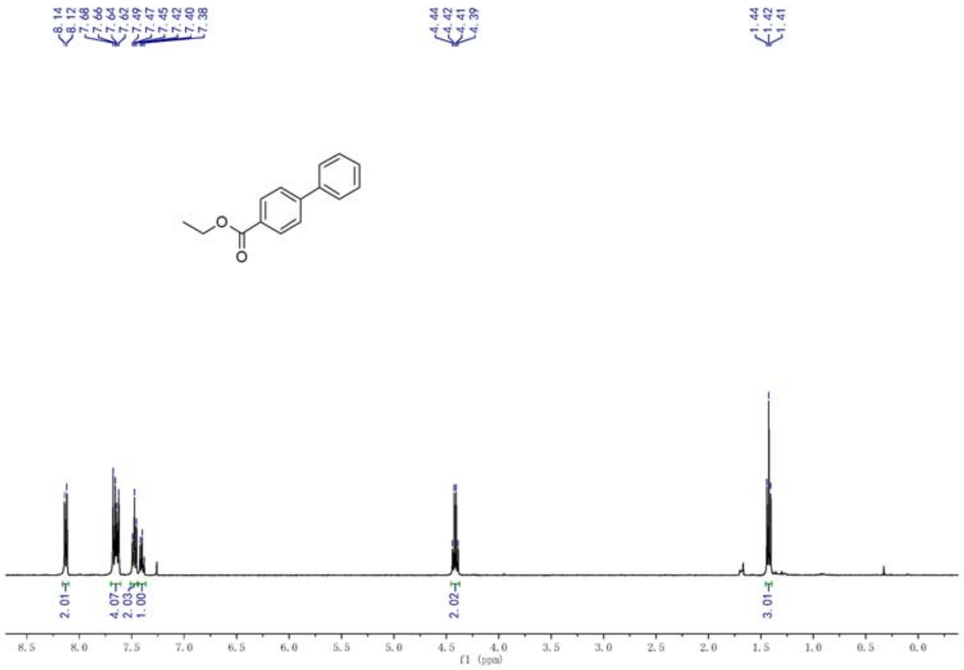

Supplementary Figure 19. ^{1}H NMR Spectrum of **3ha**

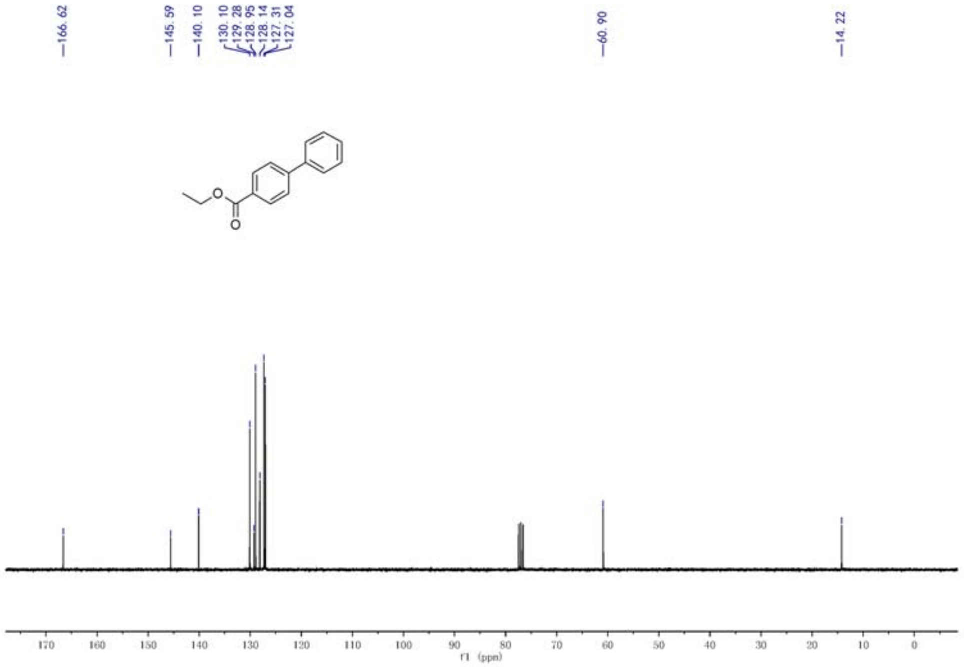

Supplementary Figure 20. ^{13}C NMR Spectrum of **3ha**

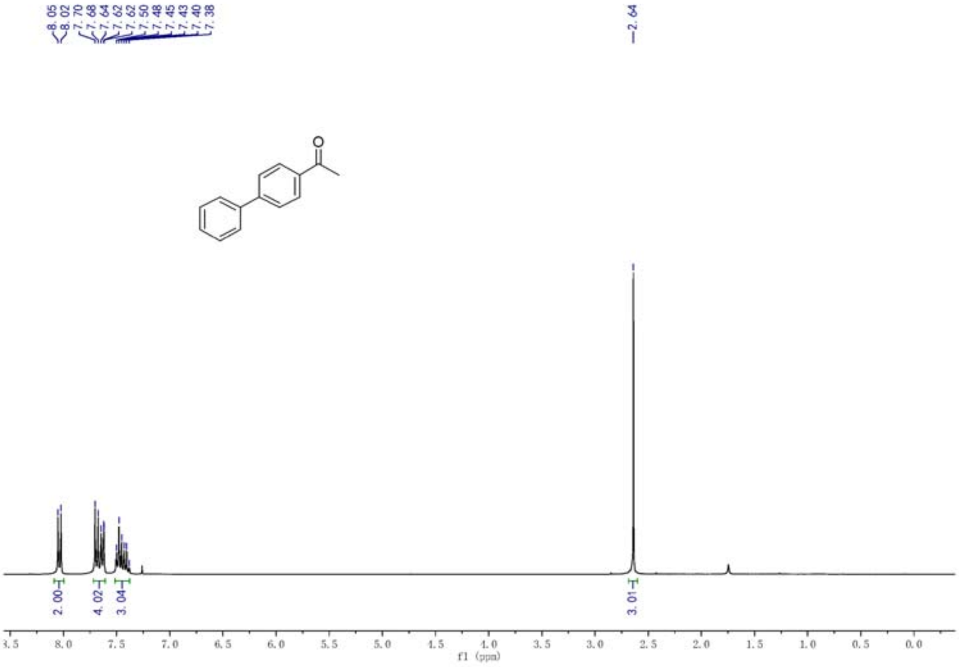

Supplementary Figure 21. ^{1}H NMR Spectrum of **3ia**

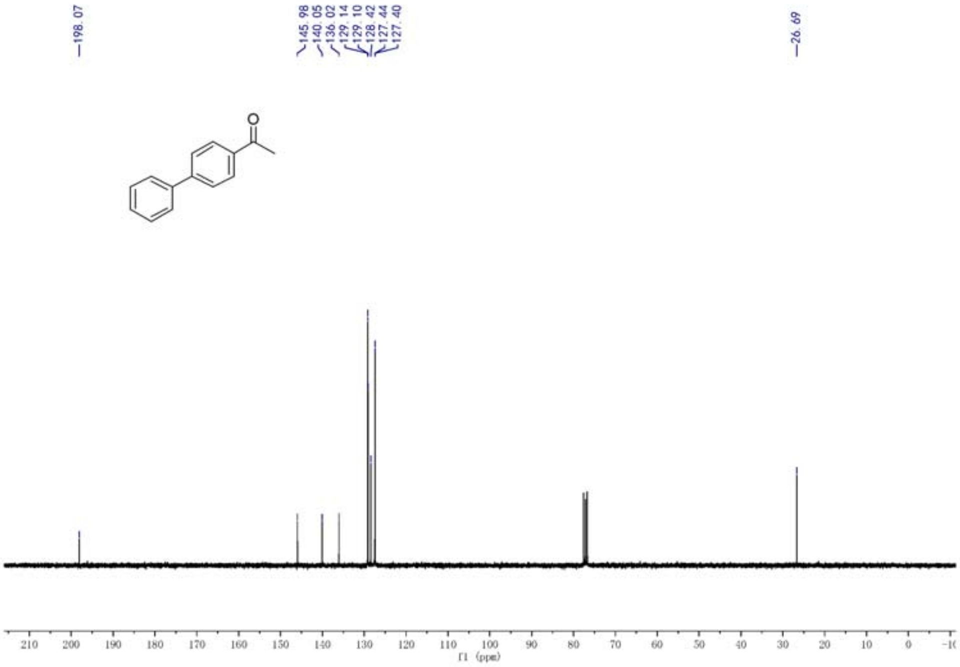

Supplementary Figure 22. ^{13}C NMR Spectrum of **3ia**

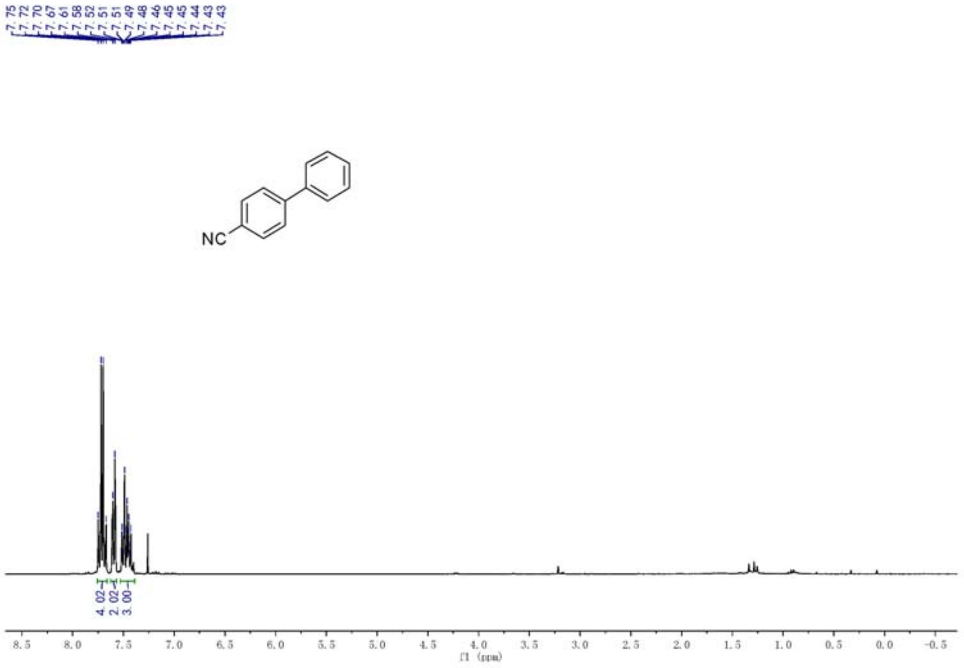

Supplementary Figure 23. ^{1}H NMR Spectrum of **3ja**

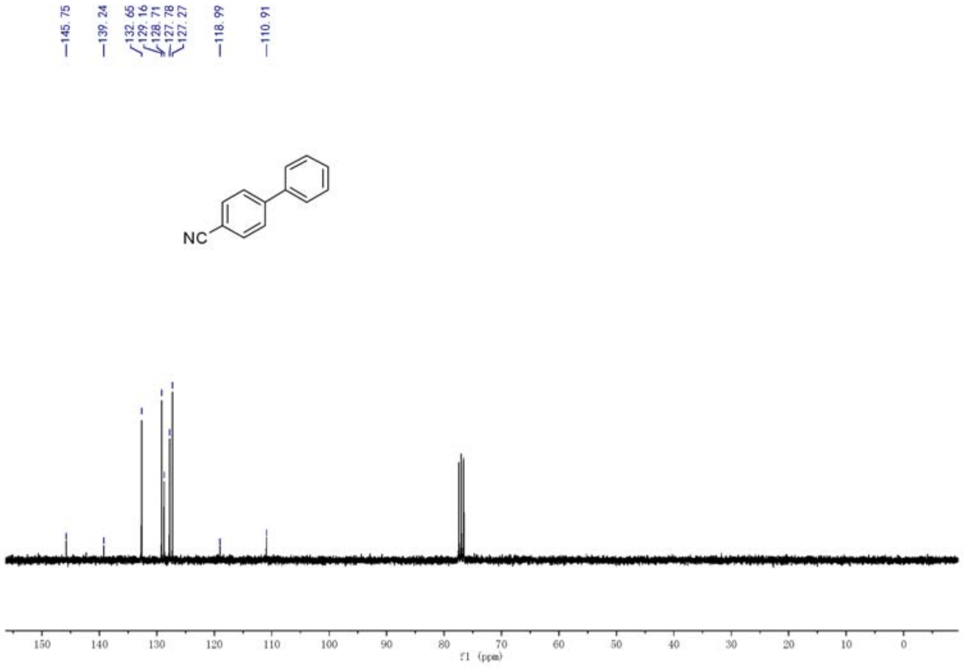

Supplementary Figure 24. ^{13}C NMR Spectrum of **3ja**

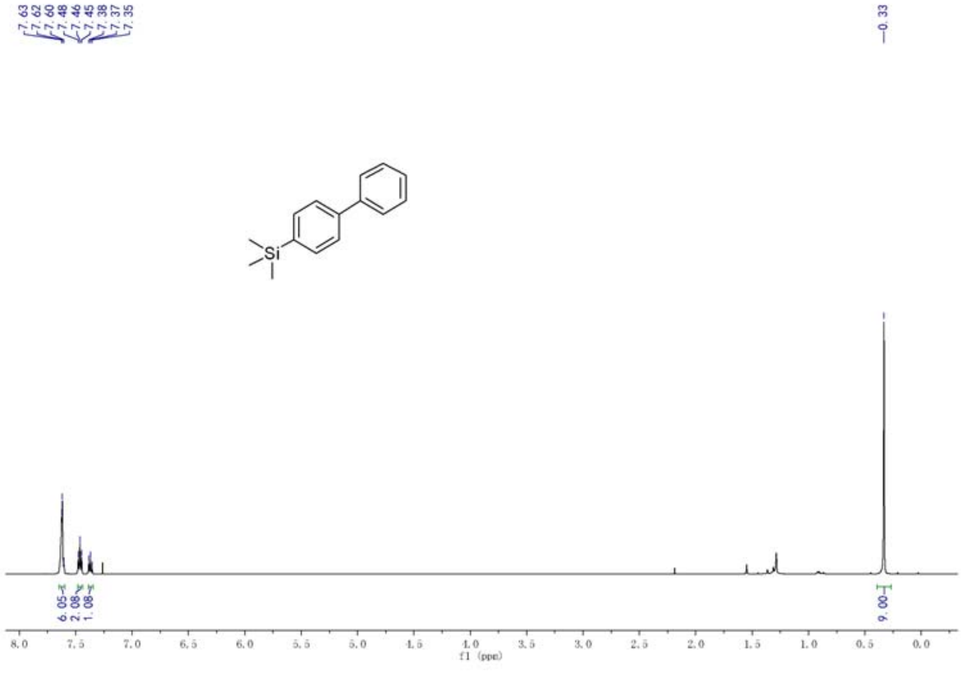

Supplementary Figure 25. ^{1}H NMR Spectrum of **3ka**

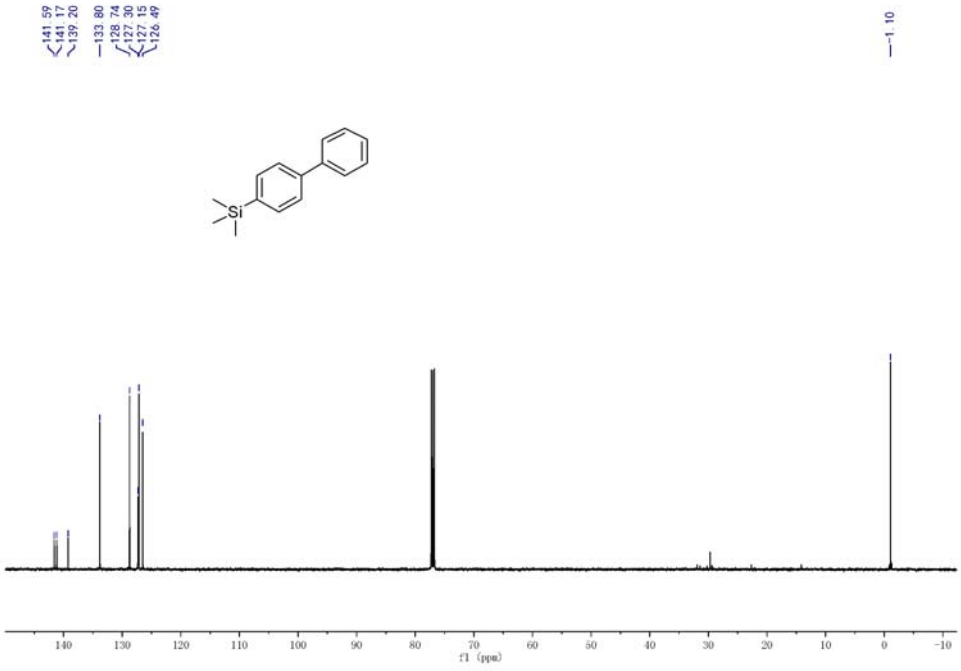

Supplementary Figure 26. ^{13}C NMR Spectrum of **3ka**

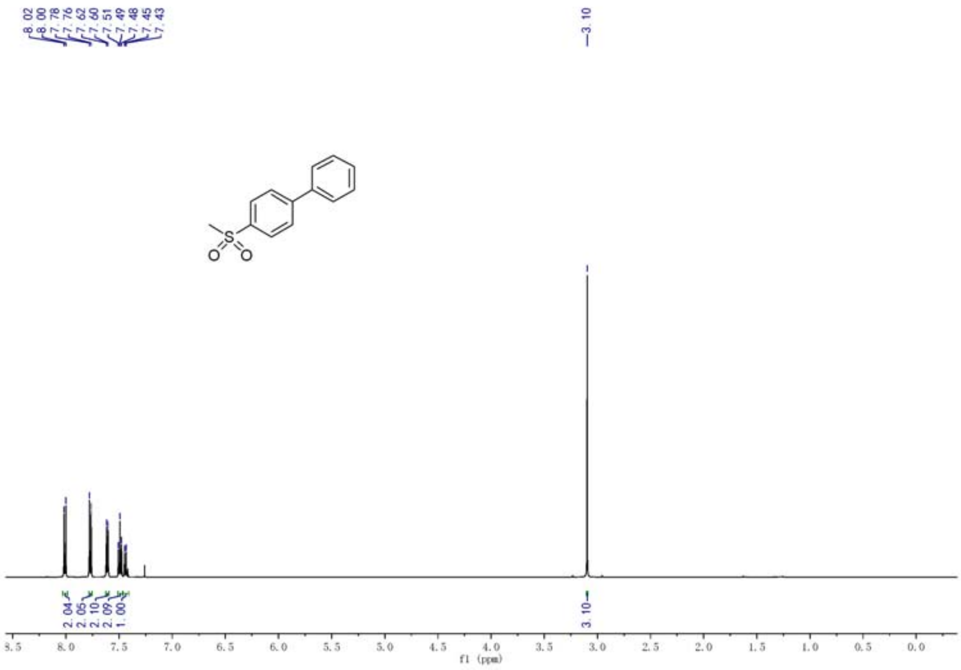

Supplementary Figure 27. ^{1}H NMR Spectrum of **3la**

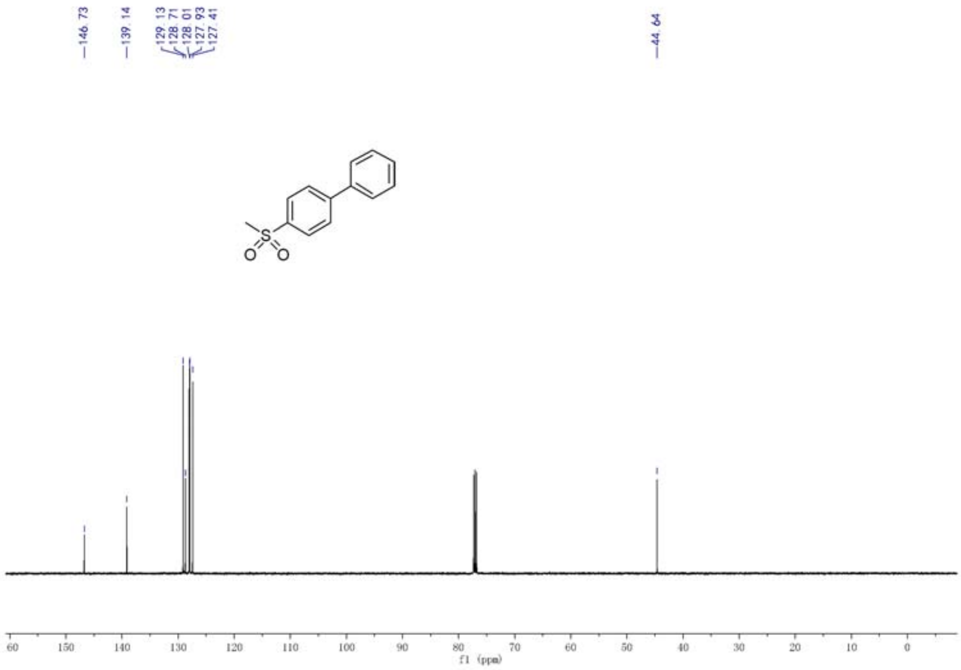

Supplementary Figure 28. ^{13}C NMR Spectrum of **3la**

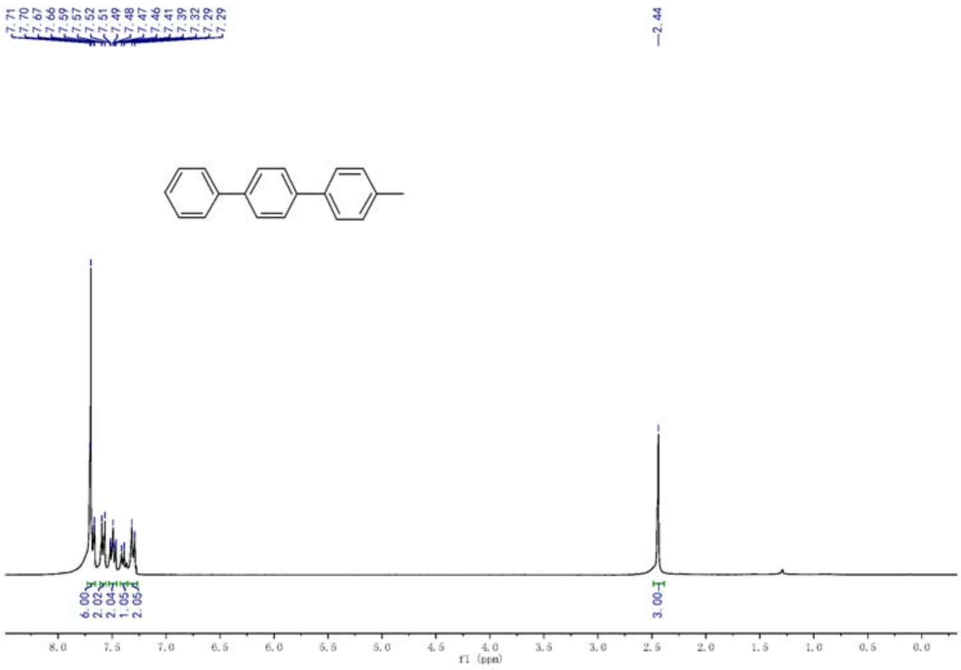

Supplementary Figure 29. ^{1}H NMR Spectrum of **3ma**

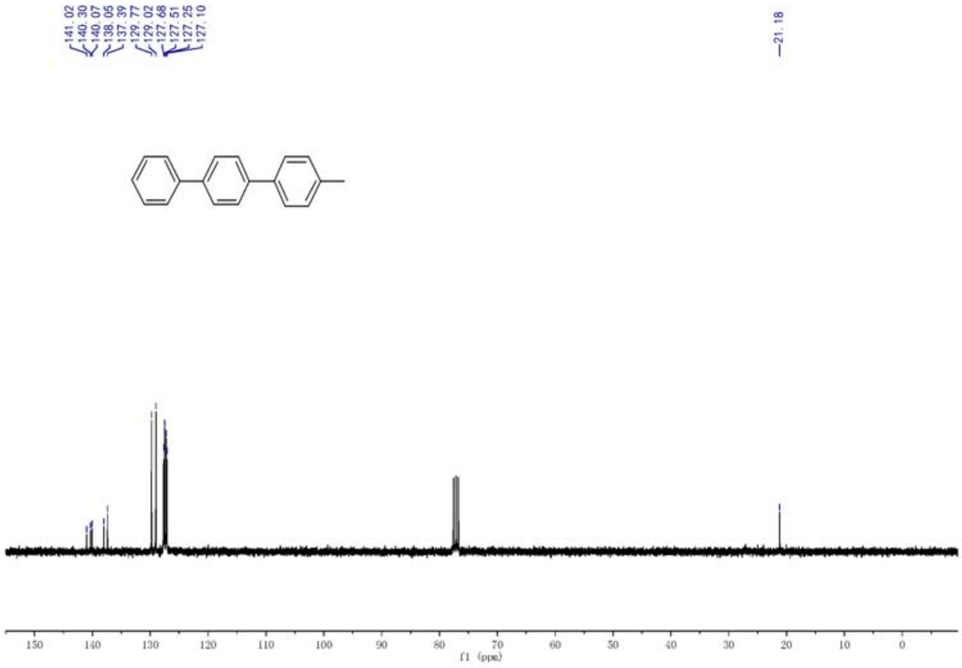

Supplementary Figure 30. ^{13}C NMR Spectrum of **3ma**

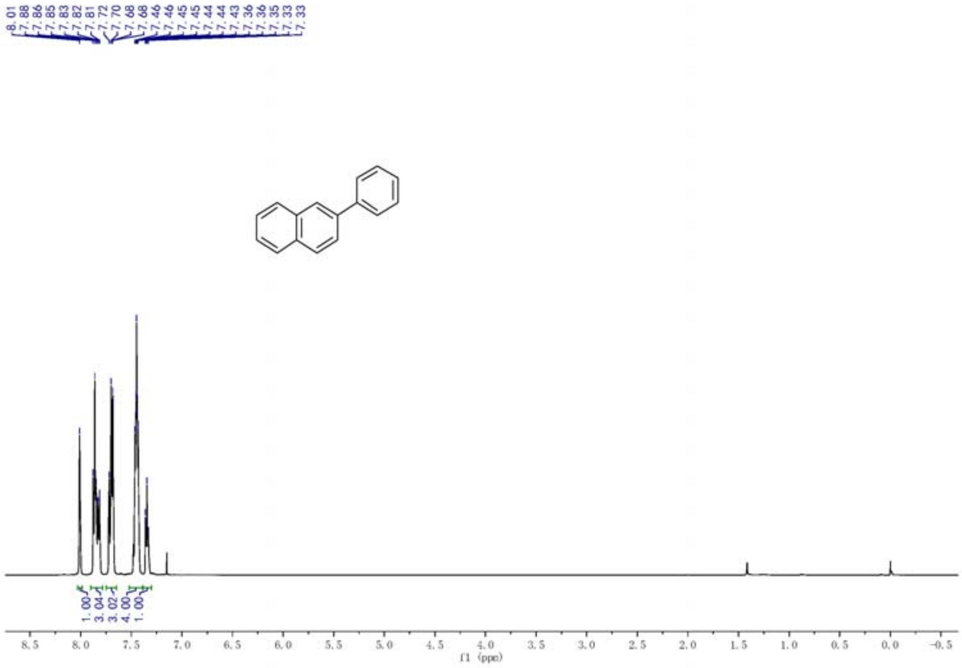

Supplementary Figure 31. ^{1}H NMR Spectrum of **3na**

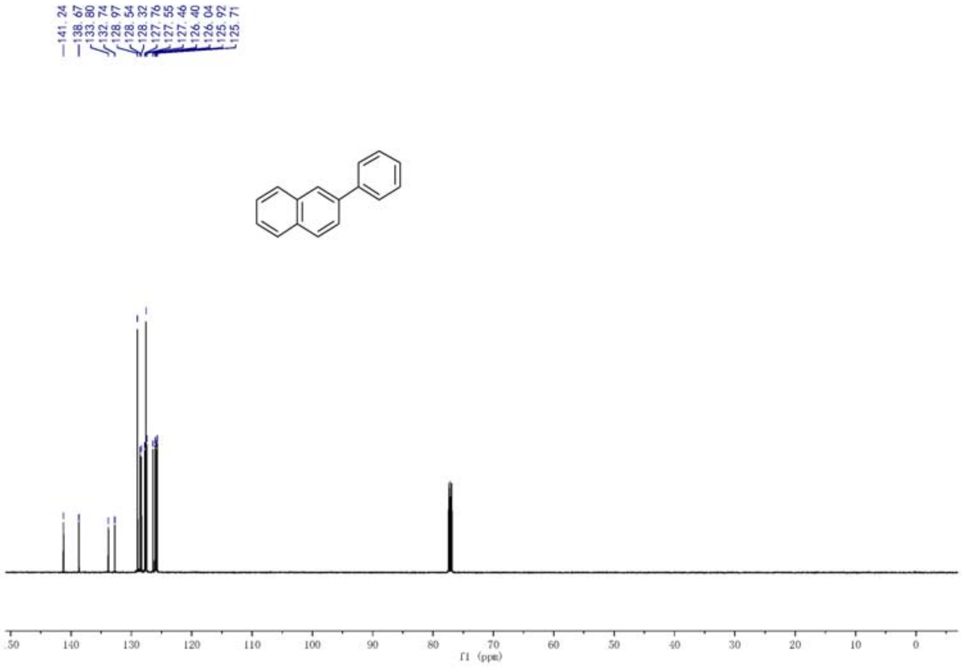

Supplementary Figure 32. ^{13}C NMR Spectrum of **3na**

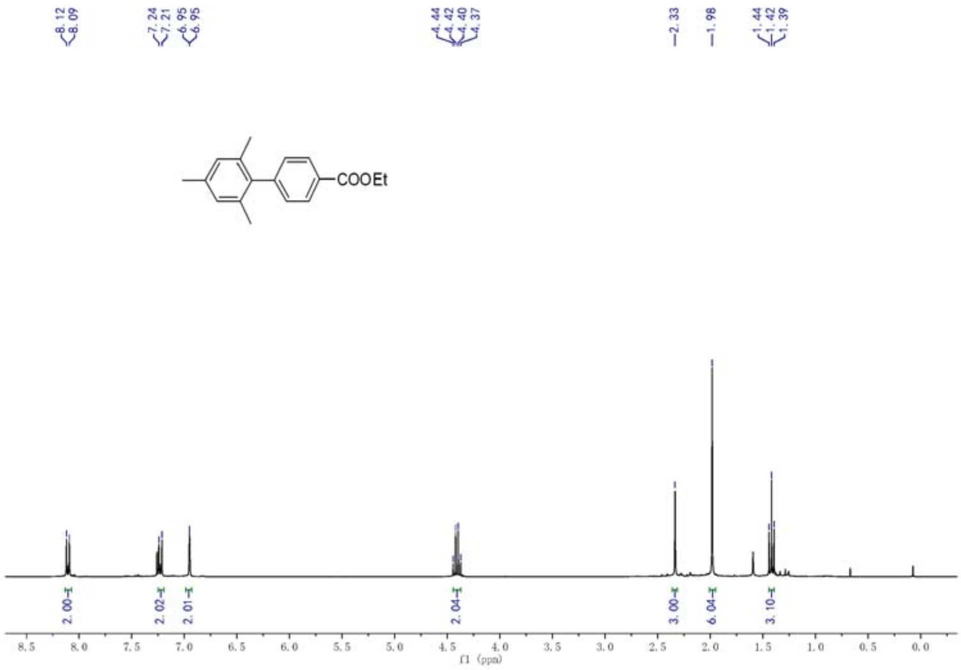

Supplementary Figure 33. ^{1}H NMR Spectrum of **3hb**

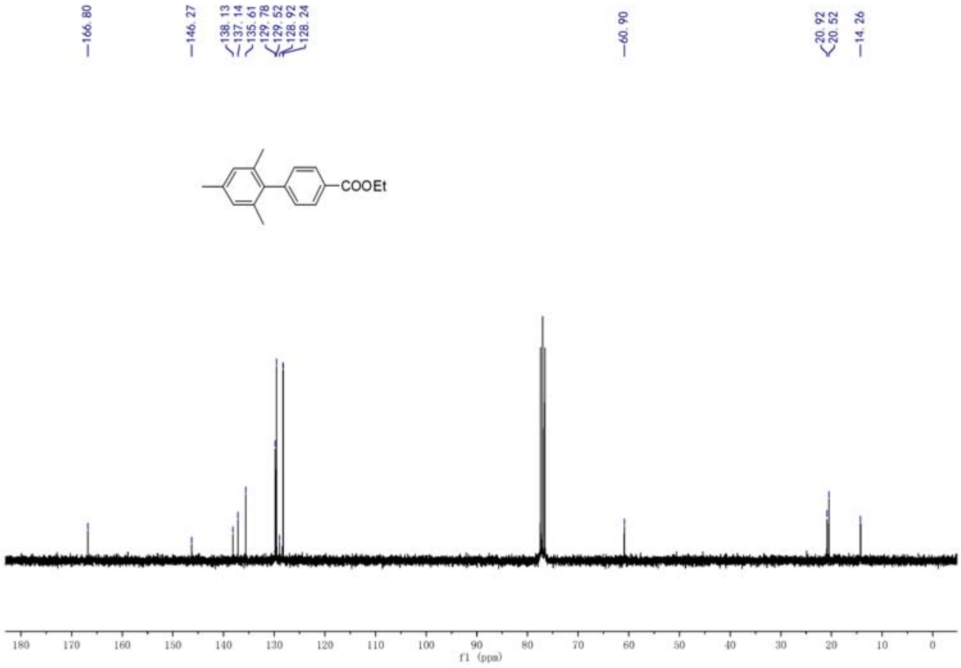

Supplementary Figure 34. ^{13}C NMR Spectrum of **3hb**

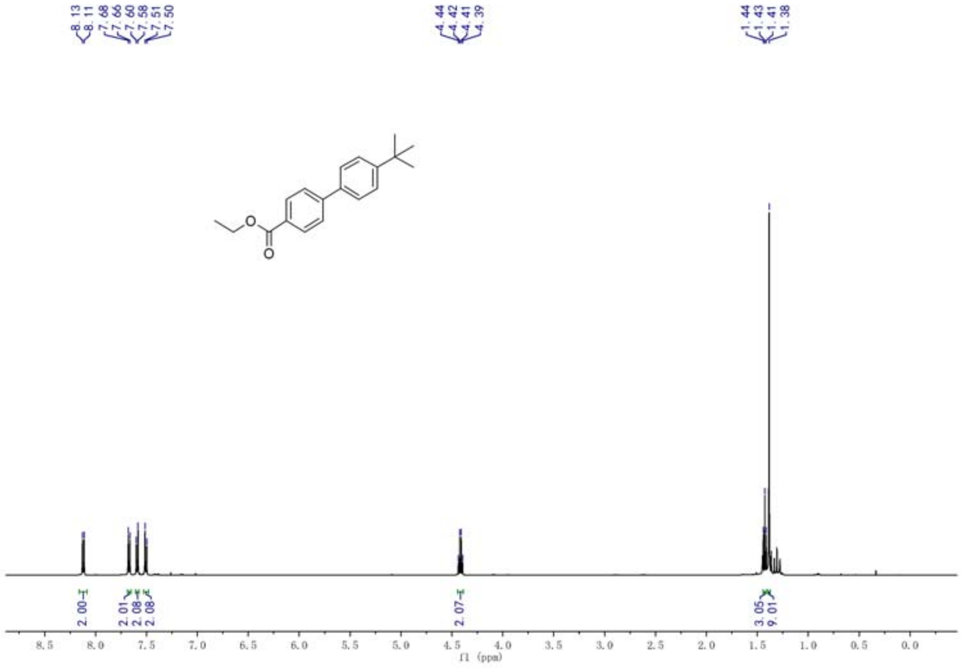

Supplementary Figure 35. ^{1}H NMR Spectrum of **3hc**

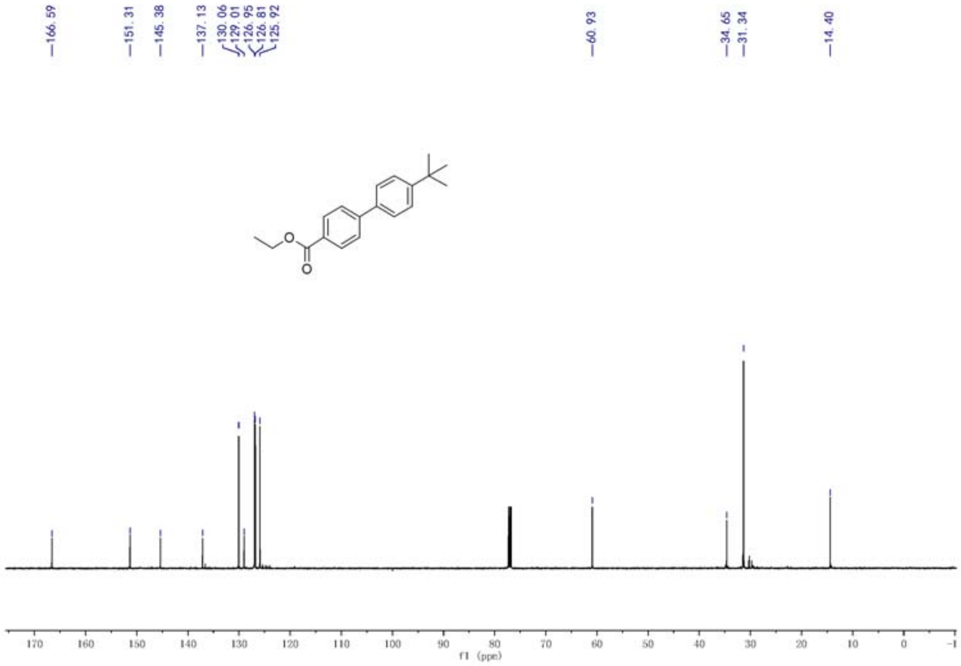

Supplementary Figure 36. ^{13}C NMR Spectrum of **3hc**

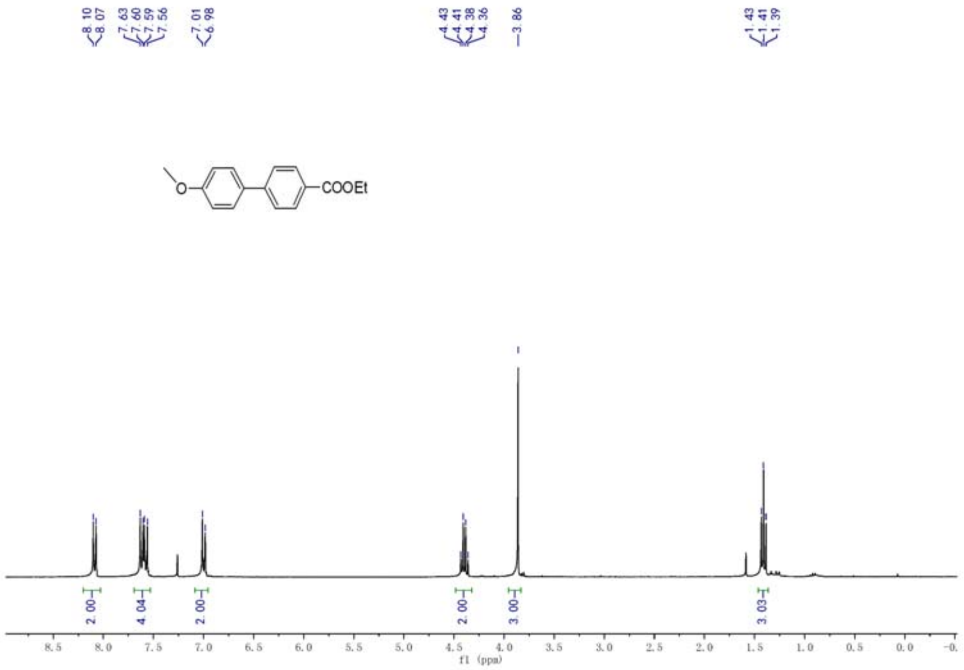

Supplementary Figure 37. ^{1}H NMR Spectrum of **3hd**

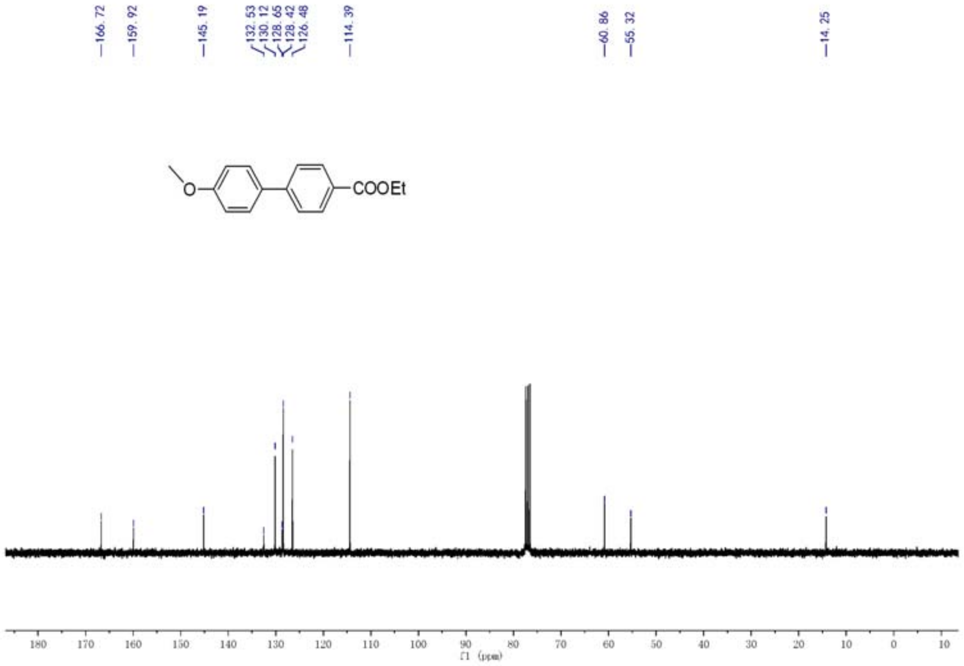

Supplementary Figure 38. ^{13}C NMR Spectrum of **3hd**

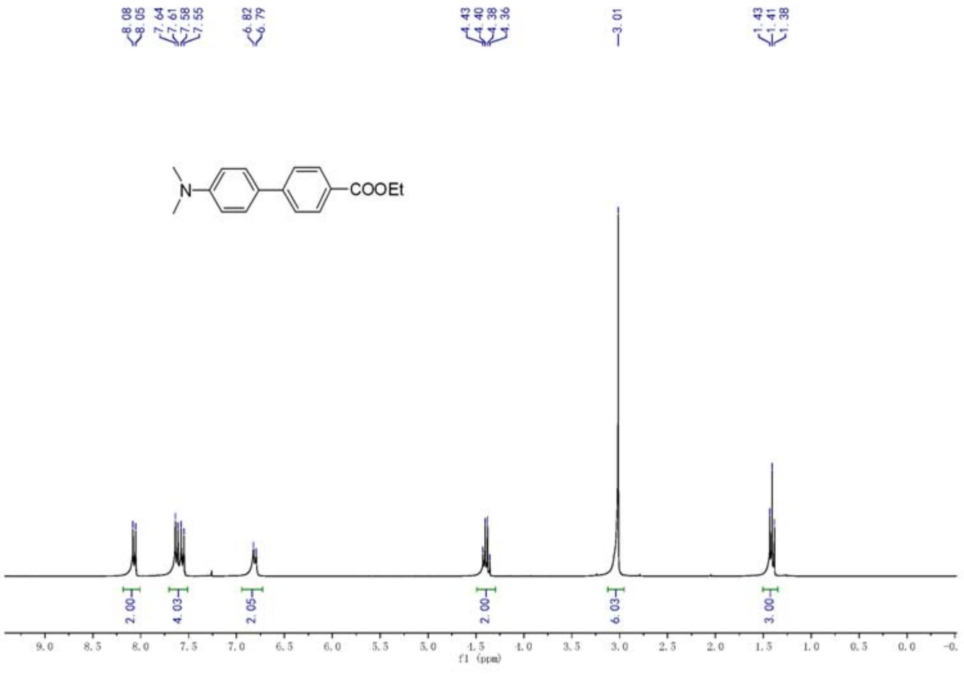

Supplementary Figure 39. ^{1}H NMR Spectrum of **3he**

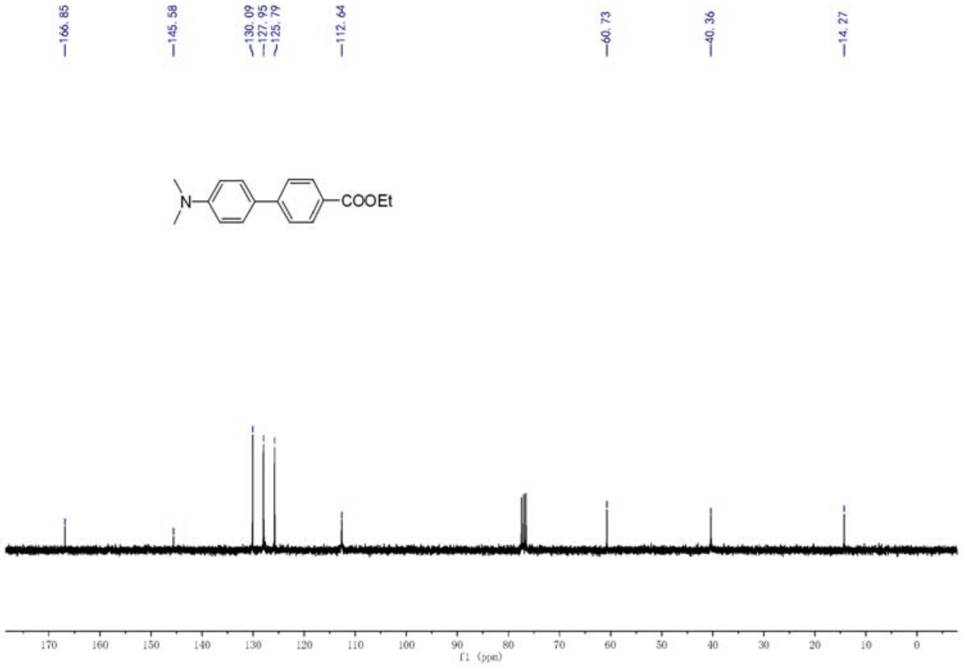

Supplementary Figure 40. ^{13}C NMR Spectrum of **3he**

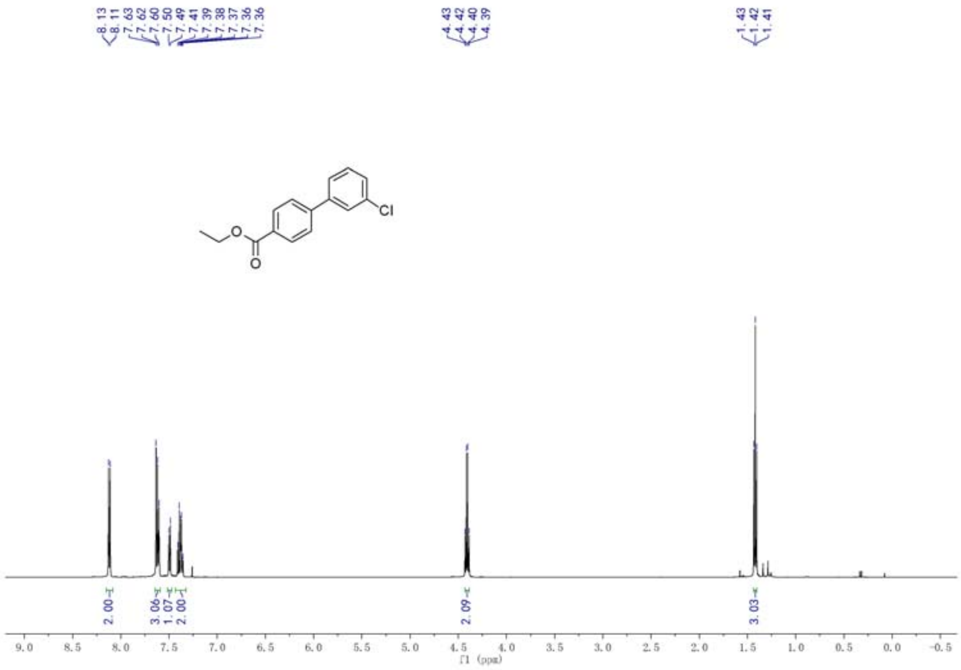

Supplementary Figure 41. ^{1}H NMR Spectrum of **3hg**

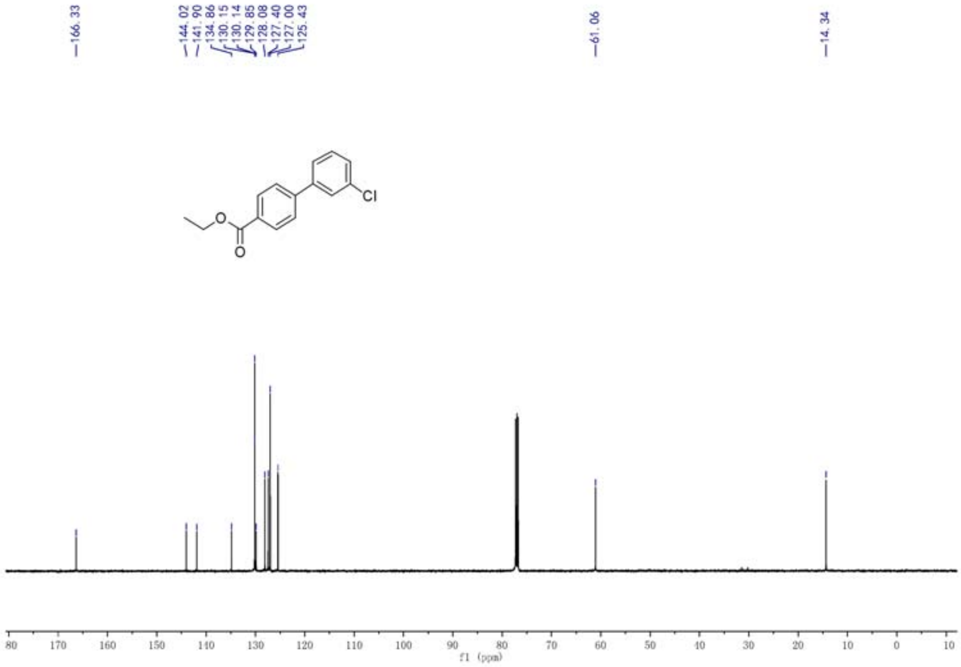

Supplementary Figure 42. ^{13}C NMR Spectrum of **3hg**

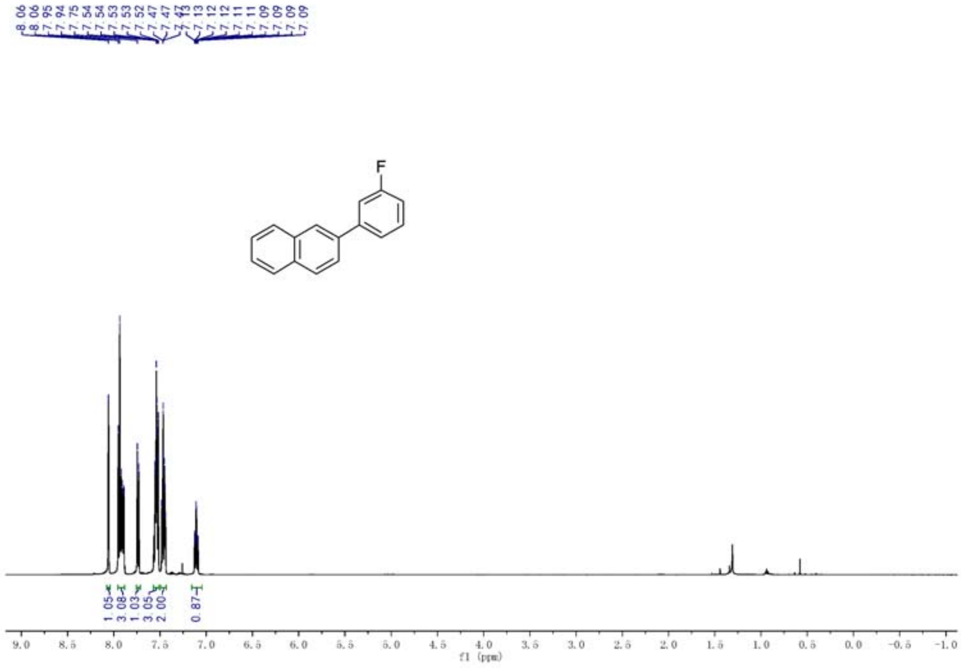

Supplementary Figure 43. ^{1}H NMR Spectrum of **3hf**

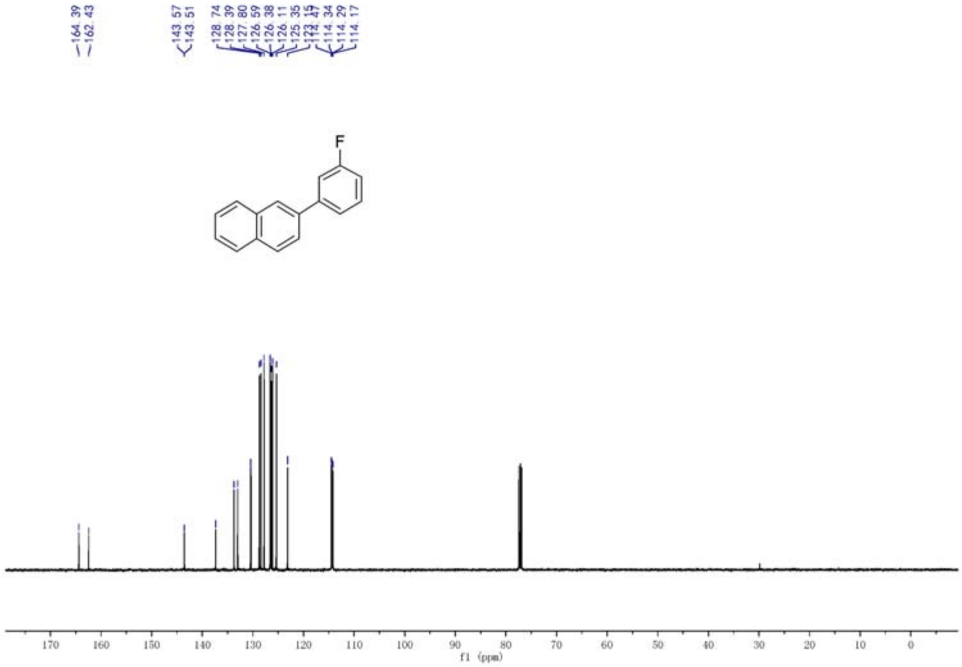

Supplementary Figure 44. ^{13}C NMR Spectrum of **3hf**

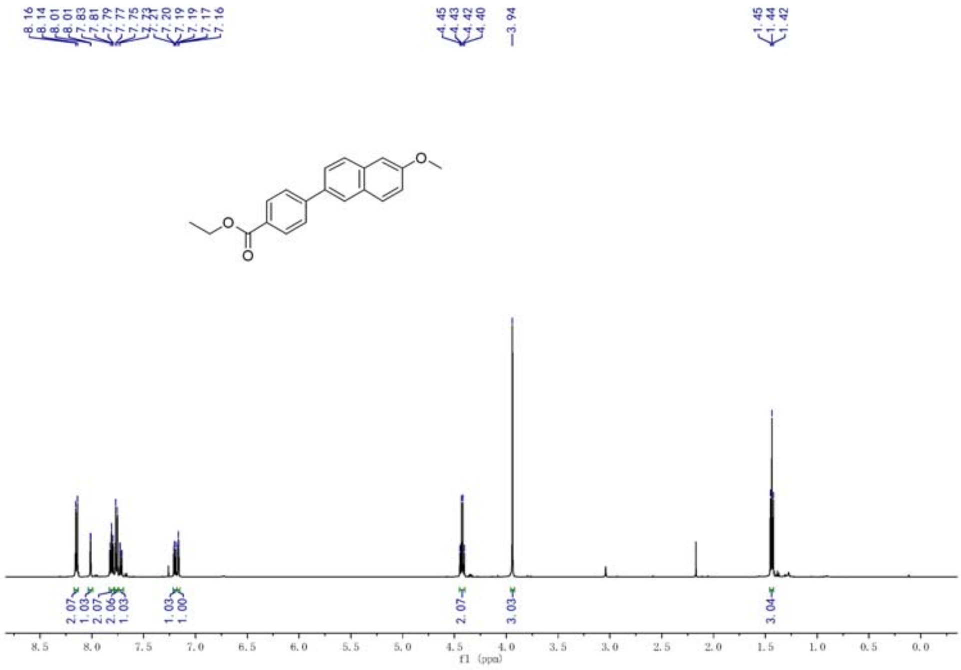

Supplementary Figure 45. ^{1}H NMR Spectrum of **3hh**

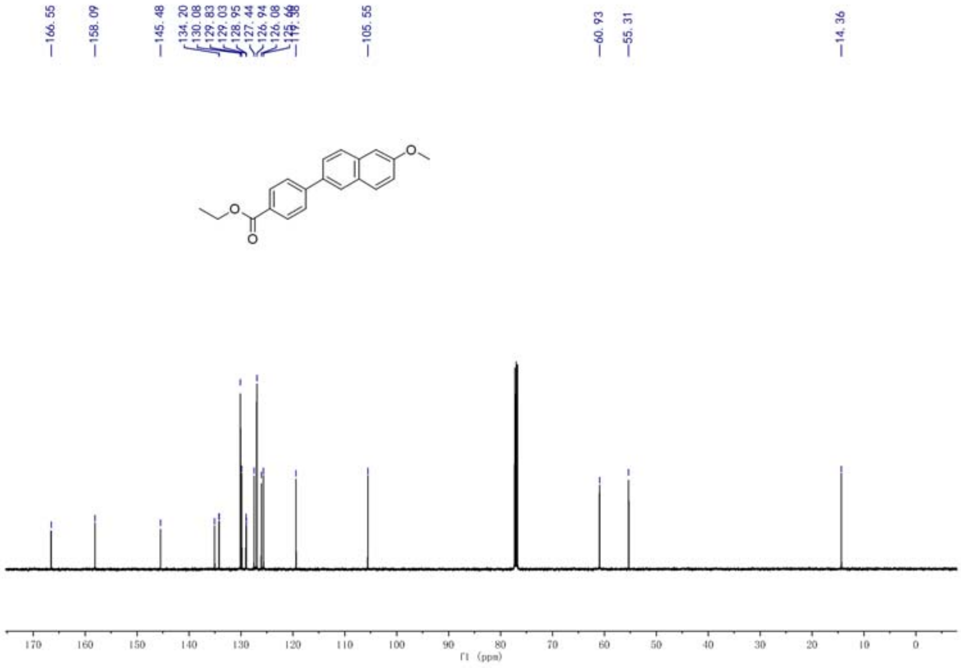

Supplementary Figure 46. ^{13}C NMR Spectrum of **3hh**

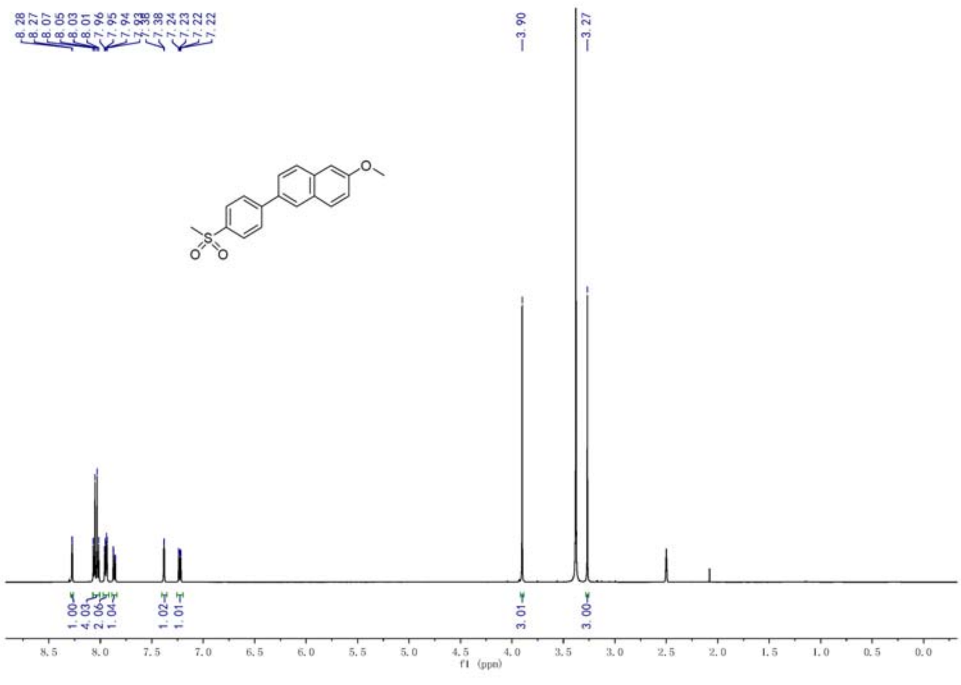

Supplementary Figure 47. ^{1}H NMR Spectrum of **3lh**

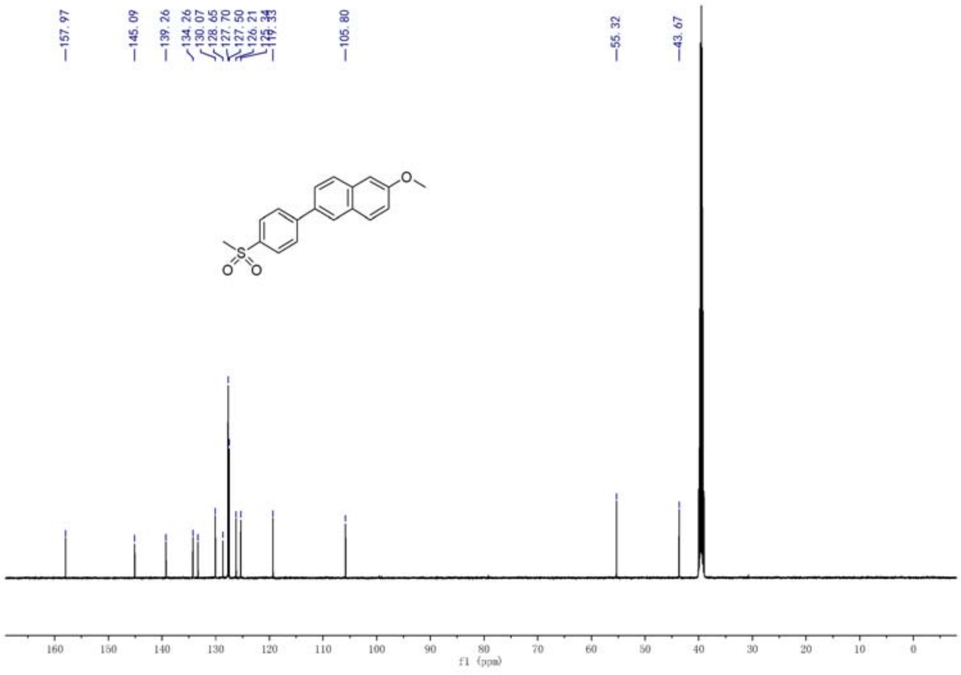

Supplementary Figure 48. ^{13}C NMR Spectrum of **3lh**

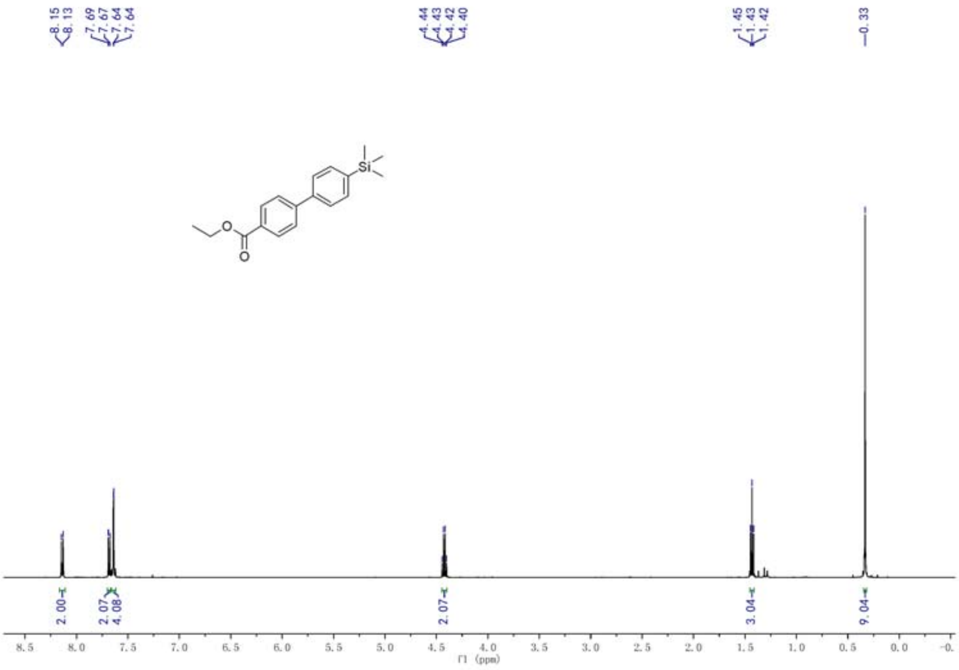

Supplementary Figure 49. ^{1}H NMR Spectrum of **3hi**

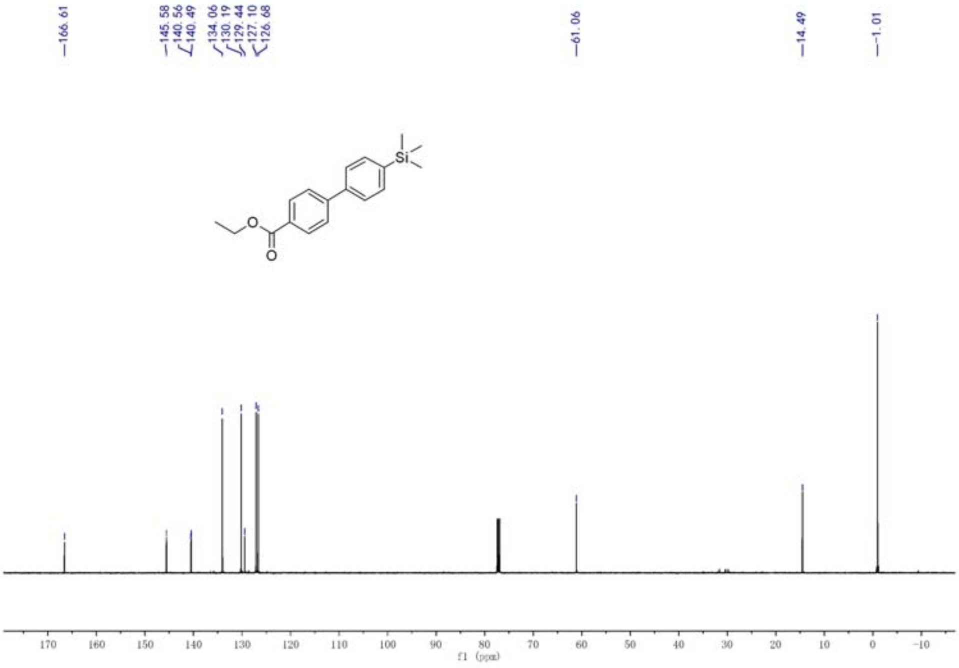

Supplementary Figure 50. ^{13}C NMR Spectrum of **3hi**

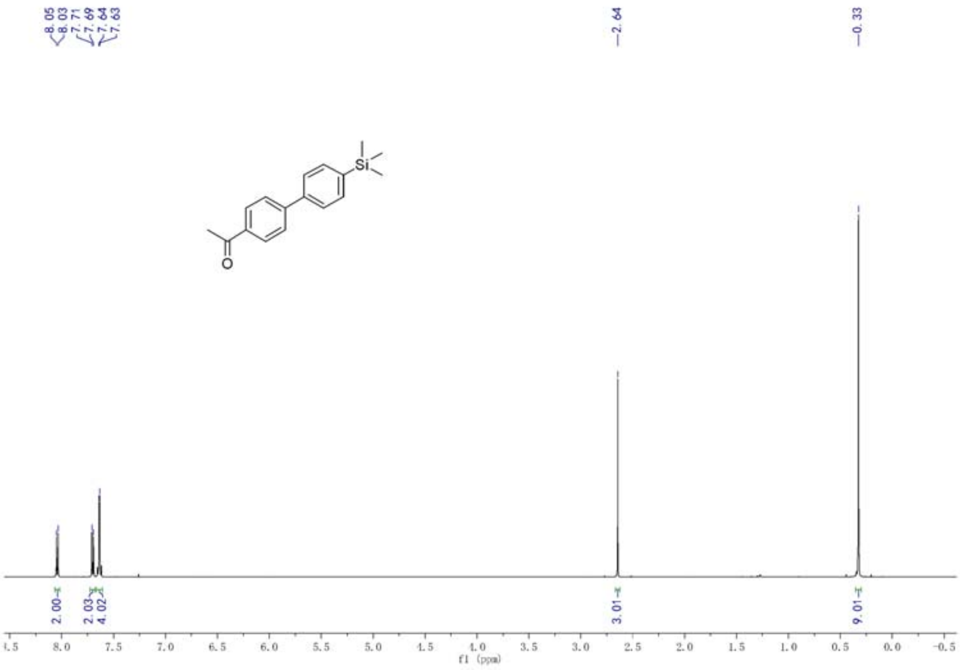

Supplementary Figure 51. ^{1}H NMR Spectrum of **3ii**

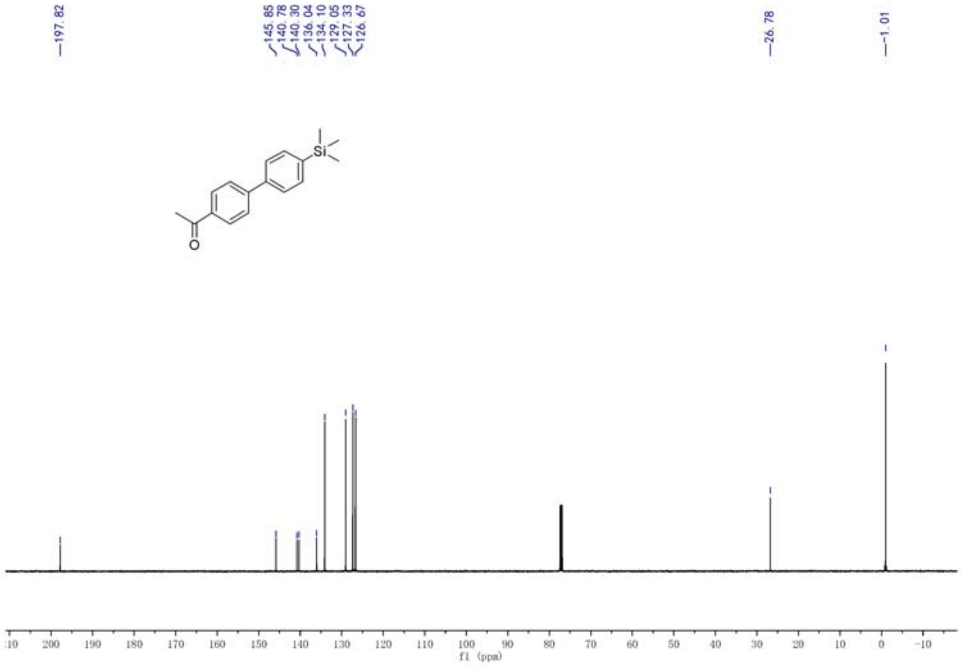

Supplementary Figure 52. ^{13}C NMR Spectrum of **3ii**

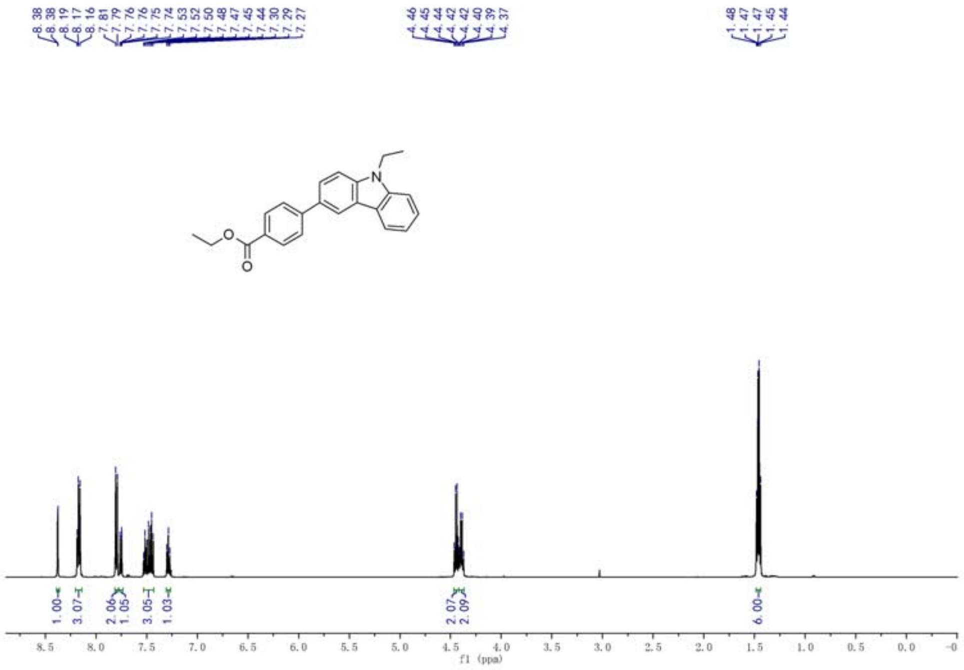

Supplementary Figure 53. ^{1}H NMR Spectrum of **3hj**

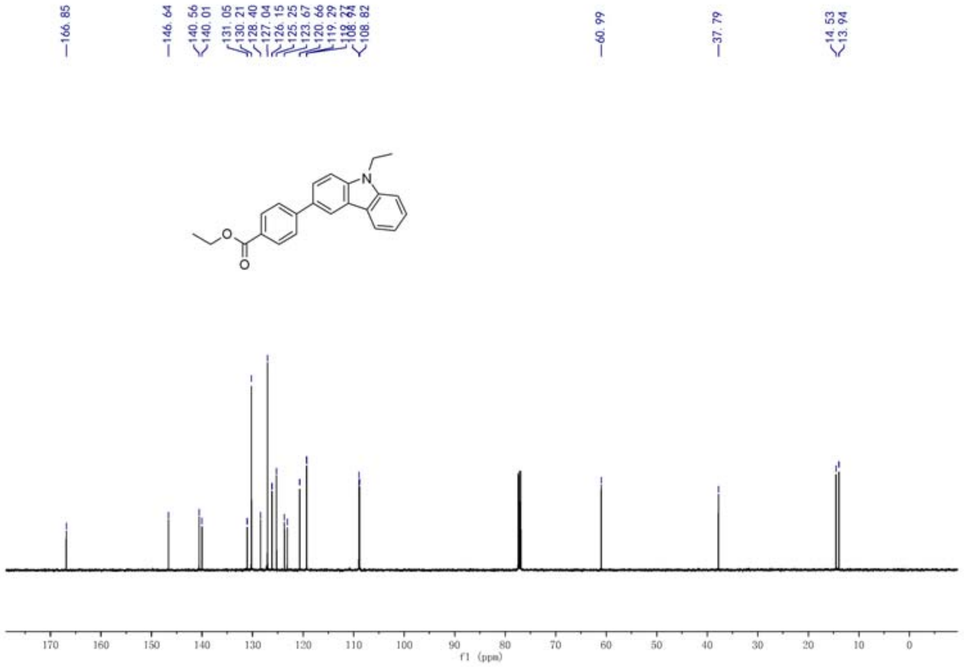

Supplementary Figure 54. ^{13}C NMR Spectrum of **3hj**

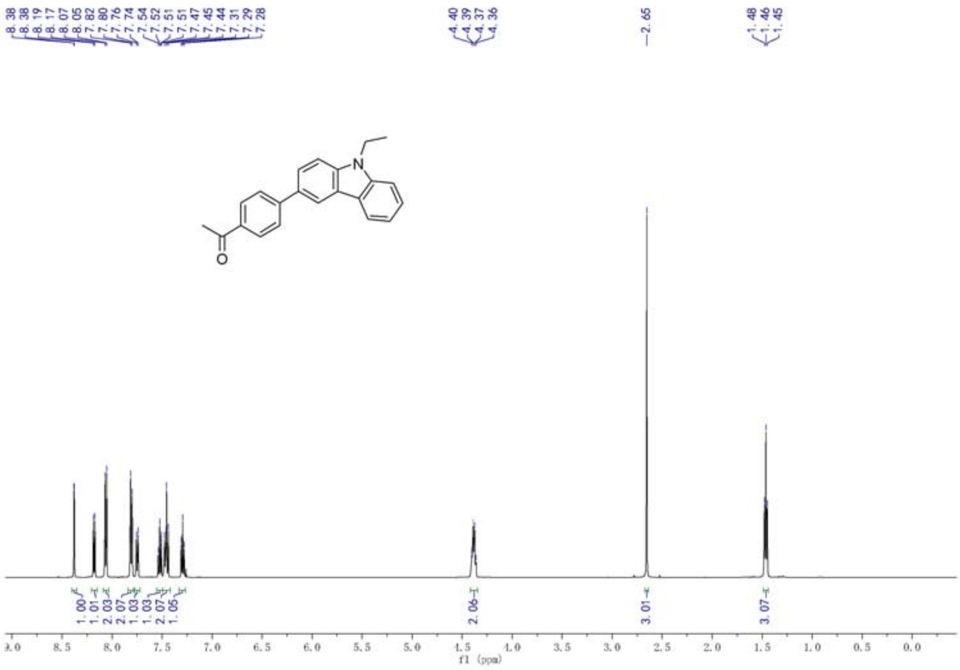

Supplementary Figure 55. ^{1}H NMR Spectrum of **3ij**

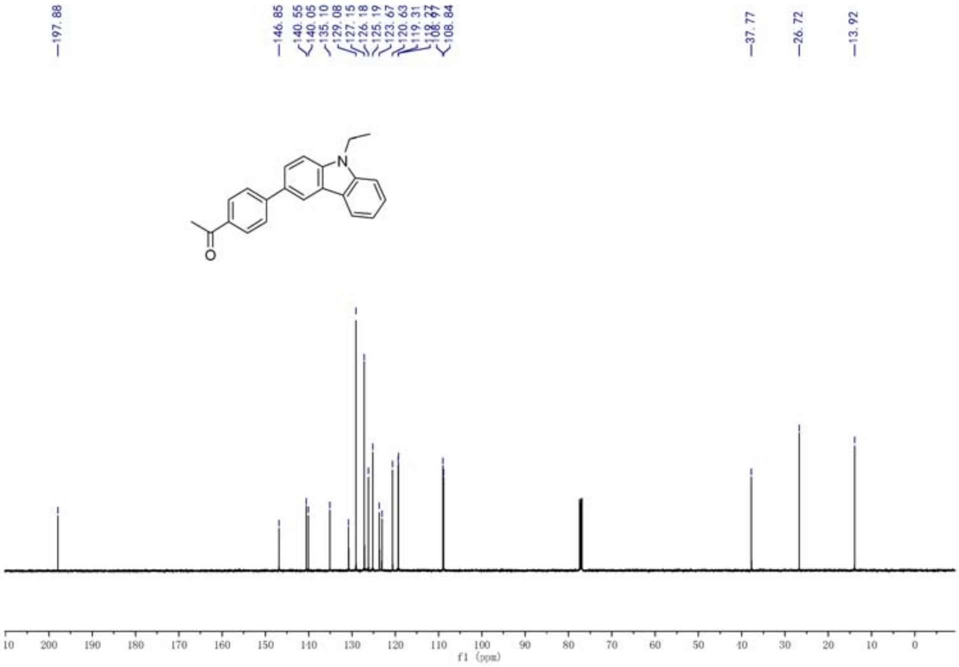

Supplementary Figure 56. ^{13}C NMR Spectrum of **3ij**

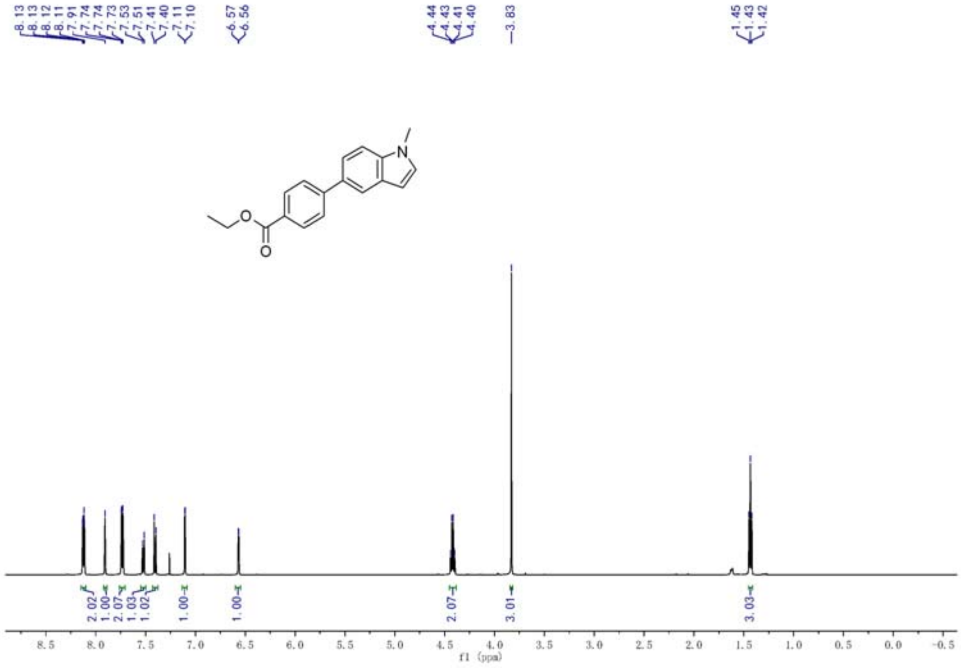

Supplementary Figure 57. ^{1}H NMR Spectrum of **3hk**

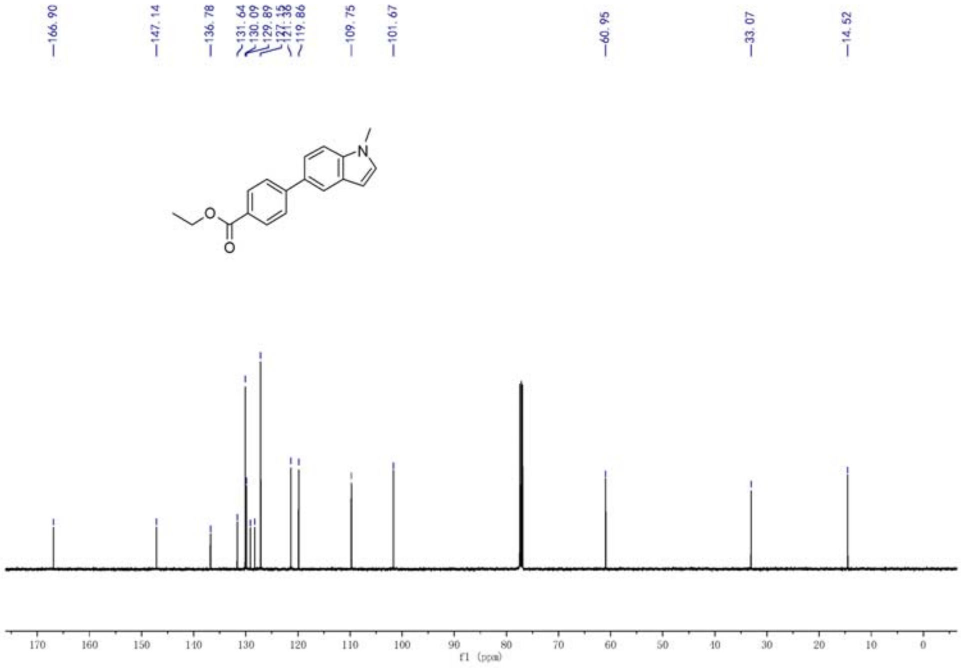

Supplementary Figure 58. ^{13}C NMR Spectrum of **3hk**

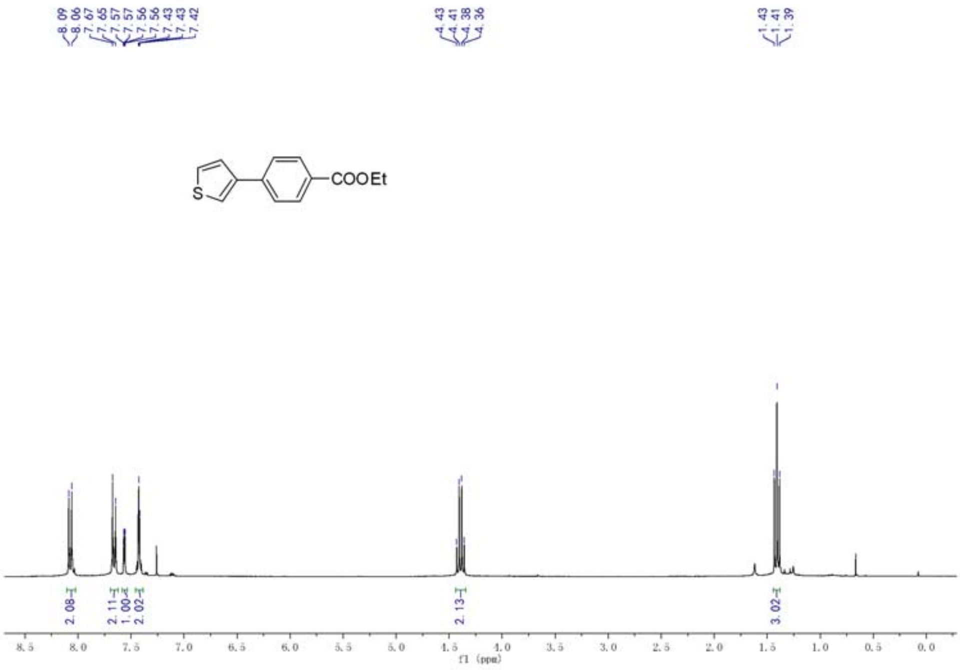

Supplementary Figure 59. ^{1}H NMR Spectrum of **3hl**

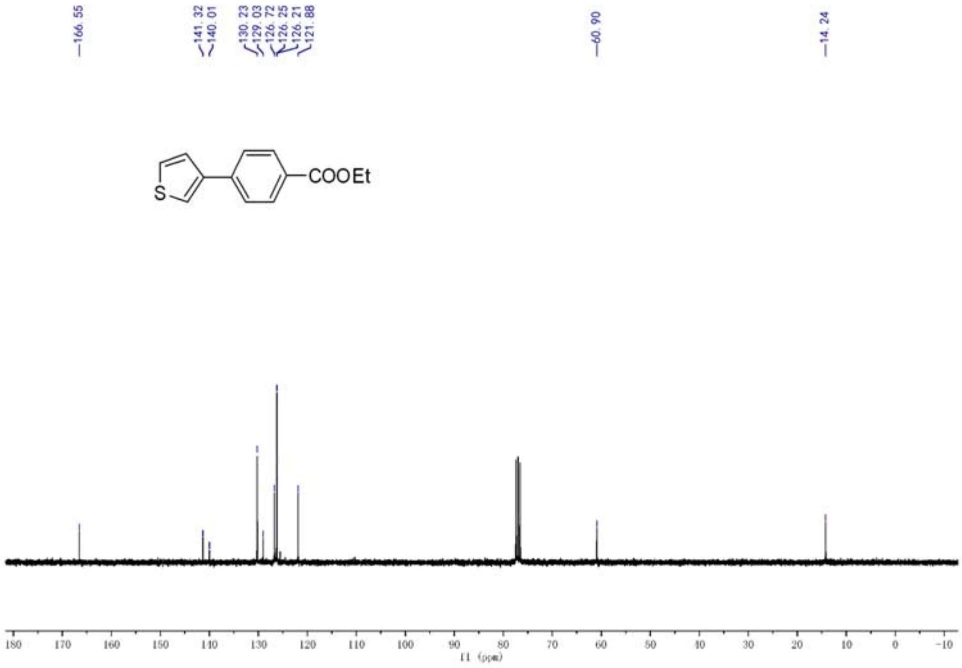

Supplementary Figure 60. ^{13}C NMR Spectrum of **3hl**

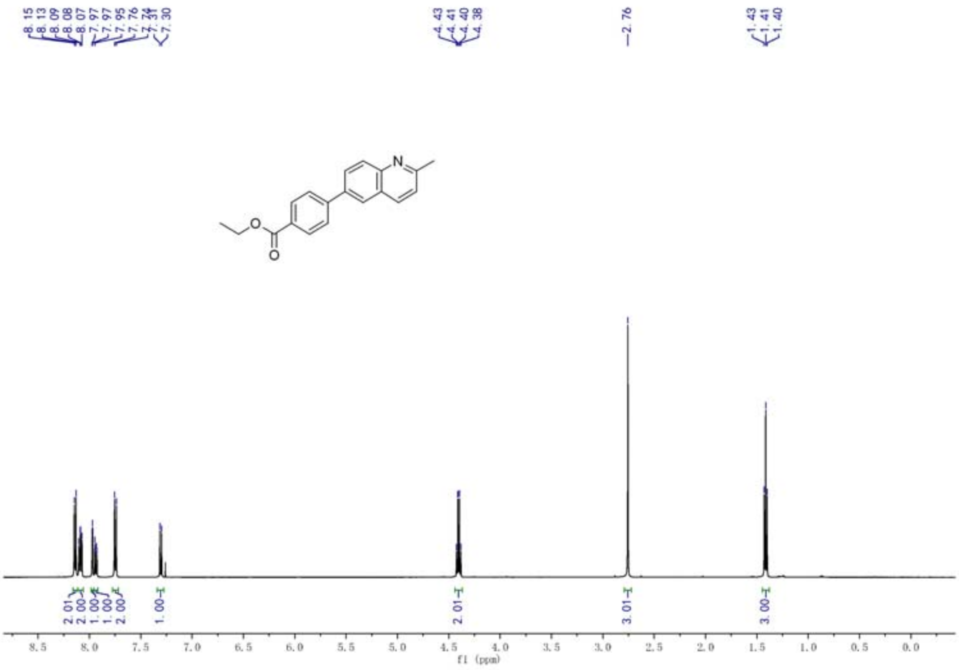

Supplementary Figure 61. ^{1}H NMR Spectrum of **3hm**

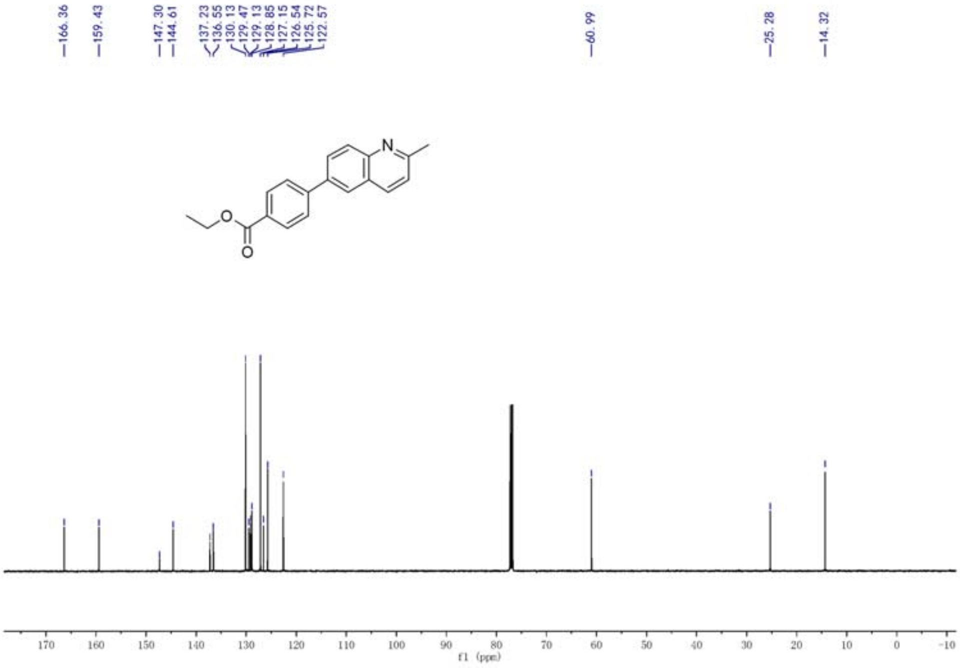

Supplementary Figure 62. ^{13}C NMR Spectrum of **3hm**

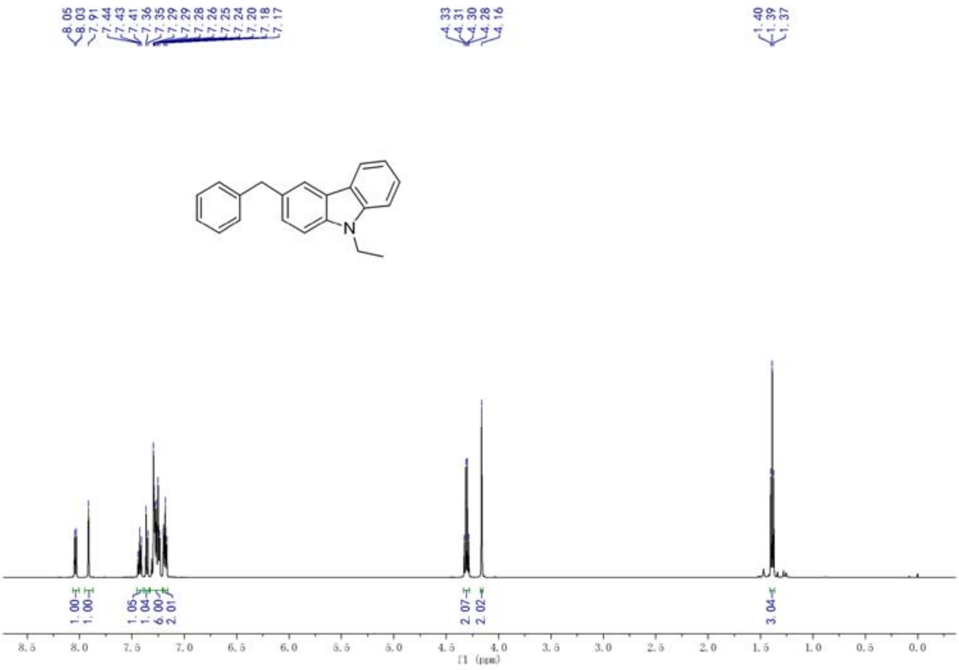

Supplementary Figure 63. ^{1}H NMR Spectrum of **5aj**

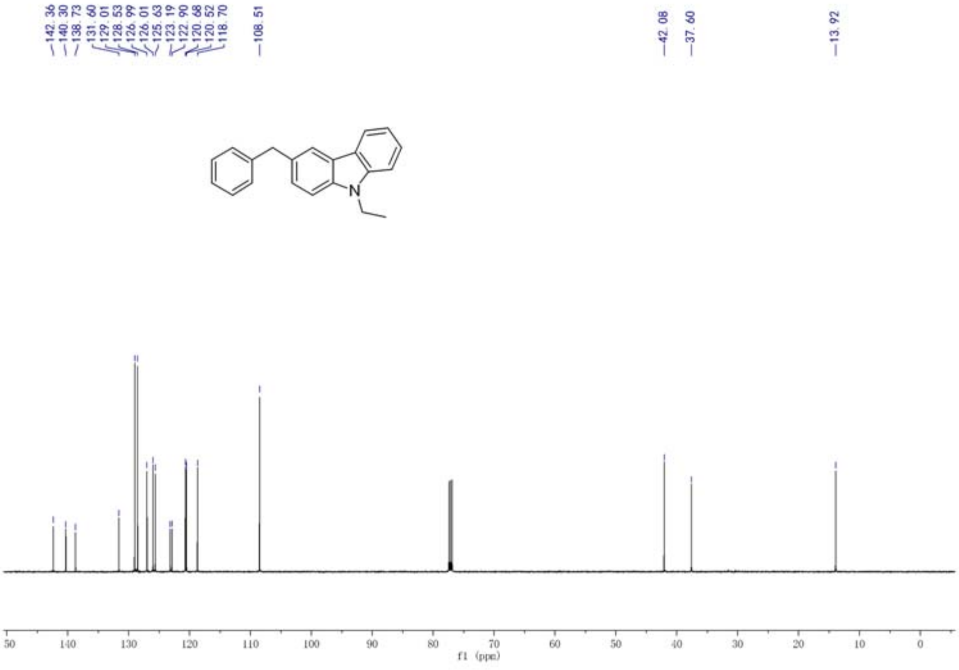

Supplementary Figure 64. ^{13}C NMR Spectrum of **5aj**

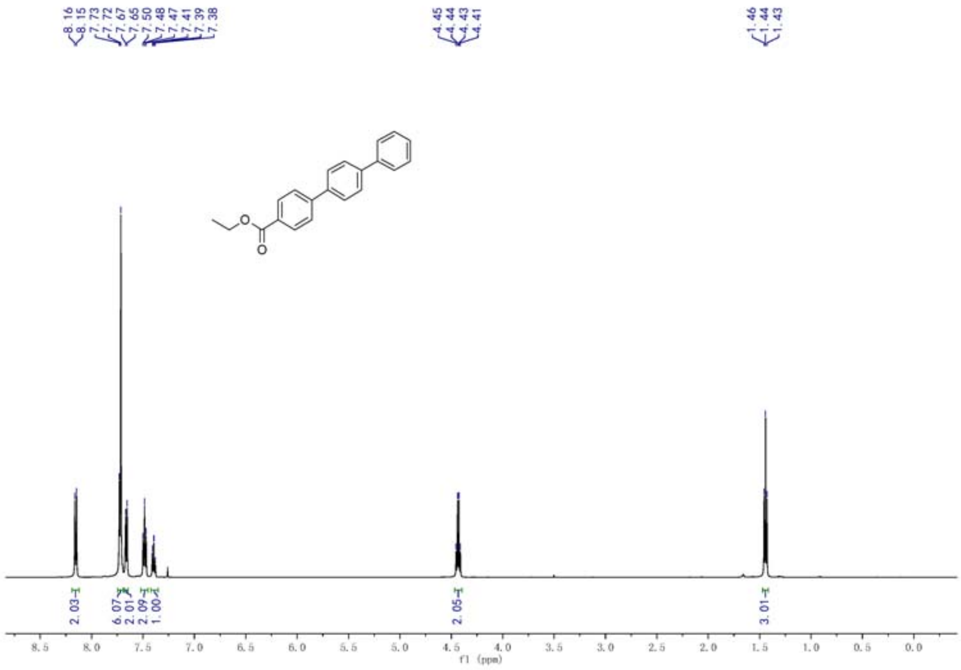

Supplementary Figure 65. ^{1}H NMR Spectrum of **3oa**

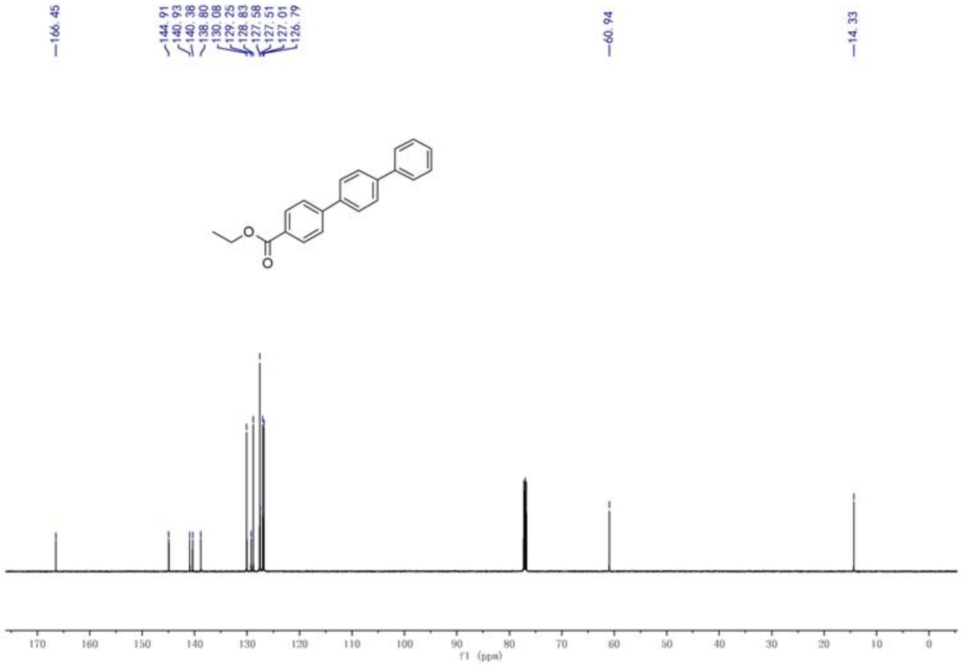

Supplementary Figure 66. ^{13}C NMR Spectrum of **3oa**

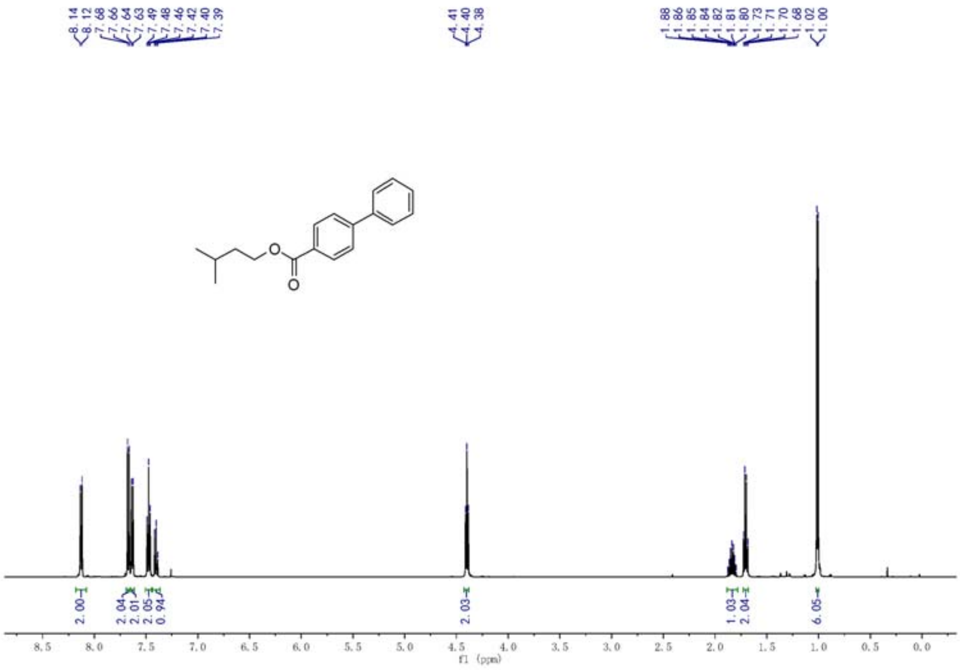

Supplementary Figure 67. ^{1}H NMR Spectrum of **3pa**

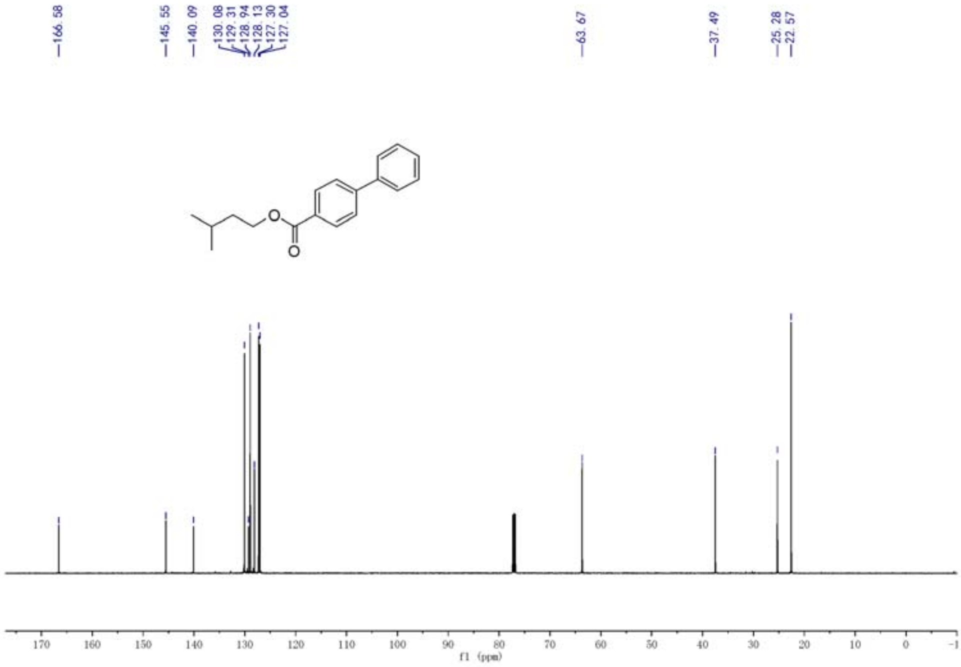

Supplementary Figure 68. ^{13}C NMR Spectrum of **3pa**

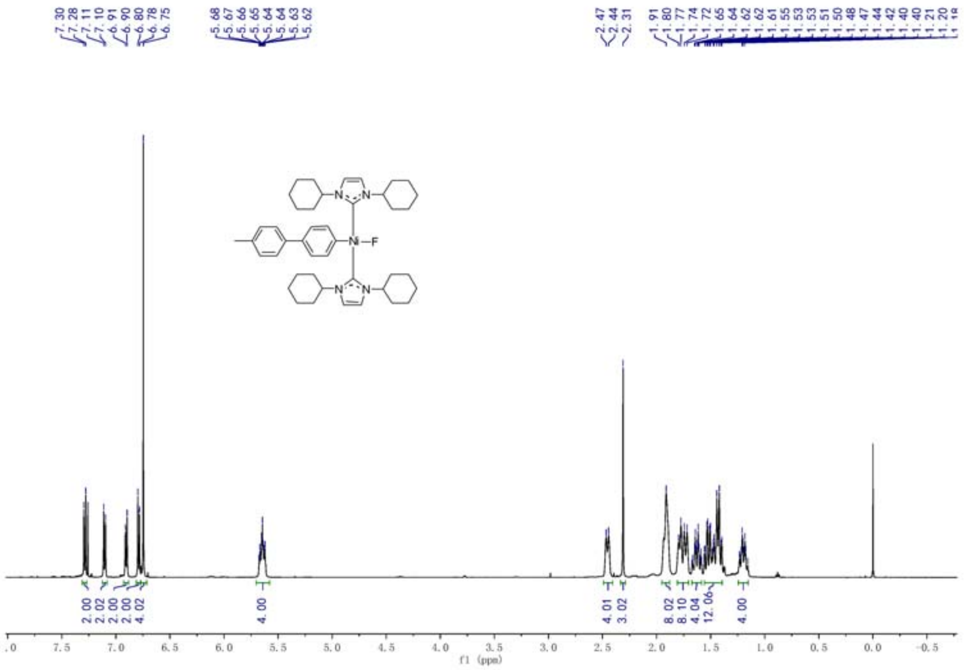

Supplementary Figure 69. ^{1}H NMR Spectrum of ***trans*-Ni(ICy)$_2$Ar$^{(1m)}$F**

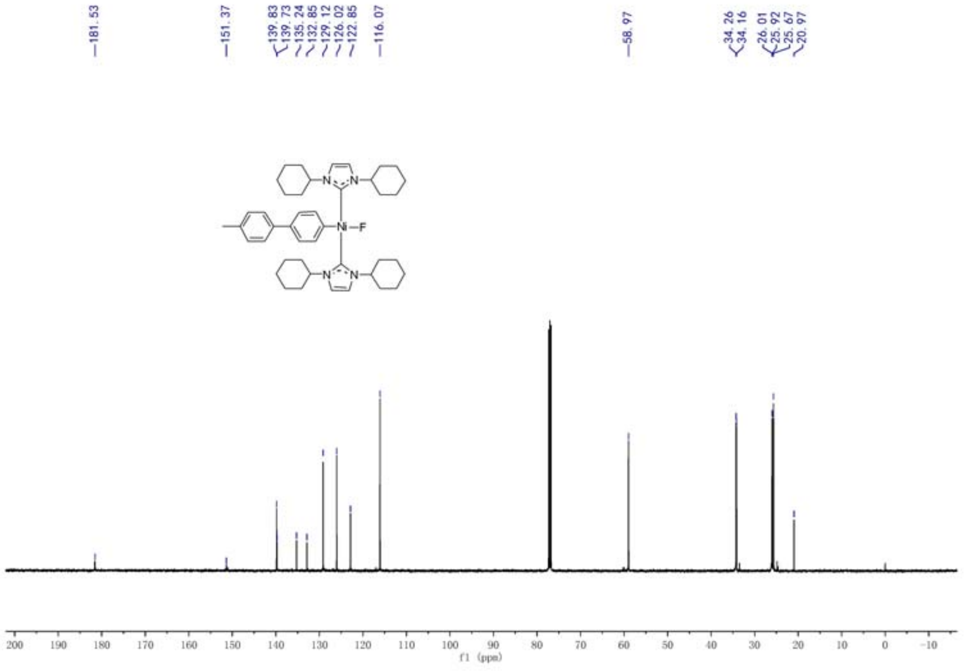

Supplementary Figure 70. ^{13}C NMR Spectrum of ***trans*-Ni(ICy)$_2$Ar$^{(1m)}$F**

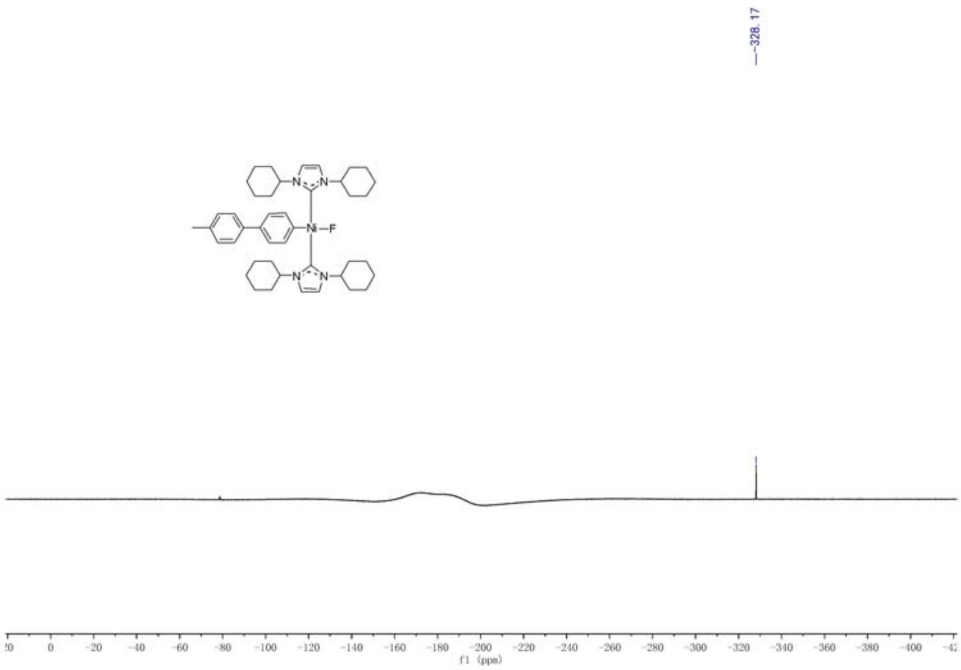

Supplementary Figure 71. ^{19}F NMR Spectrum of ***trans*-Ni(ICy)$_2$Ar$^{(1m)}$F**

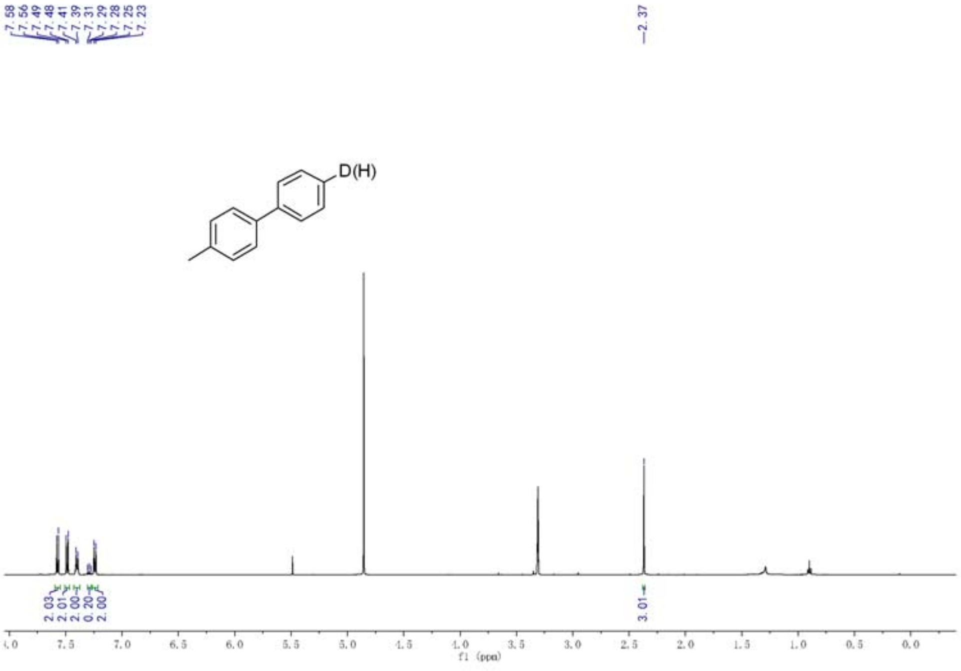

Supplementary Figure 72. ^{1}H NMR Spectrum of **4-methyl-1,1'-biphenyl-4'-*D***

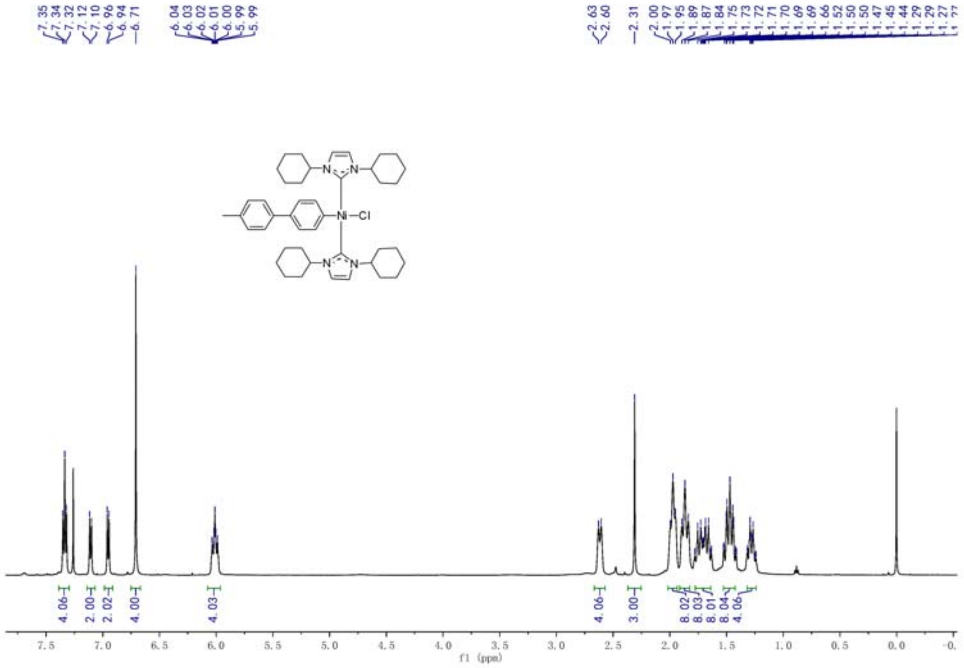

Supplementary Figure 73. ^{1}H NMR Spectrum of ***trans*-Ni(ICy)$_2$Ar$^{(1m)}$Cl**

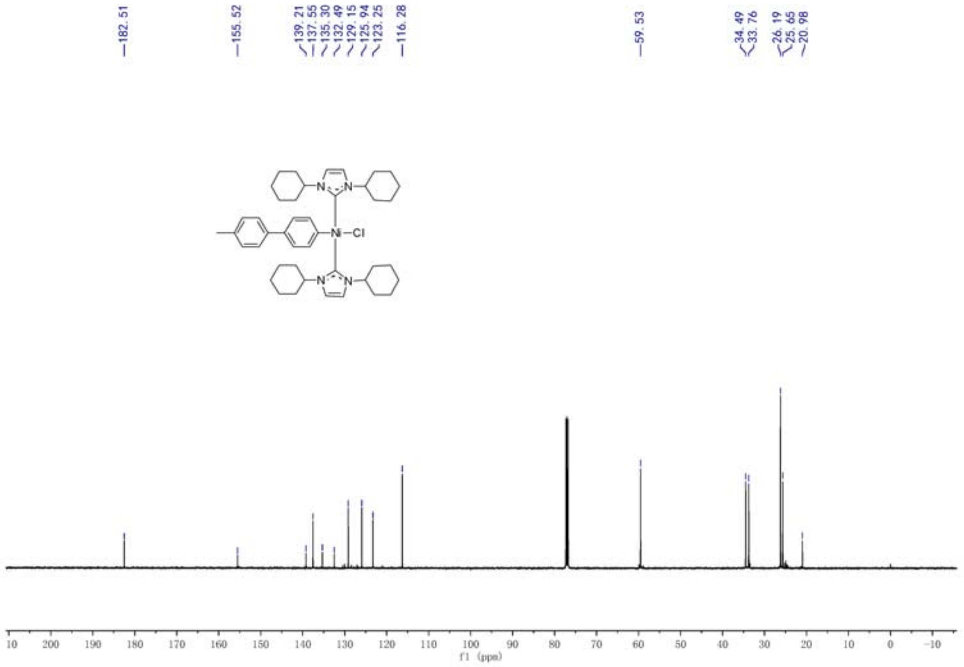

Supplementary Figure 74. ^{13}C NMR Spectrum of ***trans*-Ni(ICy)$_2$Ar$^{(1m)}$Cl**

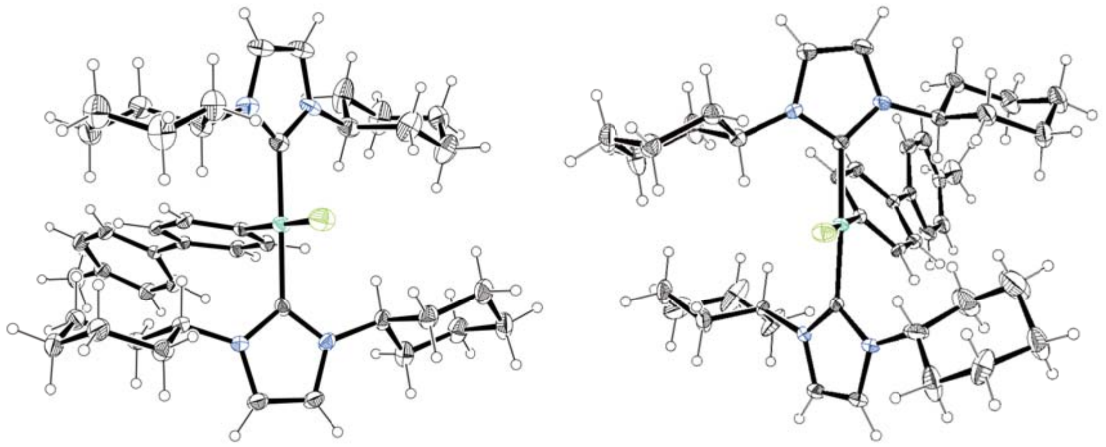

Supplementary Figure 75. Two Independent Molecules in the Crystal of ***trans*-Ni(ICy)$_2$Ar$^{(1m)}$F**.

Supplementary Table 1. Optimization of Reaction Conditions (1): Anions in Ammonium Salts and Ligands (internal standard for GC analysis: n-dodecane)

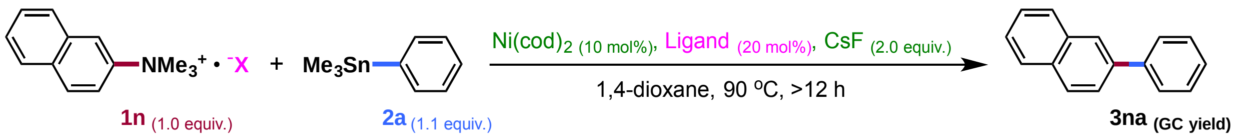

Entry	X	Ligand	GC Yield
1	I	ICy • HBF_4	trace
2	BF_4	ICy • HBF_4	35%
3	OTf	ICy • HBF_4	61%
4	OTf	IPr • HBF_4	48%
5	OTf	IBu • HBF_4	trace
6	OTf	IMes • HCl	trace
7	OTf	PCy_3 • HBF_4	–
8	OTf	DCyEPhos	trace
9	OTf	DCyPE	38%
10	OTf	XPhos	trace
11	OTf	TFP	trace
12	OTf	BINAP	–

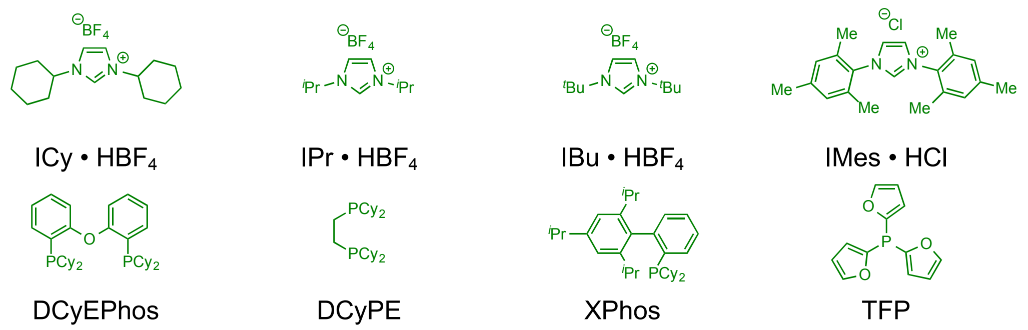

Supplementary Table 2. Optimization of Reaction Conditions (2): Bases (internal standard for GC analysis: n-dodecane)

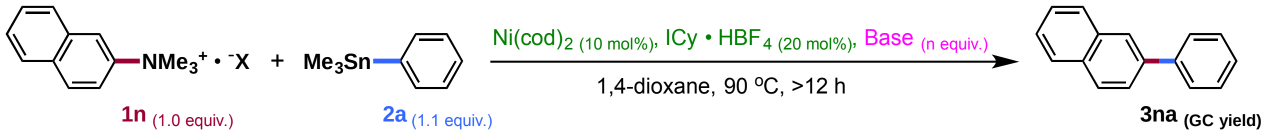

Entry	Base	n equiv.	GC Yield
1	NaO^{t}Bu	2.0	trace
2	CsF + CuCl (5 mol%)	2.0	-
3	K_3PO_4	2.0	trace
4	CsF + TBAF (5 mol%)	2.0	20%
5	KF	2.0	trace
6	CsF	2.0	61%
7	CsF	3.0	87%
8	CsF	4.0	78%

Supplementary Table 3. Optimization of Reaction Conditions (3): Solvents and Temperature (internal standard for GC analysis: n-dodecane)

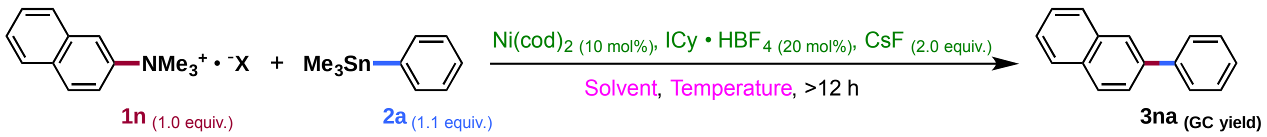

Entry	Temperature (°C)	Solvent	GC Yield
1	90	dioxane	87%
2	80	dioxane	91%
3	70	dioxane	17%
4	80	THF	64%
5	80	$Cl_2CH_2CH_2Cl_2$	-
6	80	DME	-
7	80	CH_3CN	-
8	80	Toluene	trace

Supplementary Table 4. Crystal Data and Structure Refinement for ***trans*-Ni(ICy)$_2$Ar$^{(1m)}$F** (CCDC: 1438708)

Identification code	v150826_kwh1_2	
Empirical formula	$C_{88.5}H_{124}F_2N_8Ni_2O_3$	
Formula weight	1503.37	
Temperature	100(2) K	
Wavelength	1.54178 Å	
Crystal system	Triclinic	
Space group	*P*-1	
Unit cell dimensions	*a* = 13.4293(8) Å	α = 88.045(2)°.
	b = 13.6016(8) Å	β = 82.252(2)°.
	c = 25.1405(14) Å	γ = 66.250(2)°.
Volume	4163.9(4) Å^3	
Z	2	
Density (calculated)	1.199 Mg/m^3	
Absorption coefficient	1.001 mm^{-1}	
F(000)	1618	
Crystal size	0.120 x 0.120 x 0.080 mm^3	
Theta range for data collection	3.926 to 71.880°.	
Index ranges	-15<=*h*<=15, -16<=*k*<=16, -30<=*l*<=29	
Reflections collected	35571	
Independent reflections	14377 [$R_{(int)}$ = 0.0474]	
Completeness to theta = 67.679°	91.7 %	
Absorption correction	Empirical	
Max. and min. transmission	0.9243 and 0.8354	
Refinement method	Full-matrix least-squares on F^2	
Data / restraints / parameters	14377 / 3 / 959	
Goodness-of-fit on F_2	1.087	
Final *R* indices [*I*>2sigma(*I*)]	R_1 = 0.0752, wR_2 = 0.2079	
R indices (all data)	R_1 = 0.0925, wR_2 = 0.2501	
Extinction coefficient	n/a	
Largest diff. peak and hole 1.804 and -1.466 e.Å^{-3}		

Supplementary Table 5. Crystal Data and Structure Refinement for ***trans*-Ni(ICy)$_2$Ar$^{(1m)}$Cl**. (CCDC: 1438709)

Identification code	v151118_kwh1_4	
Empirical formula	$C_{44}H_{60}Cl_4N_4Ni$	
Formula weight	845.47	
Temperature	100(2) K	
Wavelength	1.54178 Å	
Crystal system	Monoclinic	
Space group	$P2_1/c$	
Unit cell dimensions	a = 12.3333(9) Å	α = 90°.
	b = 11.1794(8) Å	β = 100.673(3)°.
	c = 32.972(3) Å	γ = 90°.
Volume	4467.5(6) Å^3	
Z	4	
Density (calculated)	1.257 Mg/m^3	
Absorption coefficient	3.081 mm^{-1}	
F(000)	1792	
Crystal size	0.130 x 0.100 x 0.080 mm^3	
Theta range for data collection	2.727 to 72.661°.	
Index ranges	-14<=h<=15, -13<=k<=13, -40<=l<=40	
Reflections collected	46220	
Independent reflections	8116 [$R_{(int)}$ = 0.0704]	
Completeness to theta = 67.679°	92.3 %	
Absorption correction	Empirical	
Max. and min. transmission	0.7963 and 0.6882	
Refinement method	Full-matrix least-squares on F^2	
Data / restraints / parameters	8116 / 0 / 479	
Goodness-of-fit on F^2	1.032	
Final R indices [I>2sigma(I)]	R_1 = 0.0841, wR_2 = 0.2152	
R indices (all data)	R_1 = 0.0931, wR_2 = 0.2225	
Extinction coefficient	n/a	
Largest diff. peak and hole	2.973 and -0.929 e.Å^{-3}	

Supplementary Discussion

Additional Discussions/Explanations for the Transmetalation Step from CP2-1 to CP2-2 (Supplementary Figure 1): After the formation of *cis*-$NHC_2Ni(II)ArF$ (**CP2-1**), one ICy ligand opposite the Ph group would leave to form **INT-a**. An in-depth scan of the potential energy surface showed that the energy change for the release of ICy is a simple uphill process (no TS). The C–F bond in **INT-a** then rotates from the *cis*- to the *trans*-position to Ph (**INT-b**), *via* a very low energy barrier (**TS-a_b**). PhSnMe3 then approaches **INT-b** to form **CP2-2**, leading to a thermodynamically very stable coupling product. In the absence of $PhSnMe_3$, as indicated by the control experiments in **Figure 3** and **Table 2**, disassociated ICy would again coordinate to form *trans*-**RS**, which is ca. 10 kcal/mol more stable than **CP2-1**. Hence, the total activation barrier of the transmetalation from **RS** to **CP2-2** adds up to over 30 kcal/mol, which would be kinetically difficult under the current reaction conditions, as reflected in the very low yield in the control experiments (**Figure 3**). A similar process for Pd-catalysis has been reported in the literature (ref. 41-48).

Additional Discussions for Ni(I)-mediated Mechanism (Supplementary Figure 2, 3, and 4): We have performed DFT calculation for the Ni(I)/Ni(III) mechanism at the same level as the Ni(0)/Ni(II) route (B3LYP&M06). The results are summarized in **Supplementary Figure 2 and 3**. Based on reported information, we envisioned that the Ni(I) catalyst $Ni^{I}(ICy)_2F$ would be firstly generated from $Ni^{0}(ICy)_2$ and $[PhNMe_3]^{+}F^{-}$, similar to the reported reaction between $Ni^{0}(ICy)_2$ and ArX (X = Cl, Br, *etc*.). The resultant $Ni^{I}(ICy)_2F$ reacts with $PhSnMe_3$ to form **CP-a**, $Ni^{I}(ICy)_2Ph$, *via* Ni/Sn transmetalation with large endothermicity (+11.3 kcal/mol). Then, the Ni(I)–□ complex **CP-b** is formed with a reasonable activation energy (+24.6 kcal mol^{-1}), albeit again with +13.3 kcal mol^{-1} endothermicity. From **CP-b**, C–N bond cleavage takes place through **TS-b** with an energy loss of +6.0 kcal mol^{-1}. IRC analysis for **TS-b** failed to locate the proposed Ni(III) intermediate, and instead, a straightforward C–C bond formation occurs, leading directly to the final product.

In summary, the theoretical calculations indicate that, as shown in **Supplementary Figure 3**, the CPs and TSs in the Ni(0)/Ni(II) route (green line) are energetically more favorable than those in the Ni(I)/Ni(III) pathway (pink line), and the reaction is therefore much less likely to take place along the Ni(I)/Ni(III) route than along the Ni(0)/Ni(II) route.

It is also important to note that in the stoichiometric reaction between Ni(0) catalyst and ammonium salts (**Table 2** and **Figure 3**), no Ni(I) species was detected at all.

Finally, we have done several control experiments. When we used the Ni(I) catalyst (in-situ synthesized as reported in the literature) instead of the Ni(0) catalyst, the reaction became very sluggish, as shown in **Supplementary Figure 4**. Hence, although the possibility of the Ni(I)/Ni(III) mechanism cannot be totally ruled out, all the current computational and experimental results support the view that the Ni(0)/Ni(II) route is more favorable and would be at least the predominant reaction pathway.

Supplementary Methods

General procedure for preparation of aryltrimethylammonium triflates.[1] To a stirred solution of *N*,*N*–dimethylarylamine (10 mmol) in CH_2Cl_2 (10 mL) was added dropwise methyl trifluoromethanesulfonate (1.24 mL, 11.0 mmol, 1.1 equiv.) at room temperature. The resulting solution was stirred for 4 h or overnight at room temperature. Solvent was removed in vacuo and the residue was washed with Et_2O, dried under vacuum to give a white solid.

General procedure for cross-coupling between aryltrimethylammonium triflates 1 and of $ArSnMe_3$ 2 (see Table 1 and Figure 2a and 2c). A Schlenk tube was charged aryltrimethylammonium triflates (0.5 mmol), $Ni(cod)_2$ (13.8 mg, 0.05 mmol), ICy•HBF_4 (32.0 mg, 0.1 mmol), CsF (227.9 mg, 1.5 mmol), $ArSnMe_3$ (0.55 mmol) and dioxane (5 mL) under a argon atmosphere. The reaction mixture was stirred at 80 °C overnight and then cooled to room temperature. Water (10 mL) was added and the resulting mixture was extracted with ethyl acetate (3 x 10 mL). The combined organic layer was dried over Na_2SO_4, filtered and concentrated. The residue was purified by column chromatography on sillca gel.

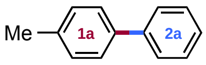

3aa

Colorless oil, isolated yield 60%; ^{1}H NMR (300 MHz, $CDCl_3$) δ 7.72–7.67 (m, 2H), 7.63–7.58 (m, 2H), 7.56–7.50 (m, 2H), 7.48–7.34 (m, 3H), 2.50 (s, 3H). ^{13}C NMR (75 MHz, $CDCl_3$) δ 141.25, 138.45, 137.11, 129.55, 128.82, 128.78, 127.32, 127.24, 127.07, 127.04, 21.00. All spectral data match those previously reported.[2]

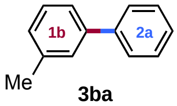

3ba

Colorless oil, isolated yield 71%; ^{1}H NMR (300 MHz, $CDCl_3$) δ 7.64–7.60 (m, 2H), 7.49–7.34 (m, 6H), 7.24–7.15 (m, 1H), 2.45 (s, 3H). ^{13}C NMR (75 MHz, $CDCl_3$) δ 141.45, 141.33, 138.41, 128.82, 128.76, 128.73, 128.06, 128.05, 127.31, 127.25, 127.23, 124.33, 21.45. All spectral data match those previously reported.[3]

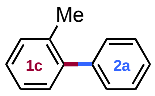

3ca

Colorless oil, isolated yield 83%; ^{1}H NMR (300 MHz, $CDCl_3$) δ 7.44–7.37 (m, 2H), 7.36–7.30 (m, 3H), 7.28–7.21 (m, 4H), 2.27 (s, 3H). ^{13}C NMR (75 MHz, $CDCl_3$) δ 142.04, 135.41, 130.36, 129.85, 129.25, 128.81, 128.11, 127.30, 126.81, 125.81, 20.36. All spectral data match those previously reported.[3]

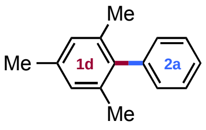

3da

Colorless oil, isolated yield 35%; ^{1}H NMR (500 MHz, $CDCl_3$) δ 7.42–7.34 (m, 2H), 7.32–7.25 (m, 1H), 7.16–7.07 (m, 2H), 6.92 (d, J = 9.2 Hz, 2H), 2.31 (d, J = 9.5 Hz, 3H), 1.98 (d, J = 10.5 Hz, 6H). ^{13}C NMR (125 MHz, $CDCl_3$) δ 141.24, 139.19, 136.64, 136.06, 129.42, 128.49, 128.18, 126.62, 21.15, 20.86. All spectral data match those previously reported.[4]

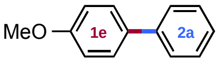

3ea

Colorless solid, isolated yield 40%; ^{1}H NMR (500 MHz, $CDCl_3$) δ 7.63–7.56 (m, 4H), 7.47 (t, *J* = 7.7 Hz, 2H), 7.35 (t, *J* = 7.4 Hz, 1H), 7.03 (d, *J* = 8.8 Hz, 2H), 3.88 (s, 3H). ^{13}C NMR (125 MHz, $CDCl_3$) δ 159.26, 140.92, 133.86, 128.83, 128.25, 126.83, 126.76, 114.31, 55.41. All spectral data match those previously reported.[2]

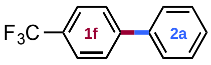

3fa

Colorless solid, isolated yield 91%; ^{1}H NMR (300 MHz, $CDCl_3$) δ 7.71 (s, 4H), 7.64–7.59 (m, 2H), 7.54–7.38 (m, 3H). ^{13}C NMR (75 MHz, $CDCl_3$) δ 144.99, 140.02, 129.21, 128.41, 127.65, 127.51, 125.90, 125.85. All spectral data match those previously reported.[2]

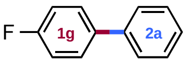

3ga

Colorless solid, isolated yield 81%; ^{1}H NMR (300 MHz, $CDCl_3$) δ 7.68–7.52 (m, 4H), 7.51–7.32 (m, 3H), 7.20–7.07 (m, 2H). ^{13}C NMR (75 MHz, $CDCl_3$) δ 162.6 (d, *J* = 244.5), 141.33, 140.35, 137.45, 128.89, 128.83, 128.70, 127.32, 127.24, 127.09, 115.66 (d, *J* = 21.0). All spectral data match those previously reported.[5]

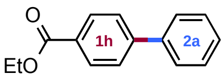

3ha

Colorless solid, isolated yield 95%; ^{1}H NMR (400 MHz, $CDCl_3$) δ 8.13 (d, *J* = 8.4 Hz, 2H), 7.65 (dd, *J* = 13.9, 7.8 Hz, 4H), 7.47 (t, *J* = 7.6 Hz, 2H), 7.40 (t, *J* = 7.3 Hz, 1H), 4.41 (q, *J* = 7.1 Hz, 2H), 1.42 (t, *J* = 7.1 Hz, 3H). ^{13}C NMR (75 MHz, $CDCl_3$) δ 166.62, 145.59, 140.10, 130.10, 129.28, 128.95, 128.14, 127.31, 127.04, 60.90, 14.22. All spectral data match those previously reported.[6]

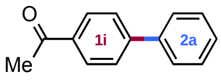

3ia

Colorless solid, isolated yield 93%; ^{1}H NMR (300 MHz, $CDCl_3$) δ 8.04 (d, *J* = 8.5 Hz, 2H), 7.72–7.60 (m, 4H), 7.44 (dt, *J* = 21.9, 7.2 Hz, 3H), 2.64 (s, 3H). ^{13}C NMR (75 MHz, $CDCl_3$) δ 198.07, 145.98, 140.05, 136.02, 129.14, 129.10, 128.42, 127.44, 127.40, 26.69. All spectral data match those previously reported.[7]

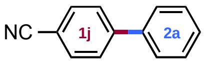

3ja

Colorless solid, isolated yield 52%; ^{1}H NMR (300 MHz, $CDCl_3$) δ 7.71 (q, *J* = 8.6 Hz, 4H), 7.59 (d, *J* = 6.7 Hz, 2H), 7.53–7.38 (m, 3H). ^{13}C NMR (75 MHz, $CDCl_3$) δ 145.75, 139.24, 132.65, 129.16, 128.71, 127.78, 127.27, 118.99, 110.91. All spectral data match those previously reported.[8]

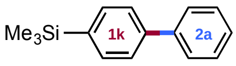

3ka

Colorless solid, isolated yield 78%; ^{1}H NMR (500 MHz, $CDCl_3$) δ 7.65–7.60 (m, 6H), 7.46 (t, *J* = 7.6 Hz, 2H), 7.37 (t, *J* = 7.4 Hz, 1H), 0.33 (s, 9H). ^{13}C NMR (125 MHz, $CDCl_3$) δ 141.59, 141.17, 139.20, 133.80, 128.74, 127.30, 127.15, 126.49, –1.10. All spectral data match those previously reported.[9]

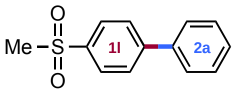

3la

Colorless solid, isolated yield 92%; ^{1}H NMR (500 MHz, $CDCl_3$) δ 8.01 (d, *J* = 8.6 Hz, 2H), 7.77 (d, *J* = 8.6 Hz, 2H), 7.61 (d, *J* = 7.0 Hz, 2H), 7.49 (t, *J* = 7.4 Hz, 2H), 7.44 (d, *J* = 7.3 Hz, 1H), 3.10 (s, 3H). ^{13}C NMR (125 MHz, $CDCl_3$) δ 146.73, 139.14, 129.13, 128.71, 128.01, 127.93, 127.41, 44.64. All spectral data match those previously reported.[8]

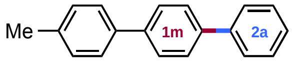

3ma

Colorless solid, isolated yield 92%; ^{1}H NMR (300 MHz, $CDCl_3$) δ 7.68 (dd, *J* = 10.8, 2.3 Hz, 6H), 7.58 (d, *J* = 8.2 Hz, 2H), 7.53–7.46 (m, 2H), 7.40 (d, *J* = 7.1 Hz, 1H), 7.35–7.27 (m, 2H), 2.44 (s, 3H). ^{13}C NMR (75 MHz, $CDCl_3$) δ 141.02, 140.30, 140.07, 138.05, 137.39, 129.77, 129.02, 127.68, 127.51, 127.25, 127.10, 21.18. All spectral data match those previously reported.[9]

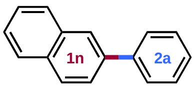

3na

Colorless solid, isolated yield 91%; ^{1}H NMR (500 MHz, $CDCl_3$) δ 8.01 (s, 1H), 7.90–7.79 (m, 3H), 7.74–7.65 (m, 3H), 7.52–7.39 (m, 4H), 7.37–7.30 (m, 1H). ^{13}C NMR (126 MHz, $CDCl_3$) δ 141.24, 138.67, 133.80, 132.74, 128.97, 128.54, 128.32, 127.76, 127.55, 127.46, 126.40, 126.04, 125.92, 125.71. All spectral data match those previously reported.[9]

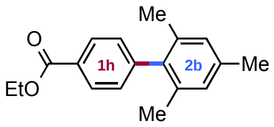

3hb

Colorless solid, isolated yield 55%; ^{1}H NMR (300 MHz, $CDCl_3$) δ 8.11 (d, *J* = 8.4 Hz, 2H), 7.23 (d, *J* = 8.5 Hz, 2H), 6.95 (d, *J* = 0.5 Hz, 2H), 4.41 (q, *J* = 7.2 Hz, 2H), 2.33 (s, 3H), 1.98 (s, 6H), 1.42 (t, *J* = 7.1 Hz, 3H). ^{13}C NMR (75 MHz, $CDCl_3$) δ 166.80, 146.27, 138.13, 137.14, 135.61, 129.78, 129.52, 128.92, 128.24, 60.90, 20.92, 20.52, 14.26. All spectral data match those previously reported.[4]

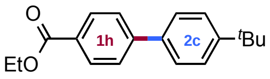

3hc

Colorless solid, isolated yield 91%; ^{1}H NMR (500 MHz, $CDCl_3$) δ 8.12 (d, *J* = 8.3 Hz, 2H), 7.67 (d, *J* = 8.3 Hz, 2H), 7.59 (d, *J* = 8.4 Hz, 2H), 7.51 (d, *J* = 8.4 Hz, 2H), 4.42 (q, *J* = 7.1 Hz, 2H), 1.43 (t, *J* = 7.1 Hz, 3H), 1.38 (s, 9H). ^{13}C NMR (126 MHz, $CDCl_3$) δ 166.59, 151.31, 145.38, 137.13, 130.06, 129.01, 126.95, 126.81, 125.92, 60.93, 34.65, 31.34, 14.40. HRMS (pos. ESI) *m/z*: calcd for $C_{19}H_{22}O_2Na$ $[M+Na]^+$ 305.1518, found 305.1514.

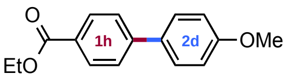

3hd

Colorless solid, isolated yield 83%; ^{1}H NMR (300 MHz, $CDCl_3$) δ 8.09 (d, *J* = 8.2 Hz, 2H), 7.60 (dd, *J* = 12.8, 8.6 Hz, 4H), 7.00 (d, *J* = 8.7 Hz, 2H), 4.40 (q, *J* = 7.2 Hz, 2H), 3.86 (s, 3H), 1.41 (t, *J* = 7.1 Hz, 3H). ^{13}C NMR (75 MHz, $CDCl_3$) δ 166.72, 159.92, 145.19, 132.53, 130.12, 128.65, 128.42, 126.48, 114.39, 60.86, 55.32, 14.25. All spectral data match those previously reported.[6]

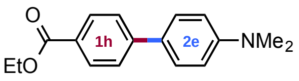

3he

Colorless solid, isolated yield 92%; ^{1}H NMR (300 MHz, $CDCl_3$) δ 8.07 (d, *J* = 8.6 Hz, 2H), 7.59 (dd, *J* = 18.6, 8.7 Hz, 4H), 6.81 (d, *J* = 8.6 Hz, 2H), 4.39 (q, *J* = 7.2 Hz, 2H), 3.01 (s, 6H), 1.41 (t, *J* = 7.1 Hz, 3H). ^{13}C NMR (75 MHz, $CDCl_3$) δ 166.85, 145.58, 130.09, 127.95, 125.79, 112.64, 60.73, 40.36, 14.27. All spectral data match those previously reported.[10]

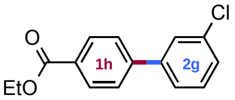

3hg

Colorless solid, isolated yield 72%; ^{1}H NMR (500 MHz, $CDCl_3$) δ 8.12 (d, *J* = 8.3 Hz, 2H), 7.65–7.60 (m, 3H), 7.49 (d, *J* = 7.3 Hz, 1H), 7.38 (dt, *J* = 8.0, 4.8 Hz, 2H), 4.41 (q, *J* = 7.1 Hz, 2H), 1.42 (t, *J* = 7.1 Hz, 3H). ^{13}C NMR (126 MHz, $CDCl_3$) δ 166.33, 144.02, 141.90, 134.86, 130.15, 130.14, 129.85, 128.08, 127.40, 127.00, 125.43, 61.06, 14.34. All spectral data match those previously reported.[11]

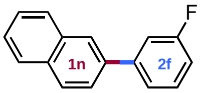

3nf

Colorless solid, isolated yield 88%; ^{1}H NMR (500 MHz, $CDCl_3$) δ 8.06 (d, *J* = 0.7 Hz, 1H), 7.97–7.89 (m, 3H), 7.74 (dd, *J* = 8.5, 1.8 Hz, 1H), 7.57–7.51 (m, 3H), 7.50–7.43 (m, 2H), 7.15–7.07 (m, 1H). ^{13}C NMR (126 MHz, $CDCl_3$) δ 164.39, 162.43, 143.57, 143.51, 137.37, 137.35, 133.71, 132.99, 130.45, 130.39, 128.74, 128.39, 127.80, 126.59, 126.38, 126.11, 125.35, 123.15, 123.13, 114.47, 114.34, 114.29, 114.17. All spectral data match those previously reported.[12]

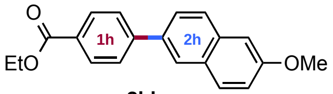

3hh

Colorless solid, isolated yield 90%; ^{1}H NMR (500 MHz, $CDCl_3$) δ 8.15 (d, *J* = 8.5 Hz, 2H), 8.01 (d, *J* = 1.5 Hz, 1H), 7.81 (t, *J* = 7.9 Hz, 2H), 7.76 (d, *J* = 8.5 Hz, 2H), 7.72 (dd, *J* = 8.5, 1.8 Hz, 1H), 7.20 (dd, *J* = 8.9, 2.5 Hz, 1H), 7.16 (d, *J* = 2.4 Hz, 1H), 4.43 (q, *J* = 7.1 Hz, 2H), 3.94 (s, 3H), 1.44 (t, *J* = 7.1 Hz, 3H). ^{13}C NMR (126 MHz, $CDCl_3$) δ 166.55, 158.09, 145.48, 135.06, 134.20, 130.08, 129.83, 129.03, 128.95, 127.44, 126.94, 126.08, 125.66, 119.38, 105.55, 60.93, 55.31, 14.36. HRMS (pos. ESI) *m/z*: calcd for $C_{20}H_{18}O_3Na$ $[M+Na]^+$ 329.1154, found 329.1150.

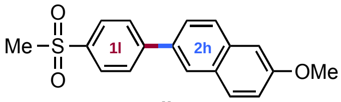

3lh

Colorless solid, isolated yield 91%; ^{1}H NMR (500 MHz, DMSO) δ 8.27 (d, *J* = 1.5 Hz, 1H), 8.04 (q, *J* = 8.7 Hz, 4H), 7.95 (dd, *J* = 8.8, 3.3 Hz, 2H), 7.86 (dd, *J* = 8.6, 1.9 Hz, 1H), 7.38 (d, *J* = 2.5 Hz, 1H), 7.23 (dd, *J* = 8.9, 2.5 Hz, 1H), 3.90 (s, 3H), 3.27 (s, 3H). ^{13}C NMR (126 MHz, DMSO) δ 157.97, 145.09, 139.26, 134.26, 133.29, 130.07, 128.65, 127.70, 127.50, 126.21, 125.34, 119.33, 105.80, 55.32, 43.67. HRMS (pos. ESI) *m/z*: calcd for $C_{18}H_{16}O_3SNa$ $[M+Na]^+$ 335.0718, found 335.0710.

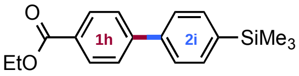

3hi

Colorless solid, isolated yield 93%; ^{1}H NMR (500 MHz, $CDCl_3$) δ 8.14 (d, *J* = 8.6 Hz, 2H), 7.68 (d, *J* = 8.6 Hz, 2H), 7.64 (d, *J* = 1.8 Hz, 4H), 4.42 (q, *J* = 7.1 Hz, 2H), 1.43 (t, *J* = 7.1 Hz, 3H), 0.33 (s, 9H). ^{13}C NMR (126 MHz, $CDCl_3$) δ 166.61, 145.58, 140.56, 140.49, 134.06, 130.19, 129.44, 127.10, 126.68, 61.06, 14.49, –1.01. HRMS (pos. ESI) *m/z*: calcd for $C_{18}H_{22}O_2SiNa$ $[M+Na]^+$ 321.1287, found 321.1286.

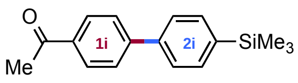

3ii

Colorless solid, isolated yield 92%; ^{1}H NMR (500 MHz, $CDCl_3$) δ 8.04 (d, J = 8.5 Hz, 2H), 7.70 (d, J = 8.5 Hz, 2H), 7.64 (d, J = 2.6 Hz, 4H), 2.64 (s, 3H), 0.33 (s, 9H). ^{13}C NMR (126 MHz, $CDCl_3$) δ 197.82, 145.85, 140.78, 140.30, 136.04, 134.10, 129.05, 127.33, 126.67, 26.78, –1.01. All spectral data match those previously reported.[13]

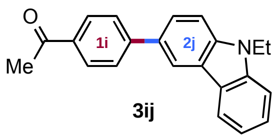

3ij

Colorless solid, isolated yield 94%; ^{1}H NMR (500 MHz, $CDCl_3$) δ 8.38 (d, J = 1.5 Hz, 1H), 8.18 (d, J = 7.7 Hz, 1H), 8.06 (d, J = 8.2 Hz, 2H), 7.81 (d, J = 8.1 Hz, 2H), 7.75 (d, J = 8.5 Hz, 1H), 7.52 (dd, J = 11.2, 4.1 Hz, 1H), 7.46 (t, J = 9.0 Hz, 2H), 7.29 (t, J = 7.4 Hz, 1H), 4.38 (q, J = 7.0 Hz, 2H), 2.65 (s, 3H), 1.46 (t, J = 7.2 Hz, 3H). ^{13}C NMR (126 MHz, $CDCl_3$) δ 197.88, 146.85, 140.55, 140.05, 135.10, 130.78, 129.08, 127.15, 126.18, 125.19, 123.67, 123.07, 120.63, 119.31, 119.27, 108.97, 108.84, 37.77, 26.72, 13.92. HRMS (pos. ESI) *m/z*: calcd for $C_{22}H_{19}ONNa$ $[M+Na]^+$ 336.1364, found 336.1360.

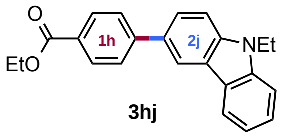

3hj

Colorless solid, isolated yield 91%; ^{1}H NMR (500 MHz, $CDCl_3$) δ 8.38 (d, J = 1.5 Hz, 1H), 8.20–8.14 (m, 3H), 7.80 (d, J = 8.5 Hz, 2H), 7.75 (dd, J = 8.5, 1.8 Hz, 1H), 7.54–7.42 (m, 3H), 7.29 (t, J = 7.4 Hz, 1H), 4.44 (q, J = 7.1 Hz, 2H), 4.39 (q, J = 7.3 Hz, 2H), 1.46 (dt, J = 12.9, 6.6 Hz, 6H). ^{13}C NMR (126 MHz, $CDCl_3$) δ 166.85, 146.64, 140.56, 140.01, 131.05, 130.21, 128.40, 127.04, 126.15, 125.25, 123.67, 123.12, 120.66, 119.29, 119.27, 108.94, 108.82, 60.99, 37.79, 14.53, 13.94. HRMS (pos. ESI) *m/z*: calcd for $C_{23}H_{21}O_2NNa$ $[M+Na]^+$ 366.1470, found 366.1469.

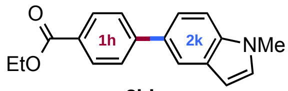

3hk

Colorless solid, isolated yield 86%; ^{1}H NMR (500 MHz, $CDCl_3$) δ 8.12 (dd, J = 8.4, 1.9 Hz, 2H), 7.91 (s, 1H), 7.76–7.71 (m, 2H), 7.52 (d, J = 8.5 Hz, 1H), 7.40 (d, J = 8.5 Hz, 1H), 7.10 (d, J = 3.1 Hz, 1H), 6.57 (d, J = 3.0 Hz, 1H), 4.42 (q, J = 7.1 Hz, 2H), 3.83 (s, 3H), 1.43 (t, J = 7.1 Hz, 3H). ^{13}C NMR (126 MHz, $CDCl_3$) δ 166.90, 147.14, 136.78, 131.64, 130.09, 129.89, 129.12, 128.28, 127.15, 121.36, 119.86, 109.75, 101.67, 60.95, 33.07, 14.52. HRMS (pos. ESI) *m/z*: calcd for $C_{18}H_{17}O_2NNa$ $[M+Na]^+$ 302.1157, found 302.1154.

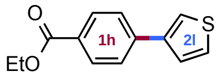

3hl

Colorless solid, isolated yield 81%; ^{1}H NMR (300 MHz, $CDCl_3$) δ 8.07 (d, J = 8.5 Hz, 2H), 7.66 (d, J = 8.5 Hz, 2H), 7.56 (dd, J = 2.6, 1.7 Hz, 1H), 7.46–7.38 (m, 2H), 4.39 (q, J = 7.1 Hz, 2H), 1.41 (t, J = 7.1 Hz, 3H). ^{13}C NMR (75 MHz, $CDCl_3$) δ 166.55, 141.32, 140.01, 130.23, 129.03, 126.72, 126.25, 126.21, 121.88, 60.90, 14.24. All spectral data match those previously reported.[14]

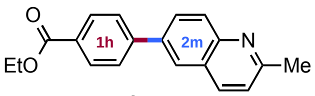

3hm

Colorless solid, isolated yield 87%; ^{1}H NMR (500 MHz, $CDCl_3$) δ 8.14 (d, *J* = 8.4 Hz, 2H), 8.09 (dd, *J* = 8.3, 6.5 Hz, 2H), 7.97 (d, *J* = 1.9 Hz, 1H), 7.94 (dd, *J* = 8.7, 2.1 Hz, 1H), 7.75 (d, *J* = 8.4 Hz, 2H), 7.30 (d, *J* = 8.4 Hz, 1H), 4.40 (q, *J* = 7.1 Hz, 2H), 2.76 (s, 3H), 1.41 (t, *J* = 7.1 Hz, 3H). ^{13}C NMR (126 MHz, $CDCl_3$) δ 166.36, 159.43, 147.30, 144.61, 137.23, 136.55, 130.13, 129.47, 129.13, 128.85, 127.15, 126.54, 125.72, 122.57, 60.99, 25.28, 14.32. HRMS (pos. ESI) *m/z*: calcd for $C_{19}H_{17}O_2NNa$ $[M+Na]^+$ 314.1157, found 314.1159.

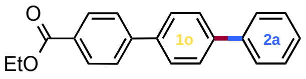

3oa

Colorless solid, isolated yield 90%; ^{1}H NMR (500 MHz, $CDCl_3$) δ 8.15 (d, *J* = 8.3 Hz, 2H), 7.72 (d, *J* = 7.2 Hz, 6H), 7.66 (d, *J* = 7.3 Hz, 2H), 7.48 (t, *J* = 7.6 Hz, 2H), 7.39 (t, *J* = 7.4 Hz, 1H), 4.43 (q, *J* = 7.1 Hz, 2H), 1.44 (t, *J* = 7.1 Hz, 3H). ^{13}C NMR (126 MHz, $CDCl_3$) δ 166.45, 144.91, 140.93, 140.38, 138.80, 130.08, 129.25, 128.83, 127.58, 127.51, 127.01, 126.79, 60.94, 14.33. HRMS (pos. ESI) *m/z*: calcd for $C_{21}H_{18}O_2Na$ $[M+Na]^+$ 325.1205, found 325.1206.

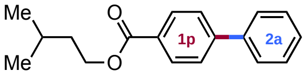

3pa

Colorless oil, isolated yield 90%; ^{1}H NMR (500 MHz, $CDCl_3$) δ 8.13 (d, *J* = 8.4 Hz, 2H), 7.67 (d, *J* = 8.5 Hz, 2H), 7.63 (d, *J* = 7.0 Hz, 2H), 7.47 (t, *J* = 7.5 Hz, 2H), 7.40 (t, *J* = 7.4 Hz, 1H), 4.40 (t, *J* = 6.8 Hz, 2H), 1.84 (dp, *J* = 13.3, 6.7 Hz, 1H), 1.70 (q, *J* = 6.8 Hz, 2H), 1.01 (d, *J* = 6.7 Hz, 6H). ^{13}C NMR (126 MHz, $CDCl_3$) δ 166.58, 145.55, 140.09, 130.08, 129.31, 128.94, 128.13, 127.30, 127.04, 63.67, 37.49, 25.28, 22.57. HRMS (pos. ESI) *m/z*: calcd for $C_{18}H_{20}O_2Na$ $[M+Na]^+$ 291.1361, found 291.1357.

Friedel–Crafts-Type Arene Borylation followed by synthesis of *p*-terphenyl derivative.[14] **(Figure 2b):** *Friedel–Crafts-Type arene borylation procedure.* Step 1: In a Schlenk tube, Et_3N (0.146 mL, 1.05 mmol) was dissolved in CH_2Cl_2, followed by slow addition of CatBCl (154 mg, 1.0 mmol). Powdered $AlCl_3$ (147 mg, 1.1 mmol) was added to the reaction mixture and the mixture stirred vigorously until all $AlCl_3$ had dissolved. *N*,*N*-dimethylaniline (0.12 mL, 1.0 mmol) was then added to the mixture and stirring continued until the borylation reaction was complete (1 h). Step 2: Excess Et_3N (2.08 mL, 15.0 mmol) followed by pinacol (355 mg, 3.0 mmol) were added to the reaction mixture and stirred for 1 h. (Caution, this is a strongly exothermic reaction.) Volatiles were removed under vacuum and the product was extracted with hexane and filtered through a short plug of silica. Removal of the solvent yielded the desired boronated compound (205.1 mg, 83%). *Synthesis of p-terphenyl derivative*. Preparation of aryltrimethylammonium triflates and Stille coupling was performed with the standard procedure as described above.

Cross-coupling between benznyltrimethylammonium triflates 4a and ArSnMe$_3$ 2j (Figure 2d). This reaction was performed with the standard procedure as described above. **5aj** was obtained as colorless solid, isolated yield 95%; ^{1}H NMR (500 MHz, CDCl$_3$) δ 8.04 (dd, *J* = 7.8, 0.5 Hz, 1H), 7.91 (s, 1H), 7.43 (t, *J* = 7.6 Hz, 1H), 7.36 (d, *J* = 8.2 Hz, 1H), 7.32–7.21 (m, 6H), 7.18 (t, *J* = 7.4 Hz, 2H), 4.31 (q, *J* = 7.2 Hz, 2H), 4.16 (s, 2H), 1.39 (t, *J* = 7.2 Hz, 3H). ^{13}C NMR (126 MHz, CDCl$_3$) δ 142.36, 140.30, 138.73, 131.60, 129.01, 128.53, 126.99, 126.01, 125.63, 123.19, 122.90, 120.68, 120.52, 118.70, 108.51, 42.08, 37.60, 13.92. HRMS (pos. ESI) *m/z*: calcd for $C_{21}H_{20}N$ $[M+H]^+$ 286.1596, found 286.1592.

Stoichiometric reaction of aryltrimethylammonium triflate 1m with Ni(cod)$_2$ and ICy ligand (Table 2): A Schlenk tube was charged aryltrimethylammonium triflates **1m** (75.1 mg, 0.2 mmol), Ni(cod)$_2$ (55.0 mg, 0.2 mmol), ICy•HBF$_4$ (128.1 mg, 0.4 mmol) or ICy (0.4 mmol, prepared from ICy•HCl and NaO^{t}Bu),[15] CsF (911.4 mg, 6 mmol) or without CsF and dioxane (20 mL) under a nitrogen atmosphere. The reaction mixture was stirred over 6 h at r.t., 50 °C, or 80 °C, respectively, and then quenched by DCl (35% in D$_2$O). The resulting mixture was extracted with ethyl acetate. The extract was dried over Na$_2$SO$_4$, filtered and removal of the solvent to give GC yields with dodecane as an internal standard. Purification by column chromatography on sillca gel to give 4-methyl-1,1'-biphenyl-4'-*d* as colorless solid (27.1 mg, 80%, D%: 80%); ^{1}H NMR (500 MHz, MeOD) δ 7.60–7.55 (m, 2H), 7.51–7.47 (m, 2H), 7.40 (dt, *J* = 6.3, 3.6 Hz, 2H), 7.29 (ddd, *J* = 7.4, 4.0, 1.2 Hz, 0.2H), 7.24 (dd, *J* = 8.4, 0.5 Hz, 2H), 2.37 (s, 3H).

Preparation and isolation of *trans*-Ni(ICy)$_2$Ar$^{(1m)}$F and related reactions (Figure 3a): A Schlenk tube was charged aryltrimethylammonium triflates **1m** (75.1 mg, 0.2 mmol), Ni(cod)$_2$ (55.0 mg, 0.2 mmol), ICy•HBF$_4$ (128.1 mg, 0.4 mmol), CsF (911.4 mg, 6 mmol) and dioxane (20 mL) under a nitrogen atmosphere. The reaction mixture was stirred 80 °C overnight and then cooled to room temperature. The resulting mixture was filtered off and dried in vacuo. 20 mL toluene was added under a nitrogen atmosphere and the mixture was stirred for 10 min, then filtered off and dried in vacuo again. The remaining solid was suspended in 20 mL pentane and filtered off to give *trans*-Ni(ICy)$_2$Ar$^{(1m)}$F as a yellow powder (103 mg, 72%). ^{1}H NMR (500 MHz, CDCl$_3$) δ 7.29 (d, *J* = 8.1 Hz, 2H), 7.10 (d, *J* = 8.0 Hz, 2H), 6.90 (d, *J* = 8.0 Hz, 2H), 6.79 (d, *J* = 8.2 Hz, 2H), 6.75 (s, 4H), 5.65 (ddd, *J* = 15.4, 7.8, 3.7 Hz, 4H), 2.46 (d, *J* = 11.9 Hz, 4H), 2.31 (s, 3H), 1.91 (s, 8H), 1.76 (dd, *J* = 26.8, 11.7 Hz, 8H), 1.68–1.57 (m, 4H), 1.56–1.39 (m, 12H), 1.24–1.15 (m, 4H). ^{13}C NMR (126 MHz, CDCl$_3$) δ 181.53, 151.37, 139.83, 139.73, 135.24, 132.85, 129.12, 126.02, 122.85, 116.07, 58.97, 34.26, 34.16, 26.01, 25.92, 25.67, 20.97. ^{19}F NMR (471 MHz, CDCl$_3$) δ –328.17. HRMS (pos. ESI) *m/z*: calcd for $C_{43}H_{59}N_4Ni$ $[M-F]^+$ 689.4094, found 689.4091. *Crystals of this Ni complex suitable for X ray diffraction have been obtained by recrystallization in acetone/pentane at –30 °C.*

To a dioxane solution of *trans*-Ni(ICy)$_2$Ar$^{(1m)}$F (71.0 mg, 0.1 mmol) prepared as described above was added PhSnMe$_3$ (72.3 mg, 0.3 mmol) under a nitrogen atmosphere. The reaction mixture was stirred 80 °C overnight and then cooled to room temperature followed by extraction with ethyl acetate. The extract was dried over Na$_2$SO$_4$, filtered and removal of the solvent to give **3ma** with 12% GC yields by dodecane as an internal standard.

To a dioxane solution of *trans*-Ni(ICy)$_2$Ar$^{(1m)}$F (71.0 mg, 0.1 mmol) was added DCl solution (0.2 mol/L) and then extracted with ethyl acetate. The extract was dried over Na$_2$SO$_4$, filtered and removal of the solvent. Purification by recrystallization twice provided *trans*-Ni(ICy)$_2$Ar$^{(1m)}$Cl as a yellow powder (116.2 mg, 80%). ^{1}H NMR (500 MHz, CDCl$_3$) δ^{1}H NMR (500 MHz, CDCl$_3$) δ 7.34 (t, *J* = 8.2 Hz, 4H), 7.11 (d, *J* = 8.0 Hz, 2H), 6.95 (d, *J* = 8.0 Hz, 2H), 6.71 (s, 4H), 6.01 (ddd, *J* = 12.1, 8.6, 3.7 Hz, 4H), 2.62 (d, *J* = 12.1 Hz, 4H), 2.31 (s, 3H), 1.97 (t, *J* = 11.2 Hz, 8H), 1.87 (t, *J* = 14.5 Hz, 8H), 1.77–1.63 (m, 8H), 1.53–1.42 (m, 8H), 1.32–1.23 (m, 4H). ^{13}C NMR (126 MHz, CDCl$_3$) δ 182.51, 155.52, 139.21, 137.55, 135.30, 132.49, 129.15, 125.94, 123.25, 116.28, 59.53, 34.49, 33.76, 26.19, 25.65, 20.98. HRMS (pos. ESI) *m/z*: calcd for $C_{43}H_{59}N_4Ni$ $[M-Cl]^+$ 689.4094, found 689.4092. *Crystals of this Ni complex suitable for X ray diffraction have been obtained by recrystallization via vapor diffusion method (CDCl$_3$/pentane) at 0 °C.*

Influence of fluoride in the coupling reaction (Table 3): A Schlenk tube was charged **1h** (178.7 mg, 0.5 mmol), Ni(cod)$_2$ (13.8 mg, 0.05 mmol), ICy•HBF$_4$ (32.0 mg, 0.1 mmol) + CsF (227.9 mg, 1.5 mmol) or ICy (0.1 mmol, prepared from ICy•HBF$_4$ and NaO^{t}Bu),[15] PhSnMe$_3$ (132.5 mg, 0.55 mmol) and dioxane (5 mL) under a nitrogen atmosphere. The reaction mixture was stirred at r.t. / 50 °C / 80 °C overnight and then cooled to room temperature. Water (10 mL) was added. The resulting mixture was extracted with ethyl acetate. The extract was dried over Na$_2$SO$_4$, filtered and removal of the solvent to give GC yields of **3ha** with dodecane as an internal standard.

Supplementary Reference

1. Xie, L. & Wang, Z. Nickel-catalyzed cross-Coupling of aryltrimethylammonium iodides with organozinc reagents. *Angew. Chem. Int. Ed.* **50**, 4901–4904 (2011).
2. Bandari, R., Hoche, T., Prager, A., Dirnberger, K. & Buchmeiser, M. R. Ring-opening metathesis polymerization based pore-size-selective functionalization of glycidyl methacrylate based monolithic media: access to size-stable nanoparticles for ligand-free metal catalysis. *Chem. Eur. J.* **16**, 4650–4658 (2010).
3. Liu, W., Cao, H., Zhang, H., Zhang, H., Chung, K., He, C., Wang, H., Kwong, F. & Lei, A. Organocatalysis in cross-coupling: DMEDA-catalyzed direct C−H arylation of unactivated benzene. *J. Am. Chem. Soc.* **132**, 16737–16740 (2010).
4. Liu, Q., Lan, Y., Liu, J., Li, G., Wu, Y. & Lei, A. Revealing a second transmetalation step in the Negishi coupling and its competition with reductive elimination: improvement in the interpretation of the mechanism of biaryl syntheses. *J. Am. Chem. Soc.* **131**, 10201–10210 (2009).
5. Watson, D. A., Su, M., Teverovskiy, G., Zhang, Y., García-Fortanet, J., Kinzel, T. & Buchwald, S. L. Formation of ArF from LPdAr(F): catalytic conversion of aryl triflates to aryl fluorides. *Science* **325**, 1661–1664 (2009).
6. Amatore, M. & Gosmini, C. Efficient cobalt-catalyzed formation of unsymmetrical biaryl compounds and its application in the synthesis of a sartan intermediate. *Angew. Chem. Int. Ed.* **47**, 2089–2092 (2008).
7. Fairlamb, L. J. S., Kapdi, A. R. & Lee, A. F. η^2-dba complexes of Pd(0): the substituent effect in Suzuki−Miyaura coupling *Org. Lett.* **6**, 4435–4438 (2004).
8. Izquierdo, F., Corpet, M. & Nolan, S. P. The Suzuki–Miyaura reaction performed using a palladium–N-heterocyclic carbene catalyst and a weak inorganic base. *Eur. J. Org. Chem.* **9**, 1920–1924 (2015).
9. Guan, B., Wang, Y., Li, B., Yu, D. & Shi, Z. Biaryl construction *via* Ni-catalyzed C−O activation of phenolic carboxylates. *J. Am. Chem. Soc.* **130**, 14468–14470 (2008).
10. Wu, D., Tao, J. & Wang, Z. Highly efficient pincer nickel catalyzed cross-coupling of aryltrimethylammonium triflates with arylzinc reagents. *J. Org. Chem. Front.* **2**, 265–273 (2015).
11. Piller, F. M., Appukkuttan, P., Gavryushin, A. Helm, M. & Knochel, P. Convenient preparation of polyfunctional aryl magnesium reagents by a direct magnesium insertion in the presence of LiCl. *Angew. Chem. Int. Ed.* **47**, 6802–6806 (2008).
12. Li, B., Li, Y., Lu, X., Liu, J., Guan, B. & Shi, Z. Cross- coupling of aryl/alkenyl pivalates with organozinc reagents through nickel-catalyzed C–O bond activation under mild reaction conditions. *Angew. Chem. Int. Ed.* **47**, 10124–10127 (2008).
13. Bernhardt, S., Manolikakes, G., Kunz, T. & Knochel, P. Preparation of solid salt-stabilized functionalized organozinc compounds and their application to cross-coupling and carbonyl addition reactions. *Angew. Chem. Int. Ed.* **50**, 9205–9209 (2011).
14. Grosso, A. D., Singleton, P. J., Muryn, C. A. & Ingleson, M. J. Pinacol boronates by direct arene borylation with borenium cations. *Angew. Chem. Int. Ed.* **50**, 2102–2106 (2011).
15. Ortega, N., Richter, C. & Glorius, F. N-Formylation of amines by methanol activation *Org. Lett.* **15**, 1776–1779 (2013).
