# Supplementary material for: Stille coupling via C–N bond cleavage
Source: Nat Commun. 2016 Sep 30;7:12937. doi: 10.1038/ncomms12937 (PMC5056441; doi:10.1038/ncomms12937)
Supplement: Supplementary Data 1 — Energy Profiles for DFT Calculations and Cartesian Coordinates [file ncomms12937-s2.docx]

**Energy Profiles for DFT Calculations**

Unit: a.u.

|  | **PhNMe_3_^+^ F^-^** | | **Ni(ICy)_2_** | | | **ICy** | | | **PhSnMe_3_** | |
| --- | --- | --- | --- | --- | --- | --- | --- | --- | --- | --- |
| G-corr_B3LYP | 0.182852 | | 0.678576 | | | 0.329849 | | | 0.155362 | |
| H-corr_B3LYP | 0.229755 | | 0.786099 | | | 0.390390 | | | 0.213890 | |
| SPE_M06 | -505.715496 | | -1561.404235 | | | -695.177409 | | | -354.549550 | |
| G-SP | -505.532644 | | -1560.725659 | | | -694.847561 | | | -354.394188 | |
| H-SP | -505.485741 | | -1560.618136 | | | -694.787020 | | | -354.335659 | |
|  | **CP0** | | **TS0** | | | **CP1** | | | **TS1** | |
| G-corr_B3LYP | 0.892783 | | 0.891170 | | | 0.892182 | | | 0.890755 | |
| H-corr_B3LYP | 1.018613 | | 1.017100 | | | 1.018133 | | | 1.016471 | |
| SPE_M06 | -2067.155835 | | -2067.138071 | | | -2067.159958 | | | -2067.152294 | |
| G-SP | -2066.263052 | | -2066.246900 | | | -2066.267776 | | | -2066.261539 | |
| H-SP | -2066.137221 | | -2066.120971 | | | -2066.141825 | | | -2066.135823 | |
|  | **CP2-1** | **NMe_3_** | | | **RS_trans** | | **CP2-2** | | | **TS2** |
| G-corr_B3LYP | 0.770788 | 0.094901 | | | 0.768423 | | 0.593418 | | | 0.595808 |
| H-corr_B3LYP | 0.887577 | 0.127512 | | | 0.887615 | | 0.709506 | | | 0.708733 |
| SPE_M06 | -1892.813679 | -174.382774 | | | -1892.827867 | | -1552.153669 | | | -1552.149444 |
| G-SP | -1892.042891 | -174.287874 | | | -1892.059443 | | -1551.560251 | | | -1551.553636 |
| H-SP | -1891.926101 | -174.255263 | | | -1891.940252 | | -1551.444163 | | | -1551.440711 |
|  | **CP3-1** | **FSnMe_3_** | | | **CP3-2** | | **TS3** | | | **PhPh** |
| G-corr_B3LYP | 0.588451 | 0.076619 | | | 0.490273 | | 0.492591 | | | 0.148765 |
| H-corr_B3LYP | 0.709321 | 0.121664 | | | 0.585638 | | 0.584327 | | | 0.191835 |
| SPE_M06 | -1552.148813 | -222.914261 | | | -1329.200012 | | -1329.198601 | | | -463.042352 |
| G-SP | -1551.560362 | -222.837642 | | | -1328.709739 | | -1328.706009 | | | -462.893587 |
| H-SP | -1551.439492 | -222.792596 | | | -1328.614374 | | -1328.614274 | | | -462.850517 |
|  | **CP2-2*^OTf^*** | | | **TS2*^OTf^*** | | | | **CP3-1*^OTf^*** | | |
| G-corr_B3LYP | 0.609638 | | | 0.614128 | | | | 0.606166 | | |
| H-corr_B3LYP | 0.744879 | | | 0.743715 | | | | 0.743984 | | |
| SPE_M06 | -2413.712716 | | | -2413.680473 | | | | -2413.687946 | | |
| G-SP | -2413.103078 | | | -2413.066345 | | | | -2413.081780 | | |
| H-SP | -2412.967837 | | | -2412.936758 | | | | -2412.943962 | | |

**Cartesian Coordinates**

Main Reaction Route

**PhNMe_3_·F^–^**

C 2.631491560491 -1.223660473116 -0.000000541708

C 1.238941536821 -1.193301207107 0.000001862718

C 2.689618700007 1.183690633459 -0.000008747256

C 0.564430656421 0.033194442491 -0.000001048466

C 1.291655955694 1.223442271708 -0.000006358334

C 3.364301151155 -0.034408311639 -0.000005865436

H 0.791081670505 2.182526400809 -0.000008673575

H 3.142495806467 -2.182001923190 0.000001765540

H 3.244460300392 2.117372970546 -0.000012885717

H 4.449955450001 -0.060291171774 -0.000007733025

N -0.925478258477 0.032187547255 0.000001648511

C -1.560590338111 1.411083484952 -0.000001824916

H -2.652401016089 1.169191661190 0.000000964709

H -1.241041496549 1.943097491724 0.898175273379

H -1.241044757179 1.943091470944 -0.898183648935

C -1.487514183328 -0.678077975991 -1.227940059882

H -1.124761481220 -1.706683791913 -1.233245477353

H -2.590229629201 -0.604924452373 -1.068014107601

H -1.124960963970 -0.149919453058 -2.112562063736

C -1.487509737632 -0.678069747008 1.227950149421

H -1.124953292243 -0.149905314799 2.112567302028

H -2.590225761010 -0.604917280025 1.068027712570

H -1.124757033079 -1.706675534755 1.233261133804

H 0.688346776976 -2.127325845100 0.000005997600

F -3.832015616840 -0.061521893229 0.000007225661

**PhSnMe_3_**

C 2.739788037458 2.587090145895 1.120684782866

H 1.805141356341 2.443316865263 1.657688778748

C 2.767532108728 2.480803356246 -0.272201720976

H 1.840051143736 2.249511705737 -0.793742208354

C 3.959358824834 2.659791293696 -0.997001830362

C 3.911991025627 2.874742335416 1.822391454420

H 3.893791262083 2.957122578907 2.906234248133

C 5.127472655953 2.945534982370 -0.268093176121

C 5.108034001005 3.053379362173 1.124966683074

H 6.074851131439 3.083627370759 -0.786642623177

H 6.025590637211 3.274269470259 1.665251181445

Sn 3.992053631262 2.513665560708 -3.143934115938

C 5.960794708073 1.903780874396 -3.760188013153

H 6.010079532349 1.808240988739 -4.850051786107

H 6.718451671861 2.632602036725 -3.453382990710

H 6.223601427199 0.935607820046 -3.321868011344

C 3.517142207985 4.418653268978 -4.028211768598

H 3.527827389469 4.350688529409 -5.121390370806

H 2.523236967587 4.756558966919 -3.717184814787

H 4.243180033420 5.180592981763 -3.726474094964

C 2.535588069196 1.053886707356 -3.757229144333

H 2.522520870205 0.956913884753 -4.848005228955

H 2.766307671894 0.072333426851 -3.330827743850

H 1.528093235072 1.336017896878 -3.433591895253

**ICy**

C -1.860158417904 -0.198193590639 0.196619372035

N -3.199531744613 0.014030882732 -0.016809920144

N -1.462964845901 1.006786093749 0.720767290032

C -3.612814690788 1.290641825808 0.357460339774

C -2.507158776387 1.922797445164 0.826715903745

H -4.629824680931 1.641714643758 0.264669169065

H -2.388537871911 2.923225487390 1.215558240132

C -4.065562820245 -1.023447742452 -0.581606269683

C -5.162879051520 -1.459943064968 0.405072160664

C -4.668107136493 -0.599571791306 -1.932587455500

H -3.387488522152 -1.867131771980 -0.748427693966

C -6.032834306308 -2.577764381060 -0.192485837555

H -5.798247315397 -0.596114739990 0.650072100213

H -4.696823706674 -1.787075946404 1.342172501035

C -5.538725118763 -1.718436107575 -2.527104776945

H -5.281834732921 0.302335118000 -1.791942790945

H -3.858301111552 -0.328324125793 -2.620373502722

C -6.633261103967 -2.165075423763 -1.545374716978

H -6.828308867425 -2.849220300862 0.512761654228

H -5.417283470335 -3.478866124125 -0.329745500064

H -5.985060481332 -1.382375432920 -3.471391712376

H -4.900345763616 -2.579590101327 -2.773301865659

H -7.211584897665 -2.993930826751 -1.972849994603

H -7.340819321857 -1.336809298209 -1.390230535991

C -0.073751098965 1.259463299294 1.110328284461

C 0.567186554465 2.376554496946 0.268041463776

C 0.060121153582 1.550169470949 2.615485551593

H 0.435555376997 0.315790529003 0.887866891198

C 2.035963001182 2.597264017086 0.664784295214

H 0.006501717391 3.311472735245 0.414502782883

H 0.484499708153 2.120042347176 -0.794851977799

C 1.529678434641 1.771879917738 3.008898182223

H -0.522694573803 2.448364630161 2.867361844028

H -0.374954542017 0.719738790545 3.184255327261

C 2.178727853441 2.882152905781 2.167969994553

H 2.462035220165 3.419804795053 0.076802101673

H 2.616960921355 1.698726630823 0.410201715156

H 1.598048963283 2.011116245531 4.077506593753

H 2.087071189064 0.834988793993 2.863052217353

H 3.237526676123 2.990952395207 2.435010882662

H 1.696363221618 3.842995622995 2.402122142235

**Ni(ICy)_2_**

C -1.860158417904 -0.198193590639 0.196619372035

N -3.199531744613 0.014030882732 -0.016809920144

N -1.462964845901 1.006786093749 0.720767290032

C -3.612814690788 1.290641825808 0.357460339774

C -2.507158776387 1.922797445164 0.826715903745

H -4.629824680931 1.641714643758 0.264669169065

H -2.388537871911 2.923225487390 1.215558240132

C -4.065562820245 -1.023447742452 -0.581606269683

C -5.162879051520 -1.459943064968 0.405072160664

C -4.668107136493 -0.599571791306 -1.932587455500

H -3.387488522152 -1.867131771980 -0.748427693966

C -6.032834306308 -2.577764381060 -0.192485837555

H -5.798247315397 -0.596114739990 0.650072100213

H -4.696823706674 -1.787075946404 1.342172501035

C -5.538725118763 -1.718436107575 -2.527104776945

H -5.281834732921 0.302335118000 -1.791942790945

H -3.858301111552 -0.328324125793 -2.620373502722

C -6.633261103967 -2.165075423763 -1.545374716978

H -6.828308867425 -2.849220300862 0.512761654228

H -5.417283470335 -3.478866124125 -0.329745500064

H -5.985060481332 -1.382375432920 -3.471391712376

H -4.900345763616 -2.579590101327 -2.773301865659

H -7.211584897665 -2.993930826751 -1.972849994603

H -7.340819321857 -1.336809298209 -1.390230535991

C -0.073751098965 1.259463299294 1.110328284461

C 0.567186554465 2.376554496946 0.268041463776

C 0.060121153582 1.550169470949 2.615485551593

H 0.435555376997 0.315790529003 0.887866891198

C 2.035963001182 2.597264017086 0.664784295214

H 0.006501717391 3.311472735245 0.414502782883

H 0.484499708153 2.120042347176 -0.794851977799

C 1.529678434641 1.771879917738 3.008898182223

H -0.522694573803 2.448364630161 2.867361844028

H -0.374954542017 0.719738790545 3.184255327261

C 2.178727853441 2.882152905781 2.167969994553

H 2.462035220165 3.419804795053 0.076802101673

H 2.616960921355 1.698726630823 0.410201715156

H 1.598048963283 2.011116245531 4.077506593753

H 2.087071189064 0.834988793993 2.863052217353

H 3.237526676123 2.990952395207 2.435010882662

H 1.696363221618 3.842995622995 2.402122142235

**CP0**

C 1.990570328190 0.812188184372 -3.335242321942

C 1.253449052381 2.027794627963 -3.221330429773

C 0.126733637956 -0.480692628883 -2.384894563826

C 0.018100208484 1.973360698737 -2.610270934725

C -0.526839010601 0.779381781021 -2.072475743160

C 1.434130417567 -0.395166461864 -2.969542946679

H 1.682119584304 2.935757449468 -3.626313228321

H -1.568806633091 0.794761264986 -1.737946534563

H 2.975306949702 0.843739485447 -3.795464801244

H -0.455028845526 -1.375136226002 -2.588531623962

H 1.975754943562 -1.315019635487 -3.187145772410

N -0.871350173391 3.201073039603 -2.520301495953

C -2.202933836138 2.920915213467 -3.203388378816

H -2.731981310012 3.873178012533 -3.299559565208

H -1.997710997388 2.496930185094 -4.187157035891

H -2.767700578332 2.238123181345 -2.550867531905

C -1.180965533979 3.499279621569 -1.065510974906

H -0.242639379884 3.518127427106 -0.512274397861

H -1.677093815991 4.473818526974 -1.026666887884

H -1.862090004177 2.704443202164 -0.722040962796

C -0.247671905552 4.404701661455 -3.148356495864

H -0.060669546388 4.205639173992 -4.204006316825

H -0.940332498791 5.240732474112 -3.043156489951

H 0.687143607713 4.633286519402 -2.635939280340

Ni 0.151792848406 -0.197857642343 -0.386995992026

F -3.126665395588 1.311703804711 -0.845741000392

C -0.242080476327 -2.076140575698 0.057888340909

N -1.486822835412 -2.650637254103 -0.087593399349

N 0.549342262975 -3.142568974272 0.415033703411

C -1.453802486240 -4.016357922551 0.161279065521

C -0.175180607238 -4.330041318062 0.476562412036

H -2.331100965975 -4.640936351855 0.101411507375

H 0.268082375707 -5.277894965754 0.738675778046

C 0.501893453573 1.073260540085 1.064798364751

N -0.306097772254 1.670335612567 2.006772980687

N 1.762781215592 1.511225538953 1.423548102827

C 0.429642100685 2.420829562395 2.917353590928

C 1.728491418964 2.327920149244 2.548213033355

H -0.024151189829 2.949638042889 3.740218655601

H 2.612423356054 2.765422696952 2.985261626124

C 1.993256831459 -3.057459442587 0.646515309164

C 2.367454799909 -3.559037518777 2.054071727746

C 2.774536860238 -3.811669736618 -0.446383997492

H 2.220321031234 -1.988596545735 0.573848905027

C 3.886214766884 -3.519704990974 2.289389232179

H 2.016590427441 -4.593539934430 2.176971817119

H 1.840368198263 -2.956351841360 2.803608904323

C 4.290212919536 -3.765253667884 -0.199421810367

H 2.444250277966 -4.860300023396 -0.465762002616

H 2.524668704715 -3.383105386960 -1.424280882120

C 4.649845913220 -4.287179859114 1.199691276659

H 4.116893752941 -3.929577394965 3.280713520185

H 4.227616204878 -2.474925224455 2.299282615884

H 4.810215861892 -4.346412674173 -0.971287021894

H 4.643664792248 -2.728769016121 -0.300245616309

H 5.731465313739 -4.210550659019 1.368501233708

H 4.397671385743 -5.356289189380 1.263767071105

C -2.708116400977 -1.927190246584 -0.487703887998

C -3.167079590373 -2.296938942647 -1.911388779214

C -3.857881049051 -2.152573698212 0.508598274706

H -2.456994773011 -0.862670024318 -0.472554346134

C -4.387779621145 -1.448226605139 -2.307782815651

H -3.421397089085 -3.367915645283 -1.951346462212

H -2.348559825113 -2.138842754263 -2.621976320461

C -5.072577980126 -1.297939682645 0.106099909713

H -4.145012300008 -3.215410253311 0.523476540239

H -3.519326923719 -1.902394591730 1.520681693888

C -5.543667991596 -1.639989914254 -1.315318717949

H -4.702916157968 -1.698738433899 -3.329363760437

H -4.091365827594 -0.392110003365 -2.283666240824

H -5.885536833767 -1.434811107502 0.831415442622

H -4.770827500522 -0.243033708880 0.116524251012

H -6.390824479643 -1.000081029080 -1.594371881039

H -5.905925987782 -2.679711151550 -1.352817305786

C -1.775887659535 1.571731098208 2.044719829656

C -2.250248254526 0.650905072301 3.183287468704

C -2.427272914828 2.960542432459 2.185565603663

H -2.082819547706 1.168312204045 1.072901789455

C -3.786667986861 0.556137865386 3.225557807859

H -1.877566404549 1.043442649051 4.142097656782

H -1.808365435552 -0.344820015529 3.054206384726

C -3.953663801716 2.822574194747 2.102168774772

H -2.152957246165 3.418004298523 3.148529706578

H -2.055782055520 3.628893359014 1.401598289395

C -4.467826964130 1.933725309683 3.244804652358

H -4.095735368866 -0.036396547001 4.096845753582

H -4.131864052833 0.015919611891 2.338252884170

H -4.426000874193 3.812846230985 2.149387294708

H -4.191588500214 2.382516005163 1.124431417039

H -5.556334026969 1.809790533041 3.175840296525

H -4.269579711817 2.431389805055 4.206971611558

C 2.977336838288 1.239691381412 0.649191492266

C 3.472782240584 2.503972399776 -0.079176499846

C 4.088793529842 0.637581236341 1.525526186505

H 2.668059167190 0.504566267588 -0.102996644578

C 4.746906393219 2.222140065491 -0.891007075725

H 3.675123030134 3.292251661838 0.661309006595

H 2.675308249860 2.868483635445 -0.736212200983

C 5.355694442710 0.352262177192 0.703328845094

H 4.341431549911 1.337981400352 2.334666452922

H 3.718802977051 -0.275511285104 2.006517167019

C 5.854176577046 1.613282165964 -0.018200815898

H 5.099306230464 3.147597270550 -1.364444438610

H 4.500355270897 1.526821187897 -1.705357523249

H 6.139904166440 -0.052744647650 1.355396640994

H 5.136389311908 -0.425151870258 -0.042992604534

H 6.734843955003 1.377794736828 -0.629186030104

H 6.179330455758 2.353115320632 0.728695871102

**TS0**

C 2.185396000000 0.433435000000 -3.437843000000

C 1.553232000000 1.578836000000 -2.978681000000

C 0.255910000000 -0.892202000000 -2.839507000000

C 0.258599000000 1.501522000000 -2.409208000000

C -0.388314000000 0.236535000000 -2.259138000000

C 1.517623000000 -0.810405000000 -3.403818000000

H 2.087004000000 2.518587000000 -3.051845000000

H -1.443182000000 0.214940000000 -1.986578000000

H 3.188376000000 0.508298000000 -3.850199000000

H -0.269996000000 -1.841461000000 -2.823015000000

H 1.988838000000 -1.694372000000 -3.825353000000

N -0.604600000000 2.770956000000 -2.388221000000

C -1.900407000000 2.519188000000 -3.147486000000

H -2.382057000000 3.487707000000 -3.307058000000

H -1.655036000000 2.053583000000 -4.102635000000

H -2.526939000000 1.887179000000 -2.500428000000

C -1.003524000000 3.186134000000 -0.988148000000

H -0.101893000000 3.268992000000 -0.386145000000

H -1.516388000000 4.151038000000 -1.067297000000

H -1.694148000000 2.407935000000 -0.628243000000

C 0.092435000000 3.928559000000 -3.036131000000

H 0.372251000000 3.664915000000 -4.056109000000

H -0.598626000000 4.772281000000 -3.042213000000

H 0.976456000000 4.194690000000 -2.456508000000

Ni 0.042536000000 -0.046135000000 -0.228728000000

F -2.927156000000 1.085539000000 -0.823166000000

C -0.476509000000 -1.927208000000 -0.053280000000

N -1.693289000000 -2.556252000000 -0.215173000000

N 0.369614000000 -2.976806000000 0.244273000000

C -1.591082000000 -3.930740000000 -0.043236000000

C -0.296170000000 -4.197519000000 0.245933000000

H -2.437572000000 -4.593171000000 -0.131743000000

H 0.194970000000 -5.135163000000 0.453852000000

C 0.603949000000 1.210745000000 1.156768000000

N -0.203648000000 1.722632000000 2.151232000000

N 1.858910000000 1.658628000000 1.525516000000

C 0.522619000000 2.439026000000 3.091843000000

C 1.819605000000 2.404576000000 2.699083000000

H 0.067127000000 2.904186000000 3.952203000000

H 2.698026000000 2.836900000000 3.152561000000

C 1.804074000000 -2.838657000000 0.509267000000

C 2.167269000000 -3.394586000000 1.899621000000

C 2.642195000000 -3.500319000000 -0.600562000000

H 1.979332000000 -1.756637000000 0.494878000000

C 3.676854000000 -3.309731000000 2.175930000000

H 1.854942000000 -4.446598000000 1.964784000000

H 1.600277000000 -2.849270000000 2.663936000000

C 4.147720000000 -3.402271000000 -0.310838000000

H 2.359872000000 -4.560096000000 -0.684419000000

H 2.399259000000 -3.026703000000 -1.558347000000

C 4.497332000000 -3.984653000000 1.066642000000

H 3.900660000000 -3.763898000000 3.149520000000

H 3.974919000000 -2.254586000000 2.251378000000

H 4.712628000000 -3.915818000000 -1.099126000000

H 4.455113000000 -2.346828000000 -0.345840000000

H 5.570715000000 -3.873644000000 1.267064000000

H 4.288800000000 -5.065091000000 1.066699000000

C -2.956158000000 -1.875910000000 -0.542809000000

C -3.408404000000 -2.149696000000 -1.989322000000

C -4.077092000000 -2.253795000000 0.440433000000

H -2.768585000000 -0.798565000000 -0.456214000000

C -4.668835000000 -1.323874000000 -2.298757000000

H -3.610322000000 -3.223842000000 -2.128700000000

H -2.604101000000 -1.883291000000 -2.685195000000

C -5.342896000000 -1.436352000000 0.132243000000

H -4.312453000000 -3.326916000000 0.364367000000

H -3.740323000000 -2.077155000000 1.469012000000

C -5.804896000000 -1.650005000000 -1.317709000000

H -4.991368000000 -1.495025000000 -3.334426000000

H -4.401359000000 -0.265769000000 -2.188910000000

H -6.142921000000 -1.699874000000 0.836658000000

H -5.111849000000 -0.372666000000 0.272010000000

H -6.680800000000 -1.022869000000 -1.529946000000

H -6.126086000000 -2.694926000000 -1.452362000000

C -1.664365000000 1.551308000000 2.187247000000

C -2.103326000000 0.637199000000 3.343635000000

C -2.394476000000 2.903682000000 2.264287000000

H -1.929315000000 1.086062000000 1.231047000000

C -3.629880000000 0.441422000000 3.338958000000

H -1.789554000000 1.080741000000 4.301238000000

H -1.592934000000 -0.329410000000 3.254250000000

C -3.909230000000 2.672387000000 2.163514000000

H -2.156807000000 3.413067000000 3.211210000000

H -2.050822000000 3.559319000000 1.456525000000

C -4.394250000000 1.774725000000 3.312472000000

H -3.930451000000 -0.155391000000 4.210083000000

H -3.906699000000 -0.136496000000 2.449609000000

H -4.441483000000 3.632575000000 2.180137000000

H -4.106867000000 2.198019000000 1.192575000000

H -5.471423000000 1.584355000000 3.221146000000

H -4.249252000000 2.300943000000 4.268822000000

C 3.057989000000 1.471603000000 0.706054000000

C 3.498416000000 2.790197000000 0.042759000000

C 4.214996000000 0.855439000000 1.510450000000

H 2.748199000000 0.771409000000 -0.078273000000

C 4.740899000000 2.584776000000 -0.837732000000

H 3.717761000000 3.533482000000 0.823306000000

H 2.665279000000 3.186062000000 -0.549918000000

C 5.458116000000 0.648862000000 0.630121000000

H 4.476740000000 1.518745000000 2.347560000000

H 3.886309000000 -0.092257000000 1.951737000000

C 5.896753000000 1.957983000000 -0.043422000000

H 5.052818000000 3.541790000000 -1.275498000000

H 4.476567000000 1.924550000000 -1.676082000000

H 6.276524000000 0.234789000000 1.232283000000

H 5.231915000000 -0.096734000000 -0.145818000000

H 6.756125000000 1.777098000000 -0.701353000000

H 6.235301000000 2.666807000000 0.727049000000

**CP1**

C 2.363983107438 0.478767219529 -3.189739029584

C 1.615132705150 1.441438850888 -2.567554678228

C 0.479789737052 -1.025934127560 -3.261961960858

C 0.226434136917 1.228270578445 -2.186169490949

C -0.326084972523 -0.091855546074 -2.520345347172

C 1.789817869882 -0.775615937863 -3.572434534785

H 2.091336437297 2.386079175158 -2.317388131170

H -1.398970883526 -0.217391413943 -2.591960789662

H 3.413073310643 0.675776375496 -3.403625238104

H 0.012379192019 -1.955019849536 -3.586294880979

H 2.383504946716 -1.498547625547 -4.125669296829

N -0.687584130111 2.520069875659 -2.465282316662

C -2.065481886878 2.133868442189 -2.941213818065

H -2.606074883144 3.062249548062 -3.149683353783

H -1.970802262262 1.549824237900 -3.857197223074

H -2.551728212697 1.595856450298 -2.110753353183

C -0.870771631966 3.297404788548 -1.191548847818

H 0.104768254378 3.453308568078 -0.731488356976

H -1.335951177019 4.258574210517 -1.437660625128

H -1.527757396247 2.684579268054 -0.562569228448

C -0.081622283889 3.400366591127 -3.517064233726

H 0.153145538084 2.796923749931 -4.394699072732

H -0.803114140759 4.179416045702 -3.775199150249

H 0.827744966907 3.866412739731 -3.140979102512

Ni -0.044894268579 0.132631376016 -0.563414147188

F -2.767994543109 1.184333300315 -0.388296117847

C -0.510485510889 -1.748941252457 -0.030519935765

N -1.718064980750 -2.380071486169 -0.208033197046

N 0.313478723278 -2.769566417503 0.381433601963

C -1.635743960649 -3.737101610353 0.066213112143

C -0.357962473021 -3.987052099919 0.433335672971

H -2.483804577924 -4.398655587314 -0.012770858004

H 0.117457032151 -4.909661060186 0.725031479067

C 0.563029965366 1.075410745066 1.099828167421

N -0.178310912089 1.386870150696 2.213045520798

N 1.844194063020 1.432599160726 1.475565452375

C 0.609654951097 1.901542019607 3.233267814719

C 1.880859764438 1.933048556088 2.773149308001

H 0.204690059504 2.198369450297 4.187528662234

H 2.790421407374 2.260748930677 3.250882193157

C 1.749209483884 -2.643000143551 0.660487201560

C 2.087886683307 -3.212499442671 2.053162793455

C 2.581162193644 -3.316447962840 -0.448333793106

H 1.943162148403 -1.566597533485 0.648897935497

C 3.597188824049 -3.194029219419 2.344643726868

H 1.737687149347 -4.251931038211 2.114285545860

H 1.536935414506 -2.650634182693 2.817309055112

C 4.085958083879 -3.272966100048 -0.142013134415

H 2.264293555729 -4.365191778058 -0.544594102181

H 2.361126649469 -2.825228151188 -1.403029396450

C 4.401534437457 -3.883211872020 1.231942702077

H 3.787788311991 -3.678582115089 3.310655041878

H 3.939895361991 -2.156124925217 2.450831516313

H 4.638334100750 -3.799838396771 -0.930212446978

H 4.433568868258 -2.230279850899 -0.166455710393

H 5.476235561525 -3.814330923960 1.443627901323

H 4.153938236484 -4.955243096196 1.216510956672

C -2.957217584266 -1.728386299440 -0.661620904470

C -3.377954180326 -2.213285943691 -2.060875484074

C -4.110932688760 -1.940235981670 0.330781995085

H -2.749095124925 -0.649102255792 -0.674104275841

C -4.621120195525 -1.444054556052 -2.542481048667

H -3.602969056712 -3.291091073382 -2.031463487450

H -2.551430912345 -2.086521692281 -2.770301428384

C -5.320789895126 -1.110128146381 -0.126976184527

H -4.388906588226 -3.004705547017 0.390349713568

H -3.788722307561 -1.637324541211 1.332263809951

C -5.776382300930 -1.535035283233 -1.532112869060

H -4.938994687903 -1.822548468024 -3.522997981792

H -4.351474813022 -0.388142020065 -2.678349420870

H -6.147371610641 -1.210407864422 0.588582974467

H -5.010837814689 -0.057313896377 -0.142393390412

H -6.616348176052 -0.911923415430 -1.866066246588

H -6.149116087939 -2.570579182727 -1.497761310637

C -1.649754633041 1.335708957967 2.301951437359

C -2.118590823876 0.408680461051 3.435415991022

C -2.219455190719 2.755499524732 2.483778245946

H -2.006797279341 0.979435163255 1.327000892437

C -3.654673452378 0.396738631157 3.542893298050

H -1.694923533429 0.743481084503 4.395225397407

H -1.740396738398 -0.606426607338 3.259395630086

C -3.752416408930 2.706138181271 2.510193081723

H -1.845711056134 3.204582399258 3.417535393543

H -1.870737669920 3.389473484048 1.660700804917

C -4.245264548437 1.810591305725 3.656336553108

H -3.960408179794 -0.218100985514 4.399679059971

H -4.070092054973 -0.079909272434 2.647364196363

H -4.163221777932 3.719251633189 2.612773984383

H -4.083669486179 2.307653840434 1.542863777351

H -5.341791323071 1.757117119802 3.658800476656

H -3.951389398944 2.256669879401 4.619354646024

C 3.017729941418 1.449473999332 0.592498456111

C 3.415141426912 2.896999197715 0.237561934775

C 4.214732478258 0.704413987312 1.207374819630

H 2.702805196692 0.932028243479 -0.317255759282

C 4.646182024937 2.943437179897 -0.682388160757

H 3.633981835061 3.445927729905 1.164746079583

H 2.562193851778 3.403483300330 -0.229738278820

C 5.431976864643 0.734824059213 0.269197442894

H 4.492115127623 1.168648208852 2.164580217133

H 3.925169082892 -0.326629201969 1.430622857960

C 5.832447338829 2.175346367934 -0.081268721683

H 4.924346356051 3.986875221033 -0.877779677605

H 4.389450714835 2.499178292798 -1.654069001893

H 6.272881431169 0.205623301715 0.734680220074

H 5.190549703725 0.190647396523 -0.655335942373

H 6.678607993924 2.176828287188 -0.779773060777

H 6.176141746004 2.688127171282 0.829490978749

**TS1**

C -2.482679182890 0.437727478098 -3.229377980821

C -1.893267850908 -0.654835616686 -2.627075771393

C -0.410761181313 1.668603486879 -3.151678830491

C -0.520628228440 -0.626913181624 -2.202862169508

C 0.213181671345 0.587576501373 -2.467872327802

C -1.739604222322 1.612325841363 -3.518193601888

H -2.495746267704 -1.540739866581 -2.446210974003

H 1.292119174735 0.573688811015 -2.348016482347

H -3.541827214260 0.398956707758 -3.480694921236

H 0.187667743738 2.541331916208 -3.410840820159

H -2.212804701049 2.442214793961 -4.036523840495

N 0.359606668001 -2.196608474699 -2.958322869301

C 1.560212255568 -1.723418160286 -3.684007583778

H 2.053823897872 -2.579176470886 -4.167179803873

H 1.262516014871 -1.000446710522 -4.447179362110

H 2.224135780014 -1.269451967174 -2.941050754546

C 0.783675819017 -3.061217654549 -1.840999949886

H -0.084073210772 -3.316087323286 -1.229006758170

H 1.241197765497 -3.982173058035 -2.232652862162

H 1.506420658289 -2.476092871232 -1.264802559167

C -0.552457018847 -2.898178171211 -3.874307234342

H -0.946118761900 -2.195037756471 -4.612368839936

H -0.020774219101 -3.710154189610 -4.389112395201

H -1.386784489693 -3.327686186127 -3.315099752901

Ni 0.022901924455 -0.017918909918 -0.485753748980

F 2.538543752333 -0.772769809068 -1.036940883561

C 0.431116220694 1.714769848728 0.475285829724

N 1.618804768140 2.399189042411 0.527223568761

N -0.462872593511 2.589992243769 1.044948476749

C 1.460320293884 3.655285185797 1.093849869147

C 0.152930649957 3.779680465552 1.417828598821

H 2.281081895211 4.341916254474 1.224932094851

H -0.376492434583 4.597958627143 1.878153806343

C -0.338633084255 -1.372274654245 0.909724465336

N 0.567077799379 -1.990042735805 1.734944584485

N -1.545787540675 -1.889769320255 1.326939483207

C -0.058100201105 -2.844007606659 2.633251108319

C -1.384142406383 -2.786545739038 2.377676753213

H 0.486051002085 -3.412887683773 3.369619630756

H -2.210105201422 -3.296768690786 2.846524973645

C -1.923165064218 2.417073566005 1.056270136782

C -2.521258477174 2.768656044461 2.432306548165

C -2.563435801092 3.252129635420 -0.070857525620

H -2.084307804642 1.355508173962 0.845703138956

C -4.058768613208 2.706018551451 2.433707114117

H -2.221244880456 3.787724560489 2.711277802635

H -2.103310638479 2.099864495749 3.195334316308

C -4.095244764721 3.154756666157 -0.056642893165

H -2.267509082330 4.303602130937 0.058108877118

H -2.161076907433 2.918643960026 -1.033238680680

C -4.670175753048 3.555214535403 1.309468780882

H -4.434450076037 3.034141917745 3.411144728453

H -4.387176208284 1.666145007525 2.310812394719

H -4.512802498877 3.787143229770 -0.849942388742

H -4.395160915239 2.123410063872 -0.294014353359

H -5.762876625231 3.453201206605 1.312493881149

H -4.454157341962 4.617956812388 1.495451015134

C 2.884619426428 1.951786912848 -0.083176393835

C 3.161619347322 2.700856610299 -1.399613278526

C 4.074490868628 2.119748387270 0.874031793563

H 2.751515105676 0.886478712671 -0.327376408657

C 4.423844633699 2.125321526581 -2.061940430406

H 3.294666325124 3.777144716430 -1.205204041217

H 2.299047511051 2.601282690734 -2.067079380822

C 5.352048837476 1.557966756429 0.225810459712

H 4.234425030273 3.183429898745 1.110578334535

H 3.866103793430 1.614262613477 1.823902833402

C 5.638631653673 2.228685357784 -1.127117883897

H 4.624824996857 2.641132370772 -3.009929996204

H 4.232783440319 1.069019064026 -2.291877382845

H 6.204911827870 1.684926843229 0.905421773981

H 5.221000917106 0.479505521972 0.066450866052

H 6.519589911219 1.770480888765 -1.595089444660

H 5.885969352925 3.289060591624 -0.963648459977

C 2.037650321638 -1.863719587656 1.664289629307

C 2.592849725534 -1.203769327636 2.938757924205

C 2.682599718858 -3.244644271796 1.435443451753

H 2.247353575222 -1.257927511308 0.771023059644

C 4.130186767623 -1.133138886187 2.916078811865

H 2.273430230505 -1.778605953444 3.821952714746

H 2.165088629912 -0.198888228239 3.044968245305

C 4.209376214049 -3.114471352845 1.349906344052

H 2.426669929438 -3.932136126685 2.256301414333

H 2.282892157453 -3.682800267571 0.514853714495

C 4.774550439629 -2.498969314946 2.637908484889

H 4.494546163343 -0.721327939151 3.866402486217

H 4.444632886182 -0.437381138456 2.130065203869

H 4.660305745008 -4.097783426313 1.163719292337

H 4.453405254557 -2.476324099511 0.490070592166

H 5.864620678208 -2.391061380741 2.566994641052

H 4.580887291243 -3.179196987122 3.481674711006

C -2.850560265665 -1.639540543304 0.702075946717

C -3.357672860000 -2.888509774140 -0.045630961489

C -3.889481790130 -1.177043965660 1.738511605053

H -2.675392189771 -0.839222349903 -0.022934894386

C -4.729496567318 -2.639844793340 -0.693921551102

H -3.437007138978 -3.724827223632 0.663702797438

H -2.618812930459 -3.185995765414 -0.798722289260

C -5.255126260620 -0.916401541946 1.083605018805

H -4.011020229249 -1.951201215286 2.509109090549

H -3.519093448611 -0.283322553222 2.251928515222

C -5.762684779297 -2.157909817396 0.335250528221

H -5.078258771810 -3.555438617930 -1.187730203533

H -4.625535882944 -1.878864663212 -1.480289534891

H -5.979863753458 -0.605280423253 1.846385673325

H -5.166729435150 -0.079711951432 0.375789797167

H -6.717641840352 -1.939996680156 -0.158920018950

H -5.959990855187 -2.962651738790 1.059012741521

**CP2-1**

C -0.170690114972 -2.456748773804 -3.424858035735

C -0.015942832200 -1.806445213059 -2.192768246822

C -1.307399874057 -0.516682650500 -4.282766989754

C -0.484887121104 -0.496753351437 -1.970142881100

C -1.123672875954 0.131909019280 -3.057767226397

C -0.829569959092 -1.816470602373 -4.475111175278

H 0.482856737257 -2.349981891210 -1.390648071087

H -1.449884667511 1.160775975390 -2.939327573429

H 0.218037922687 -3.465376979725 -3.559531268630

H -1.813229992038 -0.000398062005 -5.097762451451

H -0.963618227544 -2.319071585981 -5.430718942332

Ni -0.162685980366 0.533200963608 -0.353757500463

F -0.278292774933 2.110868222463 -1.314296508301

C 0.588088212211 1.785525903546 1.019594466664

N 1.853065647338 2.294425420736 0.945105235892

N -0.043479238063 2.577846450655 1.928204810487

C 1.992027963624 3.405285613433 1.769677630196

C 0.799134102446 3.586390711806 2.387351555124

H 2.915038742139 3.955191583742 1.860432564119

H 0.492958308836 4.322748465645 3.112857670535

C -0.516552319148 -1.013623733968 0.725030515829

N 0.276439859167 -1.749034306994 1.562403809354

N -1.743062356089 -1.617428394675 0.817631546811

C -0.437309986707 -2.787291278519 2.150655565578

C -1.704995248434 -2.701125907721 1.682463490909

H 0.009638767426 -3.487371065775 2.837996163573

H -2.569790241204 -3.311423635710 1.887860074297

C -1.505222697753 2.558340965151 2.097918820031

C -1.938085965654 2.817940801471 3.547393947552

C -2.155038509201 3.549240383552 1.113236476650

H -1.801266538230 1.542972709856 1.814099266436

C -3.472057082118 2.795409695084 3.665636333571

H -1.576296220348 3.803375925213 3.873636802815

H -1.485043413183 2.073781854358 4.214856888053

C -3.684893211337 3.537911254571 1.245500771537

H -1.776528300561 4.558757694070 1.332876381534

H -1.824306455658 3.289514110083 0.101003346583

C -4.131174448651 3.788606972630 2.694897153844

H -3.765588605220 3.016825998851 4.699326650685

H -3.836729155344 1.780281935800 3.450422043504

H -4.120332818025 4.289829496210 0.576088579124

H -4.072896964996 2.565338012350 0.909355515463

H -5.223659314687 3.724233574619 2.776178189217

H -3.855743658702 4.813069110063 2.986755770964

C 2.816864818445 1.900942358116 -0.102730978303

C 2.706780180762 2.835699782893 -1.321105144273

C 4.257587798096 1.820073608309 0.421687614747

H 2.490555846236 0.902505567363 -0.417189397177

C 3.670906588394 2.392627981319 -2.431760415718

H 2.951451757692 3.861174905877 -1.004091827194

H 1.665562081754 2.817774238034 -1.657814914173

C 5.212467243504 1.367282236448 -0.696767956061

H 4.578479686299 2.808377285404 0.781086396779

H 4.315615615969 1.137148179191 1.278539182778

C 5.117960264181 2.283828881150 -1.926889270462

H 3.611794762416 3.094596866657 -3.272465151890

H 3.344718435941 1.417439025689 -2.821298528395

H 6.240940715362 1.338661178306 -0.315201143400

H 4.959379476119 0.338083329203 -0.991976235376

H 5.776506788237 1.912298019140 -2.722311254699

H 5.486327512934 3.285172331127 -1.657189667834

C 1.702934702845 -1.500324880427 1.815483799542

C 1.957729600865 -1.221931155291 3.308326602190

C 2.573183460188 -2.659561908849 1.297551020675

H 1.932972144645 -0.599231099136 1.243060238750

C 3.449965759554 -0.987439579937 3.592147225632

H 1.611114154405 -2.078945962050 3.903144153963

H 1.361964699098 -0.355832564068 3.620121297702

C 4.062587387415 -2.408304329454 1.582522388901

H 2.264063209219 -3.593936024496 1.787882982326

H 2.399255969524 -2.791643823083 0.223279557150

C 4.316322382071 -2.145869288667 3.074854786802

H 3.601579312468 -0.846527038103 4.669473988693

H 3.766495226339 -0.051880372760 3.110354832865

H 4.653436598945 -3.265705560394 1.238031330950

H 4.403277905725 -1.542113699121 0.997041148681

H 5.378210302691 -1.930882195794 3.247499989521

H 4.084380991066 -3.056351162660 3.647366535675

C -2.949406040878 -1.212373172737 0.070969402598

C -3.411348103733 -2.316681426863 -0.895070281228

C -4.085654734366 -0.794729327587 1.019291515752

H -2.629031570514 -0.348771898615 -0.517563715801

C -4.640298456611 -1.853813910037 -1.693654114518

H -3.667684379498 -3.221337988448 -0.322856653147

H -2.587984293253 -2.573076350538 -1.567847277917

C -5.317516164713 -0.334682158803 0.221635290921

H -4.365225116060 -1.644668455250 1.659309359756

H -3.738482914871 0.002733649649 1.687637325556

C -5.785031536673 -1.411658624979 -0.769698103914

H -4.974141129735 -2.661414061146 -2.356472834010

H -4.344280182307 -1.019423750572 -2.344214462092

H -6.128116260943 -0.069378115394 0.912028530487

H -5.064737143632 0.581057825088 -0.332090940574

H -6.630231379492 -1.037304041801 -1.360870400388

H -6.156829179380 -2.282133687160 -0.208109783913

**NMe_3_**

N -2.033555881742 -1.616538224530 -0.496895070737

C -1.449159136286 -2.147474684054 -1.718695038699

H -2.040011039470 -2.973801670139 -2.164502609686

H -1.366561707035 -1.350564575003 -2.466306996557

H -0.441626679590 -2.526192099843 -1.513064341566

C -2.087317807099 -2.623492403186 0.551606959179

H -2.474183957688 -2.176823682068 1.474446842846

H -2.731519887072 -3.489557617175 0.295463809160

H -1.078887804448 -3.001585177488 0.753908829723

C -3.341571914110 -1.028697339480 -0.741311051843

H -3.255964062683 -0.233300273014 -1.490199974286

H -4.090652989056 -1.761346991656 -1.105685807756

H -3.726559733721 -0.584579372364 0.183541340223

**CP2-2**

Ni -0.204773537050 -0.048060248031 -0.367620239758

C 0.541347619197 -0.290369362334 -2.098747926151

C 1.503083101031 0.608405319881 -2.594281582218

C 2.028741233151 0.481512535134 -3.885855751725

C 1.607096840982 -0.556949585680 -4.717819470438

C 0.658849355309 -1.466444226007 -4.243928105444

C 0.145459948360 -1.337069510910 -2.949544252135

H 1.843553510070 1.437042720339 -1.976375193554

H 2.766675308745 1.198350955567 -4.241350958613

H 2.014056063783 -0.658406154599 -5.720969942274

H 0.322470240249 -2.282521727450 -4.880802280514

H -0.585233310772 -2.068320888909 -2.607516668056

Sn -1.176356336776 2.464088118393 1.606493904050

C -1.328747571371 2.075174390936 -0.553139535988

C -2.234683634399 1.103130805554 -1.043126305708

C -2.593210237789 1.041859201998 -2.402822257848

C -2.038264673997 1.938950774898 -3.304893634102

C -1.127543700598 2.905594996344 -2.852747822530

C -0.790144681811 2.973526404832 -1.505794257665

H -2.732098157267 0.439034257028 -0.339378362393

H -3.310279281774 0.297671722870 -2.741479401533

H -2.307989735415 1.896227317328 -4.356825531510

H -0.695817228966 3.610749017194 -3.559068720875

H -0.110416093595 3.757609318265 -1.177346688861

F -0.920728515967 0.182195146981 1.386107107750

C 0.733978203080 -1.575132331843 0.207784614229

N 0.147668703127 -2.768266361486 0.492560340003

N 2.038083869371 -1.723156433555 0.551117557784

C 1.078937306937 -3.652109695731 1.022348853490

C 2.266766611896 -2.998058116433 1.053273605874

H 0.825298771445 -4.655282773333 1.323829114773

H 3.239398675879 -3.326912649438 1.381996056188

C -1.299695191062 -3.023792818474 0.360173856093

C -1.585324872884 -4.398664560707 -0.262124479861

C -2.019932046192 -2.832946794051 1.705922103350

H -1.651334753743 -2.248616653186 -0.329651843846

C -3.100556456184 -4.615253482816 -0.414688237525

H -1.179776480275 -5.191436479151 0.382428482829

H -1.078827581146 -4.482918679599 -1.231539424509

C -3.530133248990 -3.076689289082 1.555243919717

H -1.601291758099 -3.537088695845 2.440160164880

H -1.818236484211 -1.816081451571 2.054792813084

C -3.831984777722 -4.445126203511 0.925929794990

H -3.289158801198 -5.611344447762 -0.833223292855

H -3.497974455189 -3.891441122778 -1.141237053839

H -4.016710748301 -2.987882939635 2.533920341863

H -3.960618651381 -2.284998092668 0.924275236501

H -4.912683596043 -4.569712948013 0.783799435512

H -3.512845093848 -5.241375281904 1.614913469186

C 3.064664511506 -0.675480439438 0.413212438807

C 3.690963616683 -0.323029734437 1.772706599772

C 4.131762076361 -1.067648620335 -0.622249660635

H 2.516173368226 0.194601557195 0.042035240548

C 4.738708504062 0.790806774829 1.615022423777

H 4.172985991595 -1.215570399842 2.197433080397

H 2.903335943690 -0.017062638192 2.469980000635

C 5.181007998444 0.046276123681 -0.769487377197

H 4.626670577813 -1.996014765084 -0.301258036524

H 3.646911472070 -1.269698658476 -1.583065139563

C 5.812134504698 0.418147160946 0.580906800078

H 5.198474752680 1.003572117247 2.587653913006

H 4.234428504743 1.716282328018 1.301578357826

H 5.953787752224 -0.267989529947 -1.481477639649

H 4.699715964755 0.933564508643 -1.205234087425

H 6.518908452377 1.247509365577 0.453500725085

H 6.396023568014 -0.435689760852 0.956126131739

C -1.395387016405 4.641331353158 1.503719514928

H -2.317998195296 4.931745650777 0.987159869313

H -1.430694219750 5.066608726569 2.514940770926

H -0.557478142666 5.117468390474 0.979297975583

C 0.756363087870 2.288189382954 2.544016626256

H 0.967446063870 3.155194075244 3.178783303764

H 0.799789633244 1.376596846274 3.146461712379

H 1.543402522462 2.228799217877 1.782760826925

C -2.900390580334 1.848294223736 2.736017540854

H -3.352719098112 2.694173502818 3.263633625851

H -3.656001139214 1.417457721590 2.069567564027

H -2.623277834509 1.079508232652 3.463394762003

**TS2**

Ni -0.211590000000 0.048103000000 -0.242710000000

C 0.732165000000 0.071262000000 -1.885200000000

C 1.673672000000 1.079271000000 -2.160178000000

C 2.377009000000 1.114350000000 -3.370433000000

C 2.151534000000 0.138166000000 -4.343062000000

C 1.217641000000 -0.869544000000 -4.090423000000

C 0.524918000000 -0.903203000000 -2.875295000000

H 1.859989000000 1.865920000000 -1.429484000000

H 3.096682000000 1.910120000000 -3.554003000000

H 2.693523000000 0.163581000000 -5.285175000000

H 1.028076000000 -1.634695000000 -4.840966000000

H -0.192970000000 -1.704180000000 -2.707292000000

Sn -0.904178000000 2.313438000000 1.317503000000

C -1.621708000000 1.381279000000 -0.866049000000

C -2.904869000000 0.829216000000 -0.630106000000

C -3.994983000000 1.089058000000 -1.467855000000

C -3.834297000000 1.921292000000 -2.574695000000

C -2.583664000000 2.495333000000 -2.832541000000

C -1.505892000000 2.229113000000 -1.992075000000

H -3.048414000000 0.196339000000 0.240965000000

H -4.965762000000 0.647742000000 -1.252405000000

H -4.676284000000 2.129211000000 -3.230770000000

H -2.452732000000 3.144237000000 -3.695445000000

H -0.544795000000 2.676203000000 -2.225719000000

F -1.097666000000 0.204803000000 1.504966000000

C 0.762900000000 -1.529622000000 0.312531000000

N 0.151106000000 -2.730784000000 0.514146000000

N 2.060583000000 -1.739711000000 0.658650000000

C 1.053430000000 -3.672090000000 0.990060000000

C 2.255633000000 -3.049952000000 1.075409000000

H 0.773275000000 -4.686877000000 1.221941000000

H 3.216220000000 -3.423554000000 1.391768000000

C -1.293164000000 -2.959535000000 0.322017000000

C -1.566600000000 -4.242226000000 -0.479056000000

C -2.041979000000 -2.946974000000 1.665594000000

H -1.631947000000 -2.102465000000 -0.269972000000

C -3.077578000000 -4.436368000000 -0.689582000000

H -1.170120000000 -5.112345000000 0.063444000000

H -1.041184000000 -4.198007000000 -1.440918000000

C -3.548944000000 -3.166361000000 1.453922000000

H -1.638633000000 -3.741386000000 2.310837000000

H -1.853247000000 -1.987015000000 2.155440000000

C -3.835556000000 -4.441732000000 0.646946000000

H -3.255824000000 -5.369918000000 -1.237027000000

H -3.461491000000 -3.624053000000 -1.323480000000

H -4.056004000000 -3.204307000000 2.425690000000

H -3.965692000000 -2.299696000000 0.920315000000

H -4.913192000000 -4.545229000000 0.469178000000

H -3.528919000000 -5.320717000000 1.233521000000

C 3.123650000000 -0.722357000000 0.589465000000

C 3.769086000000 -0.490673000000 1.966063000000

C 4.172533000000 -1.079509000000 -0.477454000000

H 2.610015000000 0.191012000000 0.280601000000

C 4.855566000000 0.593762000000 1.880925000000

H 4.221804000000 -1.427086000000 2.323043000000

H 2.996558000000 -0.209528000000 2.690703000000

C 5.261734000000 0.002570000000 -0.552692000000

H 4.633304000000 -2.046161000000 -0.225763000000

H 3.677302000000 -1.194735000000 -1.447208000000

C 5.910829000000 0.255864000000 0.816478000000

H 5.326582000000 0.721429000000 2.863176000000

H 4.384639000000 1.556567000000 1.634954000000

H 6.020409000000 -0.289619000000 -1.288906000000

H 4.811126000000 0.933666000000 -0.924116000000

H 6.646999000000 1.066083000000 0.743467000000

H 6.464368000000 -0.642611000000 1.128245000000

C -0.415622000000 4.194433000000 0.328648000000

H -1.255974000000 4.571531000000 -0.261192000000

H -0.161461000000 4.934106000000 1.098554000000

H 0.449785000000 4.087708000000 -0.334628000000

C 0.814020000000 2.125361000000 2.614936000000

H 0.953473000000 3.051959000000 3.184845000000

H 0.684683000000 1.291720000000 3.310698000000

H 1.729090000000 1.954609000000 2.037230000000

C -2.727826000000 2.650456000000 2.396984000000

H -2.640210000000 3.551672000000 3.014680000000

H -3.568454000000 2.787792000000 1.710073000000

H -2.945851000000 1.798616000000 3.047866000000

**CP3-1**

Ni -0.138888313924 0.010098406223 -0.698533552945

C 0.539912387716 -0.423066721336 -2.400708850426

C 1.615866934092 0.269367586515 -2.984434226215

C 2.162494143215 -0.120934637490 -4.212693311270

C 1.636037791836 -1.213576521021 -4.904230564557

C 0.554275534799 -1.904141045553 -4.352871450670

C 0.018501278417 -1.513343233702 -3.121496224643

H 2.034362753625 1.143126745210 -2.486862877920

H 2.995876218644 0.438978600547 -4.634253193275

H 2.054712336142 -1.515727448955 -5.861189633667

H 0.122244338976 -2.750385131324 -4.884697877556

H -0.830759275842 -2.068447446672 -2.726123776134

Sn -1.228172847396 2.214269768715 2.029884592421

C -1.212219387452 1.354111646161 -1.541593149799

C -2.593550668084 1.120860630923 -1.711874398717

C -3.461231358931 2.096407606017 -2.216039232040

C -2.971524344131 3.358283820103 -2.564356159032

C -1.607138129700 3.621449855570 -2.415989818957

C -0.749738255999 2.629269847882 -1.926177801106

H -3.011162068851 0.147980695877 -1.450755271833

H -4.519859443333 1.872536181080 -2.338814774447

H -3.640602387453 4.120247950707 -2.957781625221

H -1.208906705748 4.595238167260 -2.696969871584

H 0.314369941974 2.856328488200 -1.861257218382

F -0.971998461846 0.489839878740 1.204170470991

C 0.872249314700 -1.432028869103 0.200798839315

N 0.346554689440 -2.616795563736 0.625883024249

N 2.187451250873 -1.508355936738 0.549921136813

C 1.312183761371 -3.411931728924 1.229280130947

C 2.474772868422 -2.713832505677 1.178336786678

H 1.100520879483 -4.389500530129 1.632751126184

H 3.462018837108 -2.972661216127 1.527136292356

C -1.074394576231 -2.966016701478 0.476370339606

C -1.259548190142 -4.311043629659 -0.244332630463

C -1.806292756518 -2.935921707307 1.828429712882

H -1.481545433470 -2.169432626414 -0.156718967108

C -2.752732081809 -4.635460870125 -0.416096099178

H -0.783102135380 -5.112176532708 0.339299005871

H -0.752493583741 -4.276534081151 -1.215602190614

C -3.296021065771 -3.273329886665 1.653702854004

H -1.341323317630 -3.663225626209 2.510292417155

H -1.680424363890 -1.943081767329 2.274022615979

C -3.493948079142 -4.614037937851 0.929638962442

H -2.865241210463 -5.612870309568 -0.900820781230

H -3.206800356348 -3.897602149038 -1.093297777256

H -3.790286930130 -3.289732855881 2.632967132554

H -3.779397983816 -2.473644521751 1.073687713494

H -4.562858465498 -4.807612710409 0.776107711794

H -3.115955781951 -5.429160192020 1.564809527021

C 3.166313939964 -0.446050539595 0.274723480121

C 3.752254207444 0.131393841554 1.574574377623

C 4.270461434318 -0.927799945924 -0.680945899792

H 2.584490572854 0.332434939528 -0.228095044261

C 4.752367365961 1.259639037719 1.272734173079

H 4.262972523558 -0.666297459225 2.133404989826

H 2.937219039941 0.493383360652 2.214113098760

C 5.272567417619 0.200939936853 -0.972366340281

H 4.800800006157 -1.778731994957 -0.228609589431

H 3.810283789576 -1.282391428919 -1.609135515584

C 5.861236858495 0.789664241846 0.318685875068

H 5.183697753991 1.633541315233 2.209584887249

H 4.216077774430 2.104819198152 0.816600904881

H 6.072490132840 -0.174488593522 -1.621986939504

H 4.761818386535 0.994783155681 -1.535959789434

H 6.535579112231 1.622680869614 0.084003020681

H 6.471520166216 0.025101472428 0.822421853330

C 0.468688123412 3.402488060137 1.528915349312

H 0.415915576084 3.718410923491 0.484963316942

H 0.508556656266 4.290471244613 2.169588572917

H 1.389760323578 2.830119229219 1.678518682829

C -1.177549891207 1.636245219092 4.090183964567

H -1.302101492468 2.507723936041 4.742481797073

H -1.979658353155 0.926598003660 4.316790659530

H -0.222442763854 1.161374721860 4.336443306020

C -3.132501435936 2.974543494356 1.464735383970

H -3.332006173606 3.905002664986 2.008245917120

H -3.171416768870 3.169777893694 0.390683965010

H -3.916242243707 2.254067192686 1.718270829500

**FSnMe_3_**

Sn -1.228172847396 2.214269768715 2.029884592421

F -0.971998461846 0.489839878740 1.204170470991

C 0.468688123412 3.402488060137 1.528915349312

H 0.415915576084 3.718410923491 0.484963316942

H 0.508556656266 4.290471244613 2.169588572917

H 1.389760323578 2.830119229219 1.678518682829

C -1.177549891207 1.636245219092 4.090183964567

H -1.302101492468 2.507723936041 4.742481797073

H -1.979658353155 0.926598003660 4.316790659530

H -0.222442763854 1.161374721860 4.336443306020

C -3.132501435936 2.974543494356 1.464735383970

H -3.332006173606 3.905002664986 2.008245917120

H -3.171416768870 3.169777893694 0.390683965010

H -3.916242243707 2.254067192686 1.718270829500

**CP3-2**

C 1.323568683570 1.595106818373 -0.085426144870

C 2.058261079643 1.931312951329 1.065043323506

C 1.933575236062 1.781830329736 -1.338073871637

C 3.375535702012 2.393474444315 0.967843532010

H 1.605282737160 1.832258582928 2.048853827589

C 3.250079750205 2.246977283203 -1.435093743998

H 1.384975308580 1.559012714833 -2.250646211287

C 3.980393628468 2.549368192927 -0.282539814887

H 3.925776208536 2.643739516491 1.872996446997

H 3.702447572300 2.380135457600 -2.416094233970

C -1.068911354416 2.437266017504 0.083839685426

C -1.482822910212 2.982563109833 1.315894760764

C -1.501956969147 3.085745686429 -1.089581883786

C -2.333954615859 4.090987742401 1.373696948514

H -1.136824233517 2.539168010634 2.248958552441

C -2.353108332251 4.193775634593 -1.038318750568

H -1.168137291283 2.724751612020 -2.061302809039

C -2.774351624330 4.698189813506 0.195045784011

H -2.645691545928 4.487404199397 2.338315052727

H -2.680187776583 4.671206918546 -1.960191270080

H 5.000639097729 2.917170512298 -0.358266687636

H -3.428595500515 5.565891966721 0.237359604195

Ni -0.320450428961 0.727053478717 0.037115982829

C -0.134009010502 -1.256661235896 0.013920778076

N -1.244626193605 -2.055553914785 -0.022226033323

N 0.905229426756 -2.139168510265 0.030367027719

C -0.903497211857 -3.400158706127 -0.027256805601

C 0.452745981577 -3.451306062280 0.004681046981

H -1.634224915621 -4.193113027520 -0.053006143506

H 1.119800291889 -4.298756043224 0.010715221829

C -2.615780146352 -1.523300655731 -0.044087800909

C -3.368481078975 -1.932164412813 -1.320927377451

C -3.397067030565 -1.905987100785 1.223778911027

H -2.495464251957 -0.430571279562 -0.056040934121

C -4.782706414401 -1.328846001409 -1.338642809673

H -3.438015793339 -3.028679348045 -1.366425334078

H -2.796016647538 -1.610741099574 -2.199167415958

C -4.811430252240 -1.303608587404 1.198243075516

H -3.465628349804 -3.001588660281 1.289710298087

H -2.844299615062 -1.566103776128 2.107686108690

C -5.573063117902 -1.702678923429 -0.075140333401

H -5.314673065599 -1.663439820342 -2.237410224769

H -4.706269873602 -0.234426900955 -1.409960790319

H -5.363366259412 -1.621024505145 2.091168182762

H -4.736832462956 -0.207974353960 1.249968250618

H -6.559263824542 -1.222973704296 -0.091191411295

H -5.752364673505 -2.788258511764 -0.066446547252

C 2.325770882050 -1.746713027311 0.067161450388

C 3.018894123431 -2.248816204459 1.344409093669

C 3.070350355150 -2.203513496696 -1.198123639347

H 2.311379277197 -0.656260261322 0.086095335539

C 4.484001513973 -1.782526935070 1.378456594036

H 2.983182081163 -3.347866201521 1.381508301961

H 2.474977630774 -1.880245232516 2.222575088841

C 4.537061840810 -1.743043433275 -1.157195050471

H 3.031621213032 -3.300298753968 -1.277071846687

H 2.563858775443 -1.798778492054 -2.082431986031

C 5.245220195618 -2.224110917159 0.118836881864

H 4.975014473182 -2.170407030219 2.279598586193

H 4.506369788583 -0.686390774302 1.451070028907

H 5.063423155802 -2.107835147123 -2.048029892178

H 4.565599422698 -0.645667270310 -1.198869783679

H 6.273487488368 -1.842590282046 0.146311094459

H 5.318667257195 -3.322223332898 0.104019539707

**TS3**

C 1.210969000000 1.940949000000 0.000007000000

C 1.915362000000 2.157666000000 1.206623000000

C 1.915376000000 2.157679000000 -1.206599000000

C 3.264652000000 2.514538000000 1.204913000000

H 1.406246000000 2.035518000000 2.158724000000

C 3.264666000000 2.514551000000 -1.204870000000

H 1.406271000000 2.035542000000 -2.158707000000

C 3.952087000000 2.691861000000 0.000027000000

H 3.780828000000 2.660864000000 2.151659000000

H 3.780854000000 2.660888000000 -2.151608000000

C -0.663940000000 2.464059000000 -0.000004000000

C -1.176133000000 2.986791000000 1.207312000000

C -1.176125000000 2.986780000000 -1.207329000000

C -2.185015000000 3.951285000000 1.206430000000

H -0.784104000000 2.635698000000 2.158247000000

C -2.185008000000 3.951273000000 -1.206463000000

H -0.784090000000 2.635677000000 -2.158258000000

C -2.695360000000 4.438911000000 -0.000020000000

H -2.570496000000 4.328017000000 2.151556000000

H -2.570483000000 4.327996000000 -2.151596000000

H 4.999548000000 2.981527000000 0.000034000000

H -3.471918000000 5.199486000000 -0.000027000000

Ni -0.158126000000 0.648206000000 -0.000002000000

C -0.231640000000 -1.286749000000 -0.000001000000

N -1.341966000000 -2.087226000000 0.000003000000

N 0.811283000000 -2.172343000000 -0.000004000000

C -0.996612000000 -3.431974000000 0.000003000000

C 0.360013000000 -3.484567000000 -0.000001000000

H -1.727159000000 -4.225625000000 0.000006000000

H 1.026301000000 -4.332950000000 -0.000002000000

C -2.714993000000 -1.562552000000 0.000005000000

C -3.478937000000 -1.966787000000 -1.271925000000

C -3.478936000000 -1.966795000000 1.271934000000

H -2.589745000000 -0.473081000000 0.000009000000

C -4.899001000000 -1.377430000000 -1.268322000000

H -3.539133000000 -3.063482000000 -1.330399000000

H -2.919728000000 -1.629661000000 -2.152920000000

C -4.898999000000 -1.377437000000 1.268336000000

H -3.539132000000 -3.063490000000 1.330402000000

H -2.919725000000 -1.629674000000 2.152931000000

C -5.671591000000 -1.771540000000 0.000007000000

H -5.438941000000 -1.707026000000 -2.164441000000

H -4.833652000000 -0.281495000000 -1.328116000000

H -5.438938000000 -1.707038000000 2.164454000000

H -4.833651000000 -0.281503000000 1.328136000000

H -6.663366000000 -1.302614000000 0.000009000000

H -5.839079000000 -2.859093000000 0.000004000000

C 2.223401000000 -1.758758000000 -0.000007000000

C 2.953301000000 -2.222023000000 1.271247000000

C 2.953297000000 -2.222031000000 -1.271261000000

H 2.184577000000 -0.665785000000 -0.000011000000

C 4.409356000000 -1.727206000000 1.268373000000

H 2.939098000000 -3.320753000000 1.328022000000

H 2.418707000000 -1.848103000000 2.152738000000

C 4.409353000000 -1.727213000000 -1.268394000000

H 2.939095000000 -3.320762000000 -1.328029000000

H 2.418701000000 -1.848117000000 -2.152753000000

C 5.154126000000 -2.172097000000 -0.000010000000

H 4.926674000000 -2.092102000000 2.164471000000

H 4.412522000000 -0.629735000000 1.325560000000

H 4.926669000000 -2.092114000000 -2.164491000000

H 4.412517000000 -0.629743000000 -1.325587000000

H 6.174474000000 -1.768709000000 -0.000013000000

H 5.250552000000 -3.268466000000 -0.000007000000

**PD (Ph–Ph)**

C -0.394757948026 1.139467714774 2.860343834479

C -0.395250321321 1.138752267292 1.465890963651

C 0.000000000331 -0.000000000088 0.742923804500

C 0.395250321637 -1.138752267440 1.465890963879

C 0.394757947717 -1.139467714795 2.860343834718

C -0.000000000276 0.000000000022 3.564412428718

H -0.712236566302 2.029415566130 3.397622694893

H -0.730168409839 2.021633662461 0.928428498407

H 0.730168410366 -2.021633662667 0.928428498828

H 0.712236565922 -2.029415566048 3.397622695336

H -0.000000000344 0.000000000060 4.651063171168

C 0.000000000405 -0.000000000106 -0.742923349842

C -0.395250320923 -1.138752267576 -1.465890508824

C 0.395250321470 1.138752267460 -1.465890508824

C -0.394757948342 -1.139467714941 -2.860343379663

H -0.730168409349 -2.021633662857 -0.928428043727

C 0.394757948181 1.139467715076 -2.860343379650

H 0.730168410106 2.021633662631 -0.928428043692

C -0.000000000285 0.000000000135 -3.564411973252

H -0.712236566761 -2.029415565930 -3.397622240651

H 0.712236566325 2.029415566129 -3.397622240687

H -0.000000000693 0.000000000277 -4.651062719766

Comparison of Transmetalation

**CP2-1_OTf**

Ni 0.700784469532 0.308259115696 0.383262102479

C 1.222186540331 -0.813060036598 1.804463586529

C 0.305079612686 -1.785686867711 2.238313427588

C 0.574949108280 -2.587672035764 3.354024694994

C 1.774524228382 -2.440409551825 4.052415269175

C 2.702726331589 -1.487361497333 3.625811152732

C 2.430974027934 -0.692250196681 2.508247562129

H -0.638027130695 -1.920792895456 1.713387838050

H -0.154080780382 -3.329398884738 3.674125288988

H 1.987251323264 -3.065725185800 4.915938043580

H 3.643102738120 -1.367002547381 4.160014937047

H 3.173990655042 0.037795995865 2.194131146120

Sn -4.123643034065 -0.083997613233 1.720771360594

C -2.271804557814 0.795378980769 2.409414568318

C -1.484009832680 1.564192589672 1.526000762867

C -0.276813990416 2.150118686044 1.939994837380

C 0.150909534670 2.000038394565 3.269186801956

C -0.617602603969 1.255170117232 4.155937334505

C -1.810525699428 0.655474500415 3.724956834277

H -1.840076864308 1.780551107772 0.524640486864

H 0.265881712163 2.795348924950 1.253308957496

H 1.073931735287 2.467190253749 3.601861890407

H -0.293893489347 1.133290415719 5.186435501383

H -2.387534373656 0.080047809674 4.446766420438

C 2.040015499224 -0.479697950226 -0.677787632009

N 3.185791383765 0.154744096825 -1.045019703392

N 2.082033841939 -1.690383421132 -1.286619546544

C 3.937425717776 -0.656221257536 -1.884459955110

C 3.245663775639 -1.813534895193 -2.032429823975

H 4.881435308340 -0.344844500742 -2.301632111139

H 3.476560193538 -2.699743917158 -2.600849232323

C 3.534969259560 1.537457621919 -0.669564045466

C 4.917047291096 1.611209835655 0.000314595630

C 3.439073330702 2.489024815462 -1.874416585355

H 2.772208828369 1.823550262426 0.063038890137

C 5.240072092312 3.055166615288 0.419396978806

H 5.685564498738 1.260897995360 -0.703493663394

H 4.947865818261 0.937577508959 0.864989130789

C 3.771911378624 3.930270620000 -1.455291285887

H 4.147012478728 2.160142768683 -2.649313744106

H 2.435199224610 2.433125675557 -2.304244333596

C 5.143425998069 4.023372802942 -0.769703949718

H 6.240416460371 3.093484267108 0.867223662668

H 4.535391221890 3.369496215254 1.203308899492

H 3.736575463542 4.585141286178 -2.333934121492

H 2.993926775705 4.292958319648 -0.767834118542

H 5.332712117188 5.050927127647 -0.435779382106

H 5.931299798138 3.780729976184 -1.498219659198

C 1.032644766394 -2.726664778227 -1.195019459261

C 0.498720306029 -3.105284694286 -2.585867990737

C 1.531550874079 -3.959194541839 -0.423588394249

H 0.223424087069 -2.250771477471 -0.637588090800

C -0.623571207644 -4.147657382837 -2.458248157723

H 1.314060735956 -3.525168330251 -3.193639426030

H 0.137192942139 -2.205431492587 -3.089804564371

C 0.413273995574 -5.008388448339 -0.307401617619

H 2.390925075637 -4.396779321249 -0.953049981825

H 1.879173350515 -3.655625763846 0.569360368889

C -0.158497341704 -5.389397446663 -1.681743082759

H -0.977125171461 -4.430496343136 -3.457197493400

H -1.478414014012 -3.687732379985 -1.943586364251

H 0.794776968981 -5.896881160872 0.210595542701

H -0.391018144128 -4.601918837846 0.322192182159

H -0.989659417078 -6.095586100188 -1.562268054741

H 0.615473896116 -5.912629036309 -2.263390975106

C -3.698260534525 -1.902719388998 0.654749796234

H -3.050624995253 -1.682833077449 -0.200059117367

H -3.197968232835 -2.632454080614 1.300656101189

H -4.623073169167 -2.355889108915 0.281599405430

C -5.091241474239 1.324849327411 0.423691583791

H -6.023717431762 0.907594144762 0.028923247375

H -5.329845637961 2.249874696198 0.958504230353

H -4.441806927052 1.571673468758 -0.422484741803

C -5.321044743016 -0.509178325140 3.459536557678

H -5.524450524402 0.403031519206 4.029992357596

H -6.281726783518 -0.944956666675 3.164971934609

H -4.820785264117 -1.222308522338 4.123697140228

O -1.330111926654 -0.398406310884 -1.833064733341

S -1.025815448442 1.035425750908 -2.005198845422

O -2.151975642078 1.973073490301 -2.063175846027

O 0.093859952628 1.512817025799 -1.088347610357

C -0.192569452604 1.153636718282 -3.664233100558

F -1.051041076777 0.849592446411 -4.640402479075

F 0.279027867904 2.391598965441 -3.868391363098

F 0.844002369710 0.295092815372 -3.723175220909

**TS2_OTf**

Ni 0.489109731178 0.048182198688 0.526548325481

C 1.165171063013 -0.802964642119 2.082906746068

C 0.846261864844 -2.118585736468 2.458343194581

C 1.391678632800 -2.711419115064 3.605139260695

C 2.292015126182 -2.005156816619 4.402594693308

C 2.630799446743 -0.697855355237 4.043736539637

C 2.071339359227 -0.108103843321 2.905975149099

H 0.158289673580 -2.707870553957 1.856694814281

H 1.111325786053 -3.729358249489 3.869836961474

H 2.720463911425 -2.462837165338 5.290731729941

H 3.324461479165 -0.127900828856 4.659159430725

H 2.330554054970 0.923251976036 2.676822657082

Sn -3.164130756812 -0.015776322787 0.665201662942

C -0.691325963476 1.248937158374 1.613454608859

C -0.798962394650 2.584058935037 1.141370686140

C -1.175949722403 3.651736997927 1.962129942627

C -1.489833200354 3.428761874229 3.304024320452

C -1.410767483438 2.129231044427 3.811926763291

C -1.013624095814 1.076724038563 2.984348842680

H -0.568977695424 2.805107768297 0.105721978132

H -1.228442372684 4.657711331324 1.550414947763

H -1.790365032988 4.254379947455 3.945056379229

H -1.643041149440 1.938056526998 4.857658261195

H -0.924097311099 0.091247919708 3.428150244046

C 1.885172414578 -0.888249881743 -0.500147780847

N 3.072120289686 -0.306049396251 -0.826333424667

N 1.932402548710 -2.113679549538 -1.089819352220

C 3.846586731989 -1.152521632494 -1.607893918846

C 3.129421078841 -2.291162870271 -1.773460123294

H 4.824138594820 -0.880629557254 -1.972889807542

H 3.364615054420 -3.195362580608 -2.311570607886

C 3.448856781429 1.063433078248 -0.439151827957

C 4.753233690039 1.083747787618 0.374055305760

C 3.521094199790 1.993263589313 -1.662421805365

H 2.630071685662 1.401632524008 0.204881243606

C 5.109613336017 2.519634487928 0.792847108691

H 5.571868385638 0.670571732645 -0.233086357877

H 4.643009998854 0.436568940388 1.251582814785

C 3.885598423603 3.424935867192 -1.236467444044

H 4.280991172620 1.615477025020 -2.362261056985

H 2.559027895566 1.974558872950 -2.185771854163

C 5.186174740779 3.461120568468 -0.419025288319

H 6.060142945165 2.519591861863 1.340146059425

H 4.346817460597 2.890336678636 1.493096568840

H 3.972151147036 4.063648074068 -2.123962169622

H 3.065365798968 3.839741486281 -0.632691526862

H 5.398221558200 4.485785053086 -0.089307911955

H 6.027013968112 3.158123547905 -1.060854870315

C 0.848114709404 -3.109066930831 -1.050354055601

C 0.262243253465 -3.362067886334 -2.450630801627

C 1.310448082084 -4.418448988262 -0.388326302823

H 0.075010252373 -2.648210098274 -0.427200954349

C -0.886363435799 -4.382001973968 -2.382293339390

H 1.053487992621 -3.750748546669 -3.108388223270

H -0.086244911785 -2.416918967762 -2.878063797313

C 0.156607702190 -5.432443700195 -0.322033536927

H 2.135445986486 -4.849317529071 -0.973935629882

H 1.703244164839 -4.204710697531 0.611590229000

C -0.449280725914 -5.692366632853 -1.709919575843

H -1.265771171127 -4.576252138586 -3.392682784505

H -1.722954902033 -3.944228630008 -1.817910976908

H 0.513097078391 -6.369028445134 0.123908313374

H -0.625171648200 -5.046745402115 0.349114237500

H -1.299924644861 -6.380620640475 -1.628987018136

H 0.298968695671 -6.192598176873 -2.342766513555

C -4.058913448454 -1.252855557456 -0.851219040565

H -3.294659937718 -1.787427690542 -1.424077827446

H -4.727089190023 -1.989049796249 -0.389374851188

H -4.628320014119 -0.640514385234 -1.556031768545

C -4.375729038562 1.639288173218 1.239741363045

H -5.274999192052 1.270716689570 1.746711565173

H -3.835026678178 2.303004168150 1.918062745204

H -4.674936923677 2.201174469411 0.351174609115

C -2.832041941491 -1.445133519293 2.249975471945

H -3.116855789435 -0.996370279008 3.205883201229

H -3.479978692107 -2.304514837497 2.042399505865

H -1.797838144275 -1.780744586914 2.320851183539

O -1.317010241971 -0.285660784757 -2.903600050537

S -1.185737151566 0.870315897128 -2.016602156935

O -2.366075707439 1.115593189054 -1.101466829329

O 0.109189807146 1.016903757409 -1.291691182287

C -1.225507238740 2.386060764616 -3.096157787139

F -2.332226809599 2.387307635725 -3.838671644448

F -1.194890351575 3.491984186997 -2.347710700110

F -0.154565645685 2.369826095342 -3.895790188403

**CP2-2_OTf**

Ni 0.979674683374 -0.124043645208 0.897533216996

C 1.954090169486 -0.884234272338 2.319942270596

C 1.698871126333 -2.175285798174 2.815163846730

C 2.483995369436 -2.745450707488 3.824792038770

C 3.550812737736 -2.033844462036 4.375673958454

C 3.813854848169 -0.743479780655 3.909398659672

C 3.025835009730 -0.180503767186 2.900306691469

H 0.864955031462 -2.754685733700 2.421515621647

H 2.255330676733 -3.747634715292 4.184129491008

H 4.160942922333 -2.472647308303 5.161528267333

H 4.631767252626 -0.167164258991 4.338843279707

H 3.244471744139 0.835618976932 2.577087631083

Sn -4.226876308939 0.739508953686 -0.087822703744

C -0.222277531130 0.638848606490 2.195108418937

C -0.405894421355 2.038396156514 2.194535061434

C -1.297116836575 2.679601070666 3.063318374914

C -2.058925480296 1.931954746595 3.966122652394

C -1.904859081678 0.542518123814 3.991962036439

C -0.993502062437 -0.080758244296 3.131109591553

H 0.156304505927 2.654729038486 1.493638143532

H -1.393811131284 3.764288042606 3.038948615116

H -2.744598124944 2.425063691972 4.652234958281

H -2.478510126515 -0.053846289544 4.700220340673

H -0.862680193872 -1.158376353896 3.214757097165

C 2.225372133595 -0.862125976902 -0.459851610626

N 3.256303987526 -0.190507208139 -1.048024513931

N 2.272750710178 -2.102441597106 -1.022441806869

C 3.933609197593 -0.995920693514 -1.954277224953

C 3.312821314888 -2.202270022349 -1.938029482603

H 4.784264529084 -0.653676896786 -2.522236621633

H 3.521710726128 -3.104371529951 -2.490942682213

C 3.578169901241 1.214569935630 -0.758198559311

C 4.995989111696 1.366137915183 -0.182839158480

C 3.368307590497 2.108271303564 -1.992359049150

H 2.854362742788 1.504568763109 0.011408487075

C 5.295811023225 2.838080660876 0.145116879233

H 5.730312839443 1.000595032631 -0.915652051967

H 5.091990152965 0.739107207254 0.710661639695

C 3.671990629418 3.578086143282 -1.659017255508

H 4.034060066863 1.773785609451 -2.801733792404

H 2.340474600045 1.993491614072 -2.354241891892

C 5.083924765129 3.746107734445 -1.076252333846

H 6.322506281981 2.932208080425 0.519449249549

H 4.635669909723 3.166663104933 0.960988049087

H 3.552375895546 4.194861580806 -2.558349771816

H 2.932490969872 3.941506359652 -0.930749906802

H 5.258906792044 4.794426842260 -0.803903045578

H 5.826765157549 3.494759475700 -1.848170781564

C 1.324882505714 -3.182601287428 -0.706782954315

C 0.472084678789 -3.560164601238 -1.930363881663

C 2.042747404163 -4.405689851092 -0.112163108634

H 0.668779877522 -2.751515944158 0.055907215977

C -0.526963634493 -4.673762692196 -1.575924827884

H 1.129761306239 -3.906657164350 -2.741262134139

H -0.055437672257 -2.671595638426 -2.293355934393

C 1.036675654672 -5.515207687436 0.234245644980

H 2.771092952060 -4.792314109221 -0.840119622997

H 2.602618764718 -4.097469771867 0.777372919022

C 0.176778838231 -5.901947436815 -0.978751613541

H -1.099323411641 -4.954822723514 -2.468675364517

H -1.255034340953 -4.283392688301 -0.849731403327

H 1.571220091687 -6.391311175964 0.621458461750

H 0.382587176182 -5.166259811212 1.046686826996

H -0.561968312549 -6.661719099291 -0.693734850930

H 0.817917212457 -6.361097875986 -1.746210801287

C -5.239518597586 0.605565642883 -1.961976504591

H -4.687876249693 -0.060646801661 -2.631382983230

H -6.249961100639 0.205169907762 -1.828154602247

H -5.316157892153 1.590239343716 -2.432106679556

C -4.822646155440 2.350642096693 1.161348336739

H -5.800699160395 2.149681488970 1.610991271140

H -4.083397357810 2.472561646134 1.958380844995

H -4.886297188695 3.278788914169 0.586164382424

C -3.848816028692 -1.126512031527 0.853251500711

H -4.777291395219 -1.532808033294 1.268702232002

H -3.441819082769 -1.832202603579 0.124895730013

H -3.126852403911 -0.983171782222 1.662002505788

O -1.638703395775 -0.578630639751 -1.820854266453

S -1.241729872359 0.769861290700 -1.409355271204

O -2.390941183551 1.508811600745 -0.689515574444

O 0.050803524597 0.974962320069 -0.739165315614

C -1.101473850976 1.803061562033 -2.951284880998

F -2.243132481189 1.728727342069 -3.641851315741

F -0.855796415450 3.072914296465 -2.635284334151

F -0.100489426913 1.331034970005 -3.698899747266
